# Diminished retinal complex lipid synthesis and impaired fatty acid **β**-oxidation associated with human diabetic retinopathy

**DOI:** 10.1172/jci.insight.152109

**Published:** 2021-10-08

**Authors:** Patrice E. Fort, Thekkelnaycke M. Rajendiran, Tanu Soni, Jaeman Byun, Yang Shan, Helen C. Looker, Robert G. Nelson, Matthias Kretzler, George Michailidis, Jerome E. Roger, Thomas W. Gardner, Steven F. Abcouwer, Subramaniam Pennathur, Farsad Afshinnia

**Affiliations:** 1 Department of Ophthalmology and Visual Sciences,; 2 Department of Molecular and Integrative Physiology,; 3 Michigan Regional Comprehensive Metabolomics Resource Core,; 4 Department of Pathology, and; 5 Department of Internal Medicine-Nephrology, University of Michigan, Ann Arbor, Michigan, USA.; 6 Chronic Kidney Disease Section, National Institute of Diabetes and Digestive and Kidney Diseases (NIDDK), Phoenix, Arizona, USA.; 7 Department of Statistics and the Informatics Institute, University of Florida, Gainesville, Florida, USA.; 8 Paris-Saclay Institute of Neuroscience, CERTO-Retina France, CNRS, Université Paris-Saclay, Orsay, France.; 9 Department of Internal Medicine-Metabolism, Endocrinology and Diabetes, and; 10 Michigan Regional Comprehensive Metabolomics Resource Core, University of Michigan, Ann Arbor, Michigan, USA.

**Keywords:** Ophthalmology, Diabetes, Fatty acid oxidation, Retinopathy

## Abstract

**BACKGROUND:**

This study systematically investigated circulating and retinal tissue lipid determinants of human diabetic retinopathy (DR) to identify underlying lipid alterations associated with severity of DR.

**METHODS:**

Retinal tissues were retrieved from postmortem human eyes, including 19 individuals without diabetes, 20 with diabetes but without DR, and 20 with diabetes and DR, for lipidomic study. In a parallel study, serum samples from 28 American Indians with type 2 diabetes from the Gila River Indian Community, including 12 without DR, 7 with mild nonproliferative DR (NPDR), and 9 with moderate NPDR, were selected. A mass-spectrometry–based lipidomic platform was used to measure serum and tissue lipids.

**RESULTS:**

In the postmortem retinas, we found a graded decrease of long-chain acylcarnitines and longer-chain fatty acid ester of hydroxyl fatty acids, diacylglycerols, triacylglycerols, phosphatidylcholines, and ceramide(NS) in central retina from individuals with no diabetes to those with diabetes with DR. The American Indians’ sera also exhibited a graded decrease in circulating long-chain acylcarnitines and a graded increase in the intermediate-length saturated and monounsaturated triacylglycerols from no DR to moderate NPDR.

**CONCLUSION:**

These findings suggest diminished synthesis of complex lipids and impaired mitochondrial β-oxidation of fatty acids in retinal DR, with parallel changes in circulating lipids.

**TRIAL REGISTRATION:**

ClinicalTrials.gov NCT00340678.

**FUNDING:**

This work was supported by NIH grants R24 DK082841, K08DK106523, R03DK121941, P30DK089503, P30DK081943, P30DK020572, P30 EY007003; The Thomas Beatson Foundation; and JDRF Center for Excellence (5-COE-2019-861-S-B).

## Graphical abstract

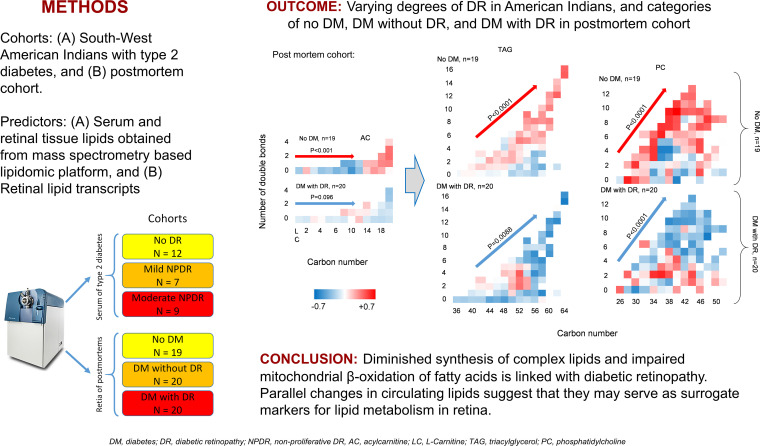

## Introduction

Diabetic retinopathy (DR) is one the most common and most devastating diabetes complications, with an estimated global prevalence of over 380 million individuals affected (1, 2). During the first 2 decades of disease, nearly all patients with type 1 diabetes and > 60% of patients with type 2 diabetes develop retinopathy. In the United States, approximately one-third of the patients aged 40 years or older with diabetes have DR, with 1 in 6 at risk of vision loss (3). The Diabetes Control and Complications Trial (DCCT) and Epidemiology of Diabetes Interventions and Complications (EDIC) trials have clearly demonstrated the need for intensive glycemic control as a key parameter to prevent or reduce the progression of complications (4). However, more recent studies have unveiled the important role of a variety of factors — including hypertension, inflammation, insulin resistance, and disordered lipid metabolism — in the generation and progression of multiple diabetes complications (5–7).

While these studies have shed some light on the pathogenesis of diabetic kidney disease and diabetic neuropathy, the metabolic basis of DR remains poorly understood. As for other diabetes complications, hyperglycemia is widely accepted as an important driver of DR in diabetes (8), but recent studies suggest a prominent role of altered lipid metabolism in DR pathology (9–15). In diabetic animal models, remodeling of fatty acids (15), alterations in 12/15-lipoxygenase (10), and a significant decrease in glycerophospholipids (12) have been reported in retina. Human studies also report alterations of erythrocyte phosphatidylcholines (PCs) (11) and circulating fatty acids and triglycerides (9) in association with DR; however, specific human retinal tissue analyses are still lacking. Lipidomic analysis of human kidney and peripheral nerve tissue have indeed revealed defective lipid metabolism (6, 7, 12, 16); however, lipidomic study of human retina remained limited exclusively to its surrogate assessment in the vitreous fluid of patients with advanced nonproliferative DR (NPDR) and proliferative DR, when those can be more easily collected (13). While interrogation of circulating lipids as surrogates of retinal metabolism may generate insight to retinal lipid metabolism, a more in-depth understanding of the retinal lipid metabolic alterations only comes from direct measurement of retinal lipids across various disease stages.

In this study, we take advantage of availability of serum biosamples from a very well phenotyped human cohort of patients with type 2 diabetes to identify the lipidomic signature associated with various stages of DR, in a similar fashion as we previously reported for diabetic kidney disease (6). This analysis at a systemic level was complemented by examination of the retina-specific lipidomic signature associated with DR postmortem eyes, allowing us to identify the lipidomic changes associated with NPDR in retinal tissues. Together, the results of this study demonstrate that markers of fatty acid β-oxidation and content in complex lipids are downregulated in retinal tissues, and that a specific panel of circulating lipids can discriminate patients without DR from those with various stages of DR.

## Results

### Baseline characteristics of Pima patients.

**Figure 1:**
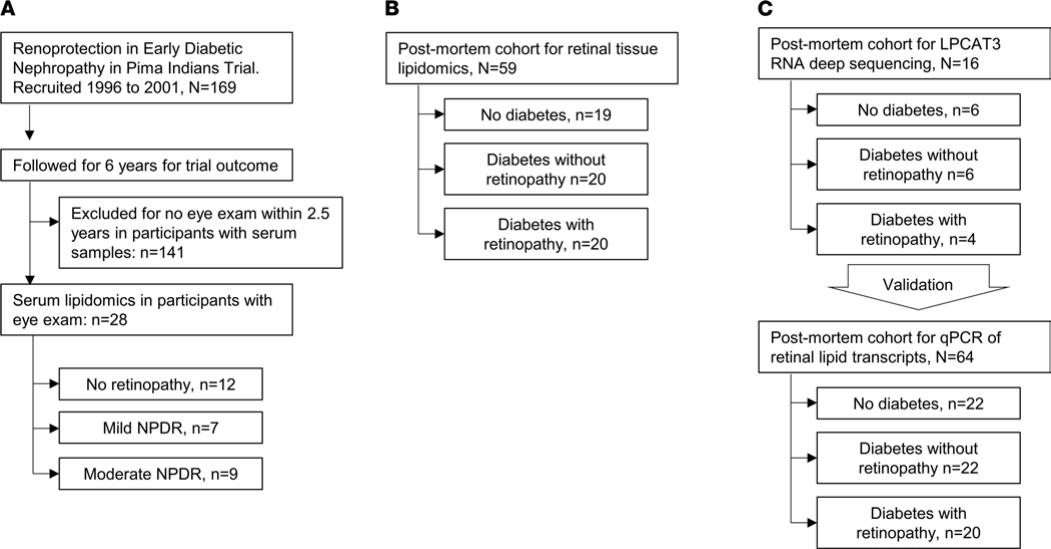
Flow diagram. (**A**–**C**) Flow diagram of Pima lipidomics (**A**), postmortem retinal tissue lipidomics (**B**), and postmortem retinal tissue transcriptomics studies (**C**).

Details of the American Indian study population and participant recruitment are published elsewhere (6, 17). Pima Indians are a highly homogeneous population among American Indians, with minimal interracial mix, who are in central Arizona (USA). High racial homogeneity minimizes the effect of other confounders on phenotypes of interest and, hence, make Pima Indians a unique opportunity to investigate biological processes. For these reasons, this population had been a target of highly granular observations over many years by NIH investigators, which led to collection of granular, well phenotyped, comprehensive clinical and outcome data, making it a very valuable population to investigate. The Pima study flow is shown in Figure 1A. From 169 Pima Indians from the Gila River Indian Community who were recruited for a randomized clinical trial (NCT00340678; ClinicalTrials.gov) between 1996 and 2001, 28 patients with available fasting serum samples at the end of the trial and eye examination within 2.5 years from the date of fasting serum sample collection were selected. The rest of the patients had eye examination on dates that were much farther from the index date of sample collection; therefore, they were not selected for the analysis. The individuals selected included 12 patients without DR, 7 with very mild (microaneurysms only) or mild NPDR, and 9 patients with moderate NPDR. Overall, the baseline characteristics of the patients in the 3 groups were similar except for a significantly higher level of urine albumin/creatinine ratio, a higher rate of insulin use, and no use of metformin in patients with moderate NPDR as compared with the other 2 groups (*P* ≤ 0.011; Table 1).

### Circulating lipids in Pima patients.

In serum samples, we measured 435 lipids from 18 classes including triacylglycerols (TAGs), diacylglycerols (DAGs), monoacylglycerols (MAGs), PCs, phosphatidylethanolamines (PEs), plasmenyl-PCs (pPCs), plasmenyl-PEs (pPEs), lyso-PCs (LPCs), lyso-PEs (LPEs), free fatty acids (FFAs), cholesteryl esters (CEs), phosphatidic acids (PAs), phosphatidylinositols (PIs), phosphatidylglycerols (PGs), phosphatidylserines (PSs), sphingomyelin (SM), ceramide-phosphates, and acylcarnitines (ACs) in positive and negative ionization modes (Supplemental Table 1; supplemental material available online with this article; https://doi.org/10.1172/jci.insight.152109DS1). After combining the different adducts of the same feature and eliminating duplicates, and the classes consisting of 2 or fewer lipid molecules (PA, PG, PS, pPC, ceramide phosphate, and MAG), 262 unique lipids including 16 FFAs (6.1%), 76 glycerolipids (29.0%), 109 phospholipids (41.6%), 12 CEs (4.6%), 20 SM (7.6%), and 29 ACs (11.1%) were included in the analysis.

**Figure 2:**
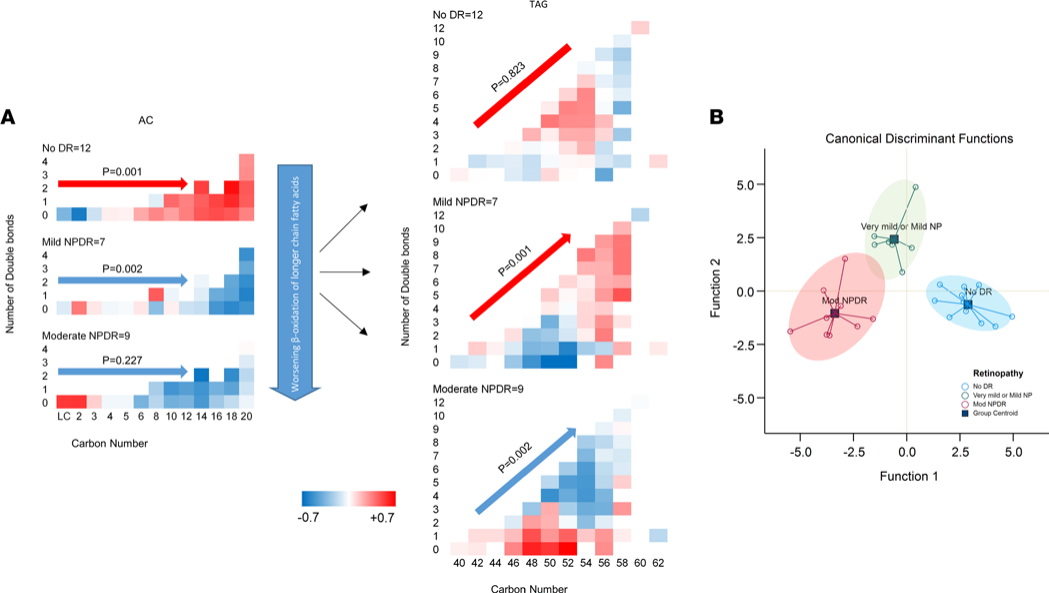
Comparing circulating lipids by diabetic retinopathy status. (**A**) Distribution of individual ACs and TAGs by retinopathy. Within each diagram, the *x* axis shows the number of carbons, the *y* axis shows the number of double bonds, and the color codes within each cell represent the *Z* score standardized mean abundance of the corresponding lipid. Statistical tests were mixed models (2-tailed *t* test) that tested the effect of the study group, carbon number, and number of double bonds as the main effects and their interaction; they also adjusted for urine albumin/creatinine ratio, use of metformin, and insulin. *P* values refer to significance of change in relative abundance of the corresponding lipid by increase in carbon number in ACs, as well as by increase in carbon number and number of double bonds (their interaction term) in TAGs. *n* for no DR, mild, and moderate NDPR is 12, 7, and 9, respectively. (**B**) Components of long-chain ACs (C ≥ 14) and intermediate-length unsaturated and monounsaturated TAGs accurately predicted the group without retinopathy (100%), mild NPDR (100%), and moderate NPDR (88.9%) with an overall accuracy of 96.4%. Statistical test was canonical discriminant analysis of components of differential lipid factors. *n* for no DR, mild, and moderate NDPR is 12, 7, and 9, respectively. AC, acylcarnitine; CE, cholesteryl ester; DAG, diacylglycerol; TAG, triacylglycerol; PC, phosphatidylcholine; PE, phosphatidylethanolamine; LPC, lyso-PC; LPE, lyso-PE; pPE, plasmenyl-PE; SM, sphingomyelin; sFFA, saturated free fatty acid; uFFA, unsaturated FFA; Sat Monounsat, saturated and monounsaturated; Polyunsat, polyunsaturated; Intm, intermediate; NPDR, nonproliferative diabetic retinopathy.

To reduce the data, minimize false discovery, and expand the variance coverage, components of lipid classes were reduced to class level of principal components representing the corresponding lipid classes. Accordingly, abundance of long-chain ACs (carbon number [C] ≥ 14) was significantly lower in patients with mild or moderate NPDR as compared with no retinopathy (*P* ≤ 0.029; Table 2). On the other hand, patients with moderate NPDR had the highest abundance of intermediate-length saturated and monounsaturated TAGs as compared with other groups, although only the comparison with mild NPDR reached statistical significance (*P* = 0.009; Table 2). Comparing the within-group components of the ACs and TAGs revealed a significantly higher abundance of long-chain ACs in patients without retinopathy versus shorter-chain ACs (*P* = 0.001, Figure 2A). Conversely, there was a lower abundance of long-chain ACs in both NPDR groups, this difference being highly significant in the mild NPDR group (*P* = 0.002; Figure 2A). Long-chain ACs were significantly lower in patients with DR compared with those without DR (*P* < 0.0001). A similar comparison of components of TAGs by study groups revealed a significantly higher abundance of longer-chain TAGs with higher number of double bonds in mild NPDR (*P* = 0.001), with reversal in abundance in patients with moderate NPDR (*P* = 0.002; Figure 2A). These associations were independent of urine albumin/creatinine ratio, use of metformin, or insulin. Critically, canonical discriminant analysis revealed that the components of long-chain ACs and intermediate-chain saturated and monounsaturated TAGs accurately classified all patients without retinopathy and mild NPDR, and 8 of 9 (88.9%) patients with moderate NPDR, with an overall model accuracy of 96.4% (*P* < 0.001, Figure 2B).

### Postmortem eye cohort.

The postmortem study flow for retinal tissue lipidomics is shown in Figure 1B. To study retinal lipid alterations by diabetes with and without retinopathy, we recovered ocular tissues from human cadavers within 9 hours of death in a postmortem cohort consisting of 19 nondiabetic donors, 20 participants with diabetes without retinopathy, and 20 diabetic donors with NPDR. All 3 groups were consistent regarding sex, age, and race (Table 3). As expected, hemoglobin A1c (HbA1c) was slightly higher in the DR group compared with the diabetic without DR (6.3% versus 8.1%, respectively) while diabetes duration was comparable in both groups (15.9 versus 13.4 years, respectively). Tissue from both the perimacular and peripheral retina was obtained for tissue lipidomic analysis.

### Postmortem retinal tissue lipids.

In postmortem retinas, we identified 1122 lipids from 25 classes including CE, bis-monoacylglycero phosphate (BMP), ceramide class consisting of α-hydroxy fatty acids and 4-sphingenines (Cer[AS]), Ceramides with dihydrosphingosine base (Cer[EODS]), ω-acylceramide (Cer[EOS]), ceramide consisting of nonhydroxy fatty acids and sphinganines (Cer[NDS]), ceramide consisting of nonhydroxy fatty acids (Cer[NP]), ceramides consisting of nonhydroxy fatty acids and 4-sphingenines (Cer[NS]), DAG, GlcCer[NS], LPC, LPE, PA, PC, PE, PG, PI, pPC, pPE, PS, SM, TAG, FFA, fatty acid ester of hydroxyl fatty acid (FAHFA), and ACs in both positive and negative mode (Supplemental Table 2). After combining the same adducts of different features, 632 unique lipids consisting of 10 CEs (1.6%), 16 BMPs (2.5%), 49 ceramides (7.8%), 47 fatty acids (7.4%), 336 phospholipids (53.2%), 38 SMs (6.0%), 107 glycerolipids (16.9%), and 29 ACs (4.6%) were included for the downstream analysis.

The identified tissue lipids are listed in Supplemental Table 2. After data reduction, similar to the above-mentioned procedure for serum samples, the lipids were reduced to 24 lipid classes as shown in Supplemental Table 3. Overall, FFAs, FAHFAs, DAGs, pPEs, PCs, ACs, and BMPs were more abundant in central retina, but PEs, LPCs, and Cer[NDS] were less abundant in the central retina (Supplemental Figure 1). The overall abundance of other lipids was not significantly different between central and peripheral retina in the nondiabetic or diabetic groups (Supplemental Figure 1). In central retina, the abundance of long-chain ACs (C ≥ 14), longer-chain FAHFAs, DAGs, TAGs, PCs, and Cer[NS] in the group with diabetes and retinopathy was significantly lower than the other 2 groups (participants with diabetes but without retinopathy, and participants without diabetes; *P* ≤ 0.0035; Supplemental Table 3). Tissues without diabetes had a significantly higher abundance of unsaturated FFAs (*P* = 0.0022) and PGs (*P* = 0.009) compared with the group with diabetes and retinopathy (Supplemental Table 3). Mean of CE was also lower in participants with diabetes with retinopathy, but the difference reached statistical significance only with the diabetic group without retinopathy (*P* = 0.008; Supplemental Table 3). In the peripheral retina, participants with diabetes and retinopathy had a higher mean of unsaturated FFAs and FAHFA compared with participants with diabetes without retinopathy (*P* < 0.0001), a lower TAG level when compared with no diabetes (*P* < 0.0001), and lower mean PC when compared with the other 2 groups (*P* ≤ 0.0011; Supplemental Table 3). There were no other significant differences in mean level of other lipids between the 3 groups in central or peripheral retina.

**Figure 3:**
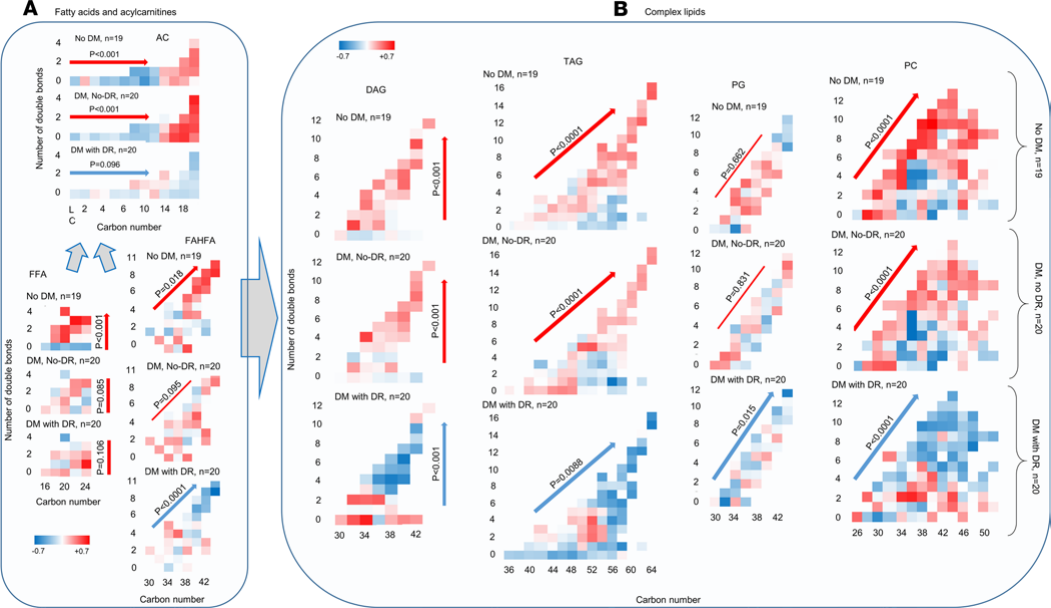
*Z* score–standardized mean relative abundance of FFAs, ACs, and complex lipids by carbon numbers and double bond numbers among postmortem groups. (**A**) Significantly higher abundant long chain ACs (C ≥ 14) in participants without diabetes and diabetics without retinopathy, higher unsaturated FFAs in participants without diabetes, and higher polyunsaturated longer-chain FAHFA in participants without diabetes, and their lower levels among diabetics with retinopathy. *P* values refer to significance of change in relative abundance of the corresponding lipid by increase in carbon number in ACs, by increase in unsaturated FFAs as compared with saturated FFAs in FFAs, and by increase in carbon number and number of double bonds (interaction term) in FAHFAs. (**B**) Significantly higher abundance of unsaturated DAGs, polyunsaturated TAGs, and PCs with higher carbon numbers in groups without diabetes and diabetics without retinopathy; however, a significantly lower abundance of unsaturated DAGs, polyunsaturated TAGs, PGs, and PCs is shown with higher carbon numbers in diabetes with retinopathy. Within each diagram, the *x* axis shows the number of carbons, and the *y* axis shows the number of double bonds. Statistical tests are based on mixed models tested the effect of study group, carbon number, and number of double bonds as the main effects and their interaction, adjusted for hypertension, hyperlipidemia, coronary artery disease, cancer, and kidney failure. *P* values refer to significance of change in relative abundance of the corresponding lipid by increase in number of double bonds in DAGs, and by increase in carbon number and number of double bonds (their interaction term) in TAGs, PGs, and PCs. *n* for both **A** and **B** is 19, 20, and 20 for participants without diabetes, diabetes with no DR, and diabetes with DR, respectively. AC, acylcarnitine; FAHFA, fatty acid ester of hydroxyl fatty acid; FFA, free fatty acid; DAG, diacylglycerol; TAG, triacylglycerol; PG, phosphatidylglycerol; PC, phosphatidylcholine; DM; diabetes mellitus; NPDR, nonproliferative diabetic retinopathy.

**Figure 4:**
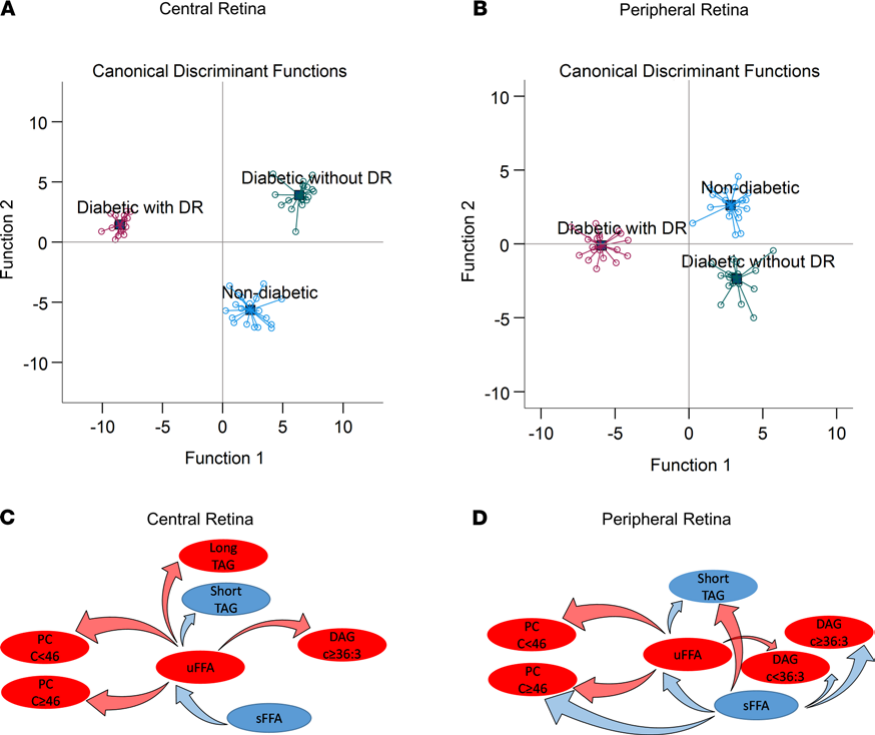
Group discrimination by retinal lipids in a postmortem cohort. (**A** and **B**) Top differentially regulated lipids, triglycerides, phosphatidylcholine, and ACs in central retinal (**A**), and triglycerides and phosphatidylcholines in peripheral retina (**B**) discriminated 3 study groups. Analyses are based on canonical discriminant analysis with 100% accurate discrimination in central retina, and 98.3% in peripheral retina. (**C** and **D**) Statistically significant correlates of saturated and unsaturated fatty acids in the central and peripheral retina of postmortem cohort. Lipids in red indicate elevated levels; lipids in blue indicate suppressed levels; arrows in red indicate statistically significant direct correlation; arrows in blue indicate statistically significant inverse correlation. Overall saturated FFAs are inversely correlated with unsaturated FFAs. A higher abundance of unsaturated FFAs suggest shift of sFFAs toward uFFAs via action of desaturases. Direct correlation of uFFAs with complex glycerolipids suggests their higher incorporation in the construct of glycerolipids, while inverse correlation of sFFAs with complex lipids suggest their relatively lower incorporation in the construct of complex lipids. The net effect would be a relatively higher abundance of complex lipids in association with a higher abundance of uFFAs under normal physiological conditions. With progression toward diabetic retinopathy, diminished levels of uFFAs leads to diminished tissue levels of glycerophospholipids. *n* for all panels is 19, 20, and 20 for participants without diabetes, diabetes with no DR, and diabetes with DR, respectively. AC, acylcarnitine; sFFA, saturated free fatty acids; uFFA, unsaturated free fatty acids; DAG, diacylglycerols; PC, phosphatidylcholines; TAG, triacylglycerols.

Comparing the constituents of fatty acids and ACs of central retina among the 3 study groups revealed a significantly higher abundance of long-chain ACs (C ≥ 14) as compared with shorter-chain ACs (C < 14) in patients without diabetes or with diabetes without retinopathy (*P* < 0.001; Figure 3A), but the dominant abundance of long-chain AC disappeared in the groups with retinopathy. Participants without diabetes had a higher abundance of unsaturated FFAs compared with saturated FFAs (*P* < 0.001), but the difference disappeared in the other 2 groups with diabetes (Figure 3A). The abundance of longer polyunsaturated FAHFA was higher than the number of double bonds, and carbon numbers increased in participants without diabetes (*P* = 0.001), was not significantly different in participants with diabetes without retinopathy, and was significantly lower in participants with diabetes with retinopathy (*P* < 0.0001; Figure 3A). Comparing the abundance of complex lipids constituents by carbon number and number of double bonds showed a significantly higher abundance of unsaturated DAGs in patients without diabetes or without retinopathy, but it showed a significantly lower abundance in participants with diabetes with retinopathy (Figure 3B). The nondiabetic group and the group with diabetes but without retinopathy exhibited a significantly higher abundance of TAGs and PCs by increase in carbon number and number of double bonds, while the group with diabetes and retinopathy showed a significant decrease in their abundance by increase of carbon number and number of double bonds (*P* < 0.0001; Figure 3B). Similarly, the diabetes group with retinopathy exhibited a significantly lower abundance of PG by an increase in carbon number and number of double bonds (*P* = 0.015; Figure 3B), but the differences in various PGs were not significant in the other 2 groups. These associations were independent of comorbid conditions. Canonical discriminant analysis using the top differentially regulated lipid classes including TAGs, PCs, and ACs in central retina (Figure 4A), and TAGs and PCs in peripheral retina (Figure 4B), discriminated the 3 groups with 100% (Figure 4A) and 98.3% (Figure 4B) accuracy.

### Retinal tissue RNA deep sequencing.

**Figure 5:**
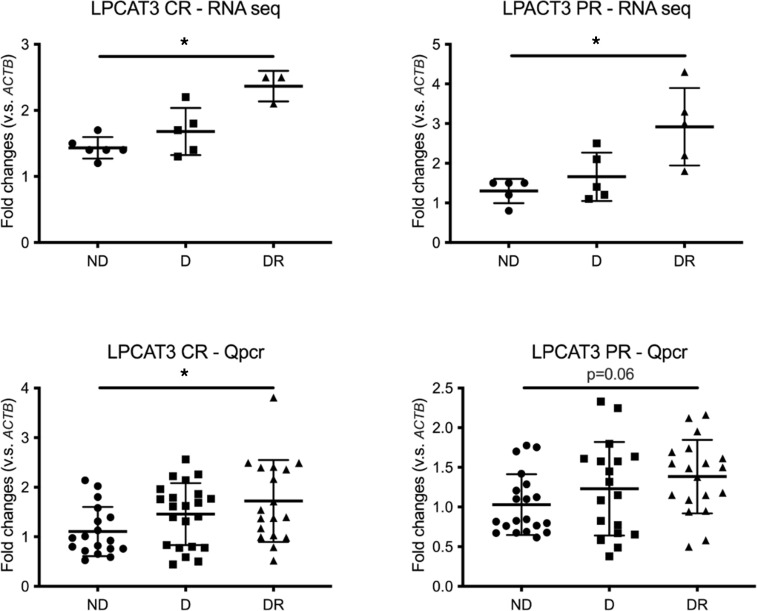
RNA deep sequencing and qPCR. Relative expression of the lipid metabolism gene *LPCAT3* in the central (left; CR) and peripheral (right; PR) retina, analyzed by RNA deep sequencing (top) or qPCR (bottom). Similar changes were observed by both methods. In the top panels, *n* is 6, 6, and 4 for participants without diabetes, diabetes without DR, and diabetes with DR, respectively. In the bottom panel *n* is, 22, 22, and 20 for participants without diabetes, diabetes without DR, and diabetes with DR, respectively. qPCR *P* values are adjusted by sex, hypertension, hyperlipidemia, coronary artery disease, nephropathy, and cancer. The statistical test was ANOVA. Data are shown as mean ± SD (*P < 0.05 in DR versus ND).

**Figure 6:**
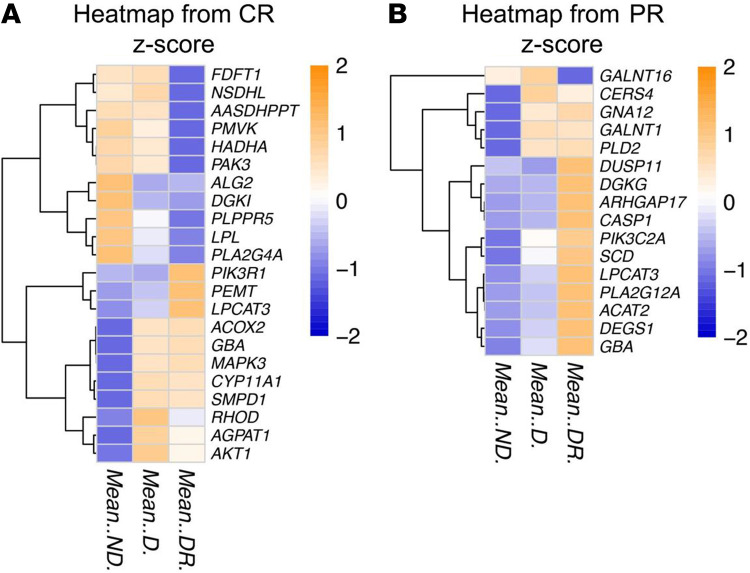
Comparing lipid-related gene expression in central and peripheral retina by study groups. (**A** and **B**) Relative expression (*Z* score) of the lipid metabolism–related genes significantly affected by diabetes and DR in the central (left; CR) and peripheral (right; PR) retina, analyzed by RNA deep sequencing. *n* for both panels is 6, 6, and 4 for participants without diabetes, diabetes without DR, and diabetes with DR, respectively.

The postmortem study flow for retinal tissue RNA deep sequencing and quantitative PCR (qPCR) of retinal lipid transcripts is shown in Figure 1C. Analysis of RNA deep sequencing in a group of human retinal samples showed significantly higher expression of LPCAT3 in association with DR both in central and peripheral retina (Figure 5, top panel). To validate the fold change alterations in a larger and independent set of donor samples obtained from 22 postmortem donors without diabetes, 22 with diabetes but without retinopathy, and 20 with diabetes and retinopathy (Table 4), qPCR analysis was performed for multiple genes, including LPCAT3, which showed the same direction alterations in both the central and peripheral retina, although statistical significance of LPCAT3 fold change in peripheral retina mitigated to borderline after multivariate adjustment for other covariates (Figure 5, bottom panel). Comparing the *Z* score standardized relative expression of the genes involved in lipid metabolism in central retina (Table 4) reveals differential expression of genes involved in de novo biosynthesis of fatty acids (*PLA2G4A*), their elongation (*HADHA*), their β-oxidation (*ACOX2*), metabolism of complex lipids (*AASDHPPT*, *AGPAT1*, *CYP11A1*, *DGKI*, *FDFT1*, *GBA*, *LPL*, *NSDHL*, *PEMT*, *PIK3R1*, *SMPD1*, *LPCAT3*, *PLPPR5*), and others/housekeeping genes (other central retina genes from Table 5). Particularly striking is the downregulation of *PLA2G4A* (fatty acid biosynthesis), *HADHA* (elongation), and *AASDHPPT*, *DGKI*, *FDFT1*, *LPL*, *NSDHL*, and *PLPPR5* (complex lipid metabolism) and the upregulation of *ACOX2* (β-oxidation) and *CYP11A1*, *GBA*, *MAPK3*, *SMPD1*, and *LPCAT3* (specific lipid species metabolic genes). Corresponding changes in lipid transcripts in peripheral retina are shown in Table 6. These data demonstrate that diabetes affects lipid metabolism in a regional manner, a phenomenon highlighted by the heatmap representation of the relative expression and changes of these genes in the RNA deep sequencing (*Z* score; Figure 6), providing important information on the potential mechanistic role of these perturbations in the onset and progression of DR. Indeed, DR has profound early effects on the peripheral retina (18, 19).

### Independent correlates of FFAs.

The partial correlation coefficients of the ACs, FFAs, and complex glycerolipids with significant alteration in central and peripheral retina are shown in Supplemental Table 4. Saturated FFAs are inversely correlated with unsaturated FFAs. Unsaturated FFAs are also directly correlated with longer-chain DAGs, longer-chain TAGs, and PC; however, they are inversely correlated with shorter chain TAGs in central retina. Similarly, unsaturated FFAs are directly correlated with shorter-chain DAGs and PCs, but inversely correlated with shorter TAGs in peripheral retina. In peripheral retina, saturated FFAs are directly correlated with shorter TAGs, and they are inversely correlated with DAGs, unsaturated FFAs, and longer-chain PCs (Figure 4, C and D).

## Discussion

**Figure 7:**
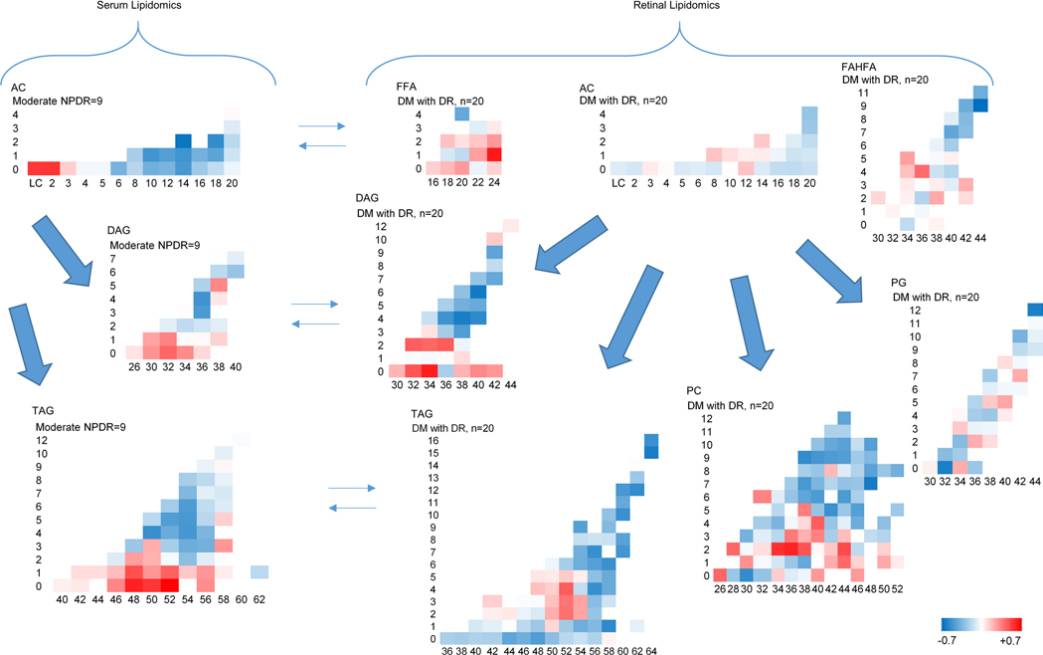
Similar lipid alterations in serum and retinal suggesting parallel changes in the 2 compartments. Left panel shows the serum lipidomics in moderate nonproliferative diabetic retinopathy (*n* = 9), and right panel shows central retinal lipidomics in diabetes with retinopathy (*n* = 20). The *x* axis in all panels represents carbon number, and the *y* axis represents the number of double bonds.

In this study, we found a significantly lower abundance of long-chain ACs (C ≥ 14), longer-chain FAHFAs, DAGs, TAGs, PCs, and Cer[NS] at lipid class level in central retinal tissue obtained postmortem from patients with DR than from those without diabetes or from those with diabetes who did not have DR. These differences in abundance accurately discriminated the 3 study groups. In an independent cohort of Pima Indians with type 2 diabetes, a similar graded decrease in abundance of circulating ACs and polyunsaturated TAGs was observed by worsening status of DR, from no DR to moderate NPDR. The panel also discriminated the 3 subgroups with high accuracy. Our findings suggest diminished synthesis of retinal lipids and impaired mitochondrial β-oxidation of retinal fatty acids in the retina in DR. Changes in a similar panel of circulating lipids mirror the lipid alterations occurring in the retina (Figure 7). The clinical implication of these findings includes lipid metabolic alterations as potentially novel risk factors for worsening of DR, optimization of systemic or retinal lipid metabolic derangements as potentially novel therapeutic targets, and availability of circulating biomarkers as surrogates of similar changes in retina.

In diabetic rat models, Tikhonenko et al. (15) reported a decrease in retinal long-chain-to-short-chain polyunsaturated fatty acids (PUFA) ratio; decreased abundance of glycerophospholipids containing DHA in PE, PC, and PS lipid classes; and significant reduction in *Elovl4* and *Elovl2* retinal elongase gene expression. They also reported a tendency toward higher total plasma fatty acid level in diabetic versus control groups, as well as a significant decrease in arachidonic acid and DHA abundance the 2 major end products of the PUFA synthesis pathway. In another study of the *db/db* mouse model, Sas et al. (12) showed a significant reduction in retinal complex lipids in the diabetic mice after systemic infusion of labeled palmitate. In a lipidomic study of human erythrocytes’ phospholipids, Koehrer et al. (11) reported a higher level of PC and plasmenyl-choline in diabetic patients without retinopathy. In a study of 648 individuals with type 1 diabetes, 2,4-dihydroxybutyric acid (DHBA); 3,4-DHBA; ribonic acid; and ribitol were positively — and the triglycerides 50:1 and 50:2 were negatively — correlated with DR stage (9). Overall, our findings are aligned with these other studies that report a lower abundance of complex retinal lipids accompanying DR.

In this study, we found a higher abundance of FFAs, FAHFAs, DAGs, pPEs, PCs, ACs, and BMPs, but we found a lower abundance of PEs, LPC, and Cer[NDS] in the central retina. In mammalian cells, PCs are made by 2 biosynthetic pathways, including the Kennedy pathway and by conversion from PE (20). In the Kennedy pathway, phosphocholine transfers from CDP-choline to DAG by endoplasmic reticulum membrane proteins to produce PCs. In the other PC biosynthetic pathway, PE is converted to PC by 3 successive methylation reactions catalyzed by phosphatidylethanolamine N-methyltransferase (PEMT) using S-adenosylmethionine as the methyl-group donor (20). PCs may further convert to LPCs via action of phospholipase a1 and a2 (PLA1,2) (21). These findings suggest that, compared with peripheral retina, a relatively higher abundance of FFAs and FAHFAs promotes their incorporation in construction of regional DAGs and PCs, leading to their higher abundance in the central retina. On the other hand, lower PEs and LPCs in the central retina may reflect lower conversion of PE to PC and PC to LPC, respectively, compared with peripheral retina. Differential lipid metabolism by retinal regions may be due to differences in cell mitochondrial contents, vascularization ratio, and density of ganglion cells, leading to a lower lipid metabolic rate in peripheral retina.

In the parallel transcriptomic analysis, we found that, in the central retina of patients with DR, genes involved in de novo biosynthesis of fatty acids (*PLA2G4A*), fatty acid elongation (*HADHA*), cholesterol, and glycerophospholipid metabolism were downregulated — except for *SMPD1* and *LPCAT3*, which were upregulated. On the other hand, in the peripheral retina, only *GALNT16* involved in sphingolipid metabolism was downregulated, while other genes involved in lipid metabolism were upregulated. The transcriptomic gene expressions suggest decreased systemic and retinal de novo biosynthesis of fatty acids and their elongation, along with diminished incorporation into complex lipids. In a mouse model experiment, Rajagopal et al., showed that deletion of the de novo lipogenic enzyme fatty acid synthase from neural retina resulted in progressive neurodegeneration and blindness, a phenomenon that was associated with decreased membrane cholesterol content, as well as loss of discrete n-3 polyunsaturated fatty acid– and saturated fatty acid–containing phospholipid species within specialized membrane microdomains (22). The gene expression findings are aligned with differential abundance of fatty acids and complex lipids observed as a function of DR. This suggests that the underlying mechanisms for retinal differential lipid alterations may involve general reduction in central retinal lipid synthesis and downregulation of retinal elongases, leading to diminished synthesis of longer fatty acids and their decreased incorporation in longer polyunsaturated complex lipids (5), along with compensatory upregulation of SMPD1 and *LPCAT3*. In parallel, diminished cytosolic long-chain ACs contribute to inefficient mitochondrial transfer of long-chain fatty acids, resulting in their incomplete mitochondrial β-oxidation (5). In this context, the upregulation of *ACOX2* in the central retina is likely a compensatory mechanism aimed at overcoming this inefficient β-oxidation. Incomplete β-oxidation of fatty acids, when coupled with diminished synthesis of complex lipids, may further promote progressive degenerative processes (5). Alternatively, our data point to other potential disease mechanisms, including PA-mediated reduction in mTOR signaling promoting increased cell death (23), and glucosylceramide- and glycosphingolipid-mediated worsening of local insulin resistance and cell death (24). Decreased retinal long- and very long–chain polyunsaturated fatty acids can also compromise integrity of photoreceptor outer segments (25).

This study has several strengths. The approach to tissue lipid identification and quantification in a postmortem tissue is potentially novel and provides valuable insights into the pathophysiology of disease. The Pima cohort is also a very well phenotyped cohort of patients with type 2 diabetes with high-quality clinical data and biosamples that provided the opportunity for parallel investigation of circulatory lipids. A major strength of this study is alignment of circulatory lipid alterations with the retinal lipids, suggesting that a similar change in circulatory lipids may be used as a surrogate of retinal tissue lipid metabolic derangements. The lipidomic platform provides high-quality data, with low coefficients of variation and minimal or no batch-to-batch variability. The platform also enabled us to identify a large array of lipids from various classes with various chain lengths and saturation status, and this contributes to a highly granular data structure.

This study also has limitations. The observational nature of the study does not allow inferring causality. However, when these observational data are coupled with existing established lipid biology knowledge, important insight into pathophysiology is gained. The cross-sectional nature of data collection precludes a flux analysis and, therefore, relative contribution of de novo lipogenesis and its products percentage cannot be determined. The sample size in Pima Indians is relatively small, and 3 different cohorts contributed to various samples. However, convergence of the findings by 3 different cohorts toward common lipid pathways points to a consilience that argues for validity of the findings, as opposed to chance findings. Like other omics-type studies, our lipidomic platform generated a large array of lipids. Statistical approaches relying on individual lipid alterations suffer from high FDR. However, we applied several data-reduction strategies, including principal component analysis and mixed models, to reduce the large number of lipids to a smaller number of lipid classes. These strategies reduced the risk of false discovery and increased the covered variance by the lipid factors; hence, they optimized alignments with the study phenotypes. Similarly, the integrative analysis applied the partial correlation–based sparsing technique with potential to minimize false discovery. Fasting was not applicable to postmortem cohorts; however, unlike circulating levels, retinal tissue lipids are unlikely to undergo rapid alteration in lipid content attributed to fasting. In the postmortem study, there is potential for lipid and transcript alteration attributed to body degradation. However, every single sample was quality controlled by TapeStation and gel analysis, and each showed a similar profile when compared with samples obtained from the operating room. This is consistent with prior studies that reported stability of postmortem lipids and transcripts with negligible changes attributed to autolysis within 24 hours following death (26, 27). The retinal tissues were retrieved within 9 hours after death, according to a research protocol that was applied uniformly to all 3 study groups, besides the highly significant effect size attributed to study groups; therefore, the potential for biased lipid estimates in the postmortem cohort is quite low. Although mRNA alterations are informative, they may not necessarily correlate with enzyme activity; therefore, along with replication of the lipidomic findings in more diverse ethnic groups, further mechanistic studies will be required to assess alterations in enzyme activity. However, findings in the Pima cohort have characteristically been confirmed in other populations.

These findings have important clinical implications. In a systematic review of randomized controlled clinical trials, the effect of statins on DR was uncertain, but fibrates reduced the incidence of macular edema by 45% (28). Our results illustrate widespread retinal lipid alterations that span a wide array of lipids representative of metabolic pathways above and beyond the cholesterol synthesis pathway. Furthermore, while DR is known to be driven by hyperglycemia, marked lipid alterations associated with DR — including downregulation of complex lipid contents in retina, along with impaired β-oxidation of fatty acids — highlight a significant derangement in retinal lipid metabolism. These findings suggest that altered retinal and systemic lipid metabolism might be a novel risk factor for development or worsening of DR, and that novel targeted interventions aimed at optimizing systemic or retinal lipid metabolism might have additive clinical outcome benefits above and beyond optimal glycemic control. Further research is required to determine if such targeted interventions might mitigate or prevent risk of DR in diabetes.

In conclusion, our findings suggest diminished elongation and desaturation of fatty acids associated with a worsening stage of DR. Associated low abundance of complex lipids and long-chain ACs also suggest diminished incorporation of longer and polyunsaturated fatty acids in newly synthesized complex lipids, and their diminished mitochondrial β-oxidation, respectively.

## Methods

### Cohort selection.

Details of the American Indian study population and participant recruitment are published elsewhere (6, 17). In brief, Pima Indians from the Gila River Indian Community participated in a longitudinal study of diabetes and its complications. In total, 169 of the study participants were recruited between 1996 and 2001 for a randomized, double-blind, placebo-controlled clinical trial to assess the efficacy of the angiotensin receptor blocker losartan on onset and progression of diabetic nephropathy in type 2 diabetes (NCT00340678). The Pima study flow is shown in Figure 1A. Of 169 participants in the clinical trial, 28 patients who provided serum samples at the end of the trial and had a retinal examination within 2.5 years of that sample collection were selected for lipidomic analysis. Retinal photographs were taken using the Joslin Vision Network–Indian Health Service (JVN-IHS) protocol described in detail elsewhere (29). In brief, the JVN-IHS program uses a 45° field of view low-illumination nonmydriatic digital imaging system to obtain retinal images. Five photographs are taken per eye (three 45° images and two 30° images) with grading undertaken at the JVN-IHS reading center in Phoenix (Arizona, USA). Retinopathy grades are based upon the gold-standard Early Treatment Diabetic Retinopathy Study (ETDRS) grading (30) into the categories of no retinopathy; NPDR was graded as minimal, mild, moderate, severe, or very severe; and proliferative DR was graded as quiescent, less than high-risk, and high-risk. For this study, the retinopathy grade was based on the finding in the worst eye at the retinal examination closest to the date of serum collection for the lipid analysis. Sex was self-reported in all participants.

### Postmortem eye selection and ascertainment of retinopathy.

Ocular tissues were recovered from human cadavers within 9 hours of death and immediately frozen until processing from Eversight (Ann Arbor, Michigan, USA). Fragments of central (perimacular) and peripheral retina were isolated as done previously (31). Central retina is defined as the approximate equivalent of the standard 7-field photography region based on the optic nerve head, macula, and retinal vasculature architecture. Retinal punches were subsequently processed for untargeted lipidomic analysis by mass spectrometry (MS; see below for details of methods), RNA deep sequencing, or qPCR. At reception of the samples, retinopathy grading was confirmed by fundus photographs and optical coherence tomography performed in the laboratory. None of the donors had proliferative retinopathy, vitreous hemorrhage, or diabetic macular edema. For the lipidomic analysis, tissues were obtained from 19 nondiabetic donors, 20 participants with diabetes without retinopathy, and 20 diabetic donors with NPDR. For the RNA deep sequencing analysis, tissues were obtained from 6 nondiabetic donors, 6 participants with diabetes without retinopathy, and 4 diabetic donors with NPDR, and the results were validated by qPCR on tissues from 22 nondiabetic donors, 22 participants with diabetes without retinopathy, and 20 diabetic donors with NPDR. The groups were balanced as best as possible by race, sex, and age. Diabetes duration was comparable with no statistically significant difference between the diabetic donors with and without retinopathy, but HbA1c was higher in donors with DR as compared with donors without DR for the lipidomics study (*P* = 0.0176; Table 3). Males were slightly overrepresented for the transcriptomic analysis (*P* = 0.0332; Table 4). Sex and race were self-reported in all participants.

### Sample preparation and MS.

The details of sample preparation and MS are presented elsewhere (6, 32, 33). In brief, 10 μL of serum sample or 5 mg of sonicated retinal tissue were added to water/methanol/dichloromethane at room temperature with 2:2:2 volume ratio, followed by spiking internal standards PC17:0/17:0, LPC 17:0, PG 17:0/17:0, PE 17:0/17:0, TAG 17:0/17:0/17:0, SM 18:1/17:0, MAG 17:0, DAG 16:0/18:1, CE 17:0, ceramide d18:1/17:0, PA 17:0, PI 17:0/20:4, and PS 17:0/17:0. After collecting the organic layer, the extracts were dried under nitrogen and reconstituted by adding 100 μL of acetonitrile/water/isopropyl alcohol (10:5:85) followed by 10 mM ammonium acetate (NH_4_OAc). Then, the extracts were examined by liquid chromatography–MS (LC/MS), utilizing ABSciex quadrupole TOF-5600 mass spectrometer equipped with a Turbo VTM ion source (AB Sciex) and Shimadzu CTO-20A Nexera X2 UHPLC with water acquity UPLC HSS T3 1.8 μm column (Waters). ACs were quantified by LC/MS using an Agilent 6410 triple-quadrupole tandem mass spectrometer (Agilent) with a targeted method described previously (7).

### Quality control.

We injected a pool of study samples at the beginning and after every 20 runs in the lipidomics study and after every 10–15 runs in the AC study. The low coefficients of variations and minimal batch-to-batch variation are shown in Supplemental Figures 2–5.

### Expression analysis.

Total RNA was purified with an RNA preparation kit (RNeasy Plus Mini Kit; Qiagen) and a homogenizer (QIAshredder; Qiagen). Paired-end RNA deep sequencing analysis was performed by the University of Michigan DNA genomic core following quality assessment using the TapeStation system (Agilent). qPCR and duplex qPCRs were performed as previously described (34) using gene-specific primers and fluorescent dye–labeled probes (Applied Biosystems Life Technologies). Reactions were performed and monitored using a real-time PCR system (CFX384; Bio-Rad). Relative normalized mRNA levels were calculated using the ΔΔCt method.

### Statistics.

Mean ± SD and count (%) were used for description of normally distributed continuous and categorical variables, respectively. Median ± IQR was applied for description of skewed variables. We used 1-way ANOVA with Bonferroni post hoc analysis to correct for multiplicity to compare mean values of normally distributed continuous variables and the Kruskal-Wallis test to compare distribution of skewed continuous variables across the 3 groups. We used 2-tailed *t* tests to compare duration of diabetes in participants with and without diabetes or mild and moderate NPDR. We applied χ^2^ to test the association of categorical variables with study groups. The lipidomic data were intraclass lipid sum normalized, logit transformed, and *Z* score standardized for the downstream analysis. We used mixed models corrected for acyl chain carbon content and number of double bonds to compare intraclass lipid mean values across study groups. To explore the effect of potential confounders, the models were also adjusted by the imbalanced background covariates, followed by stepwise deletion of nonsignificant variables from the models. To reduce the intraclass lipids into subclasses, we further applied principal component analysis with varimax orthogonal rotation. To identify the topology of lipid classes, we generated a group-adjusted partial correlation matrix. We applied canonical discriminant analysis to separate study groups by proposed lipid markers. As the findings are stemmed on lipids as class rather than individual lipids, the power analysis was performed for discriminant analysis using the canonical discriminant functions separating the study groups. Accordingly, the discriminant analysis with 2 canonical discriminant functions in 12 participants without retinopathy, 7 with mild NPDR, and 9 with moderate NPDR in the Pima cohort with 96.4% model accuracy achieved over 95% power to discriminate study groups with 4 degrees of freedom using a χ^2^ test at α of 0.01. Similarly, the discriminant analysis with 2 canonical discriminant functions in 19 participants without diabetes, 20 with diabetes but without DR, and 20 with DR in a postmortem cohort with 100% model accuracy achieved over 95% power to discriminate study groups with 4 degrees of freedom using a χ^2^ test at α of 0.01.

### Study approval.

The study was approved by the IRB 0000006 at the NIDDK. All participants gave signed informed consent prior to their participation in the study.

## Author contributions

PEF led the design of the postmortem study, postmortem data, and sample collection; performed the transcriptomic experiments; and contributed to drafting the manuscript. TMR led the lipidomic MS and contributed to drafting the manuscript. TS performed the MS lipidomic data acquisition and normalization, and contributed to drafting the manuscript. JB led the MS AC measurement and contributed to drafting the manuscript. YS helped with retinal tissue collection and transcriptomic study. HCL contributed to conceptual design, execusion of the Pima study, drafting the manuscript, and critical revision of the manuscript. RGN contributed to conceptual design, execusion of the Pima study, drafting the manuscript, and critically revising the manuscript. MK contributed to conceptual design. GM contributed to data analysis and drafting the manuscript. JER critically evaluated the manuscript. TWG contributed to conceptual design, funding, drafting the manuscript, and critical evaluation of the manuscript. SFA ascertained DR stages in Pima Indians and helped with drafting the manuscript. SP contributed to conceptual design, funding, and critical evaluation of the paper. FA generated the lipidomic data in Pima and the AC data in both cohorts, performed the statistical analysis, and wrote the first draft of the manuscript.

## Supplementary Material

Supplemental data

Trial reporting checklists

ICMJE disclosure forms

## Figures and Tables

**Table 1:**
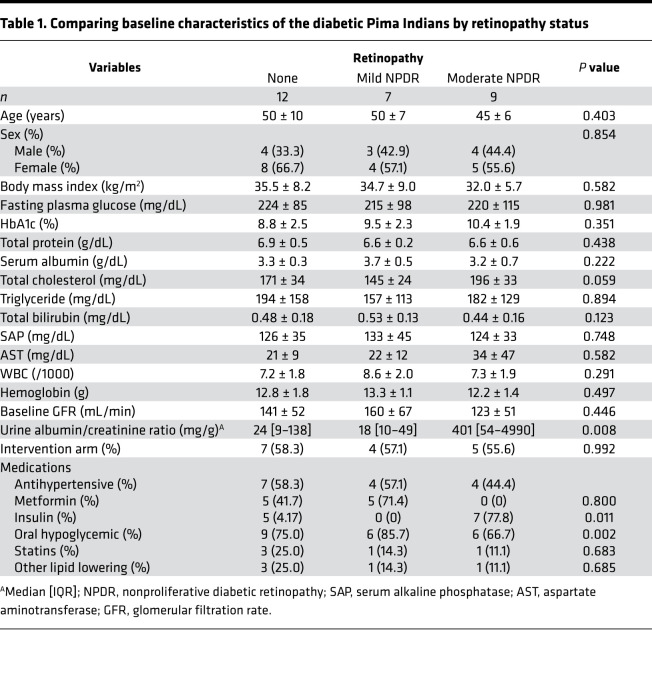
Comparing baseline characteristics of the diabetic Pima Indians by retinopathy status

**Table 2:**
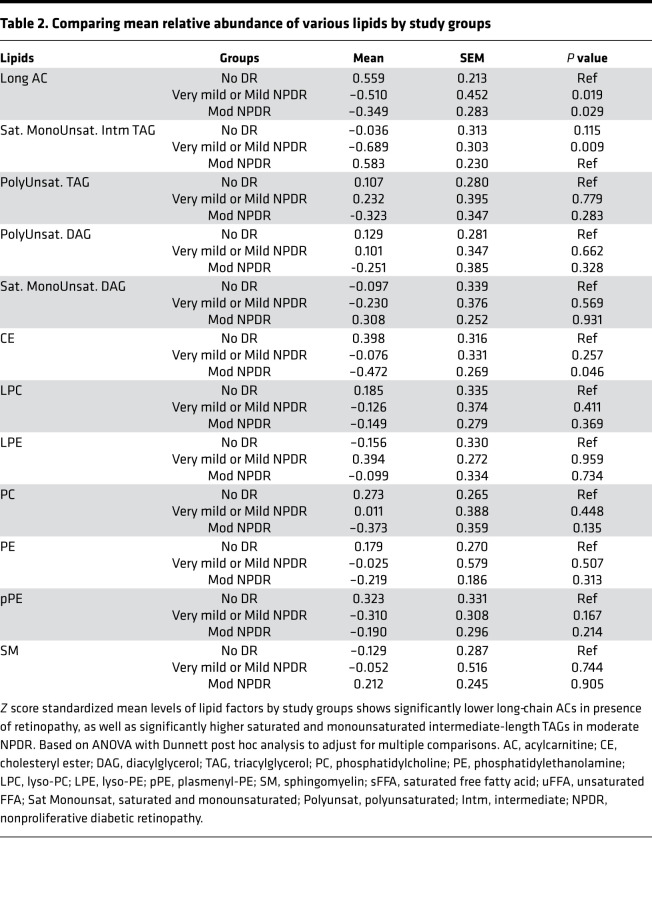
Comparing mean relative abundance of various lipids by study groups

**Table 3:**
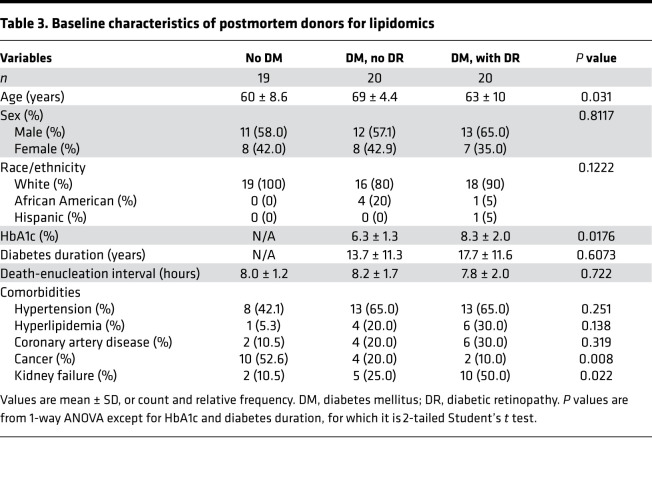
Baseline characteristics of postmortem donors for lipidomics

**Table 4:**
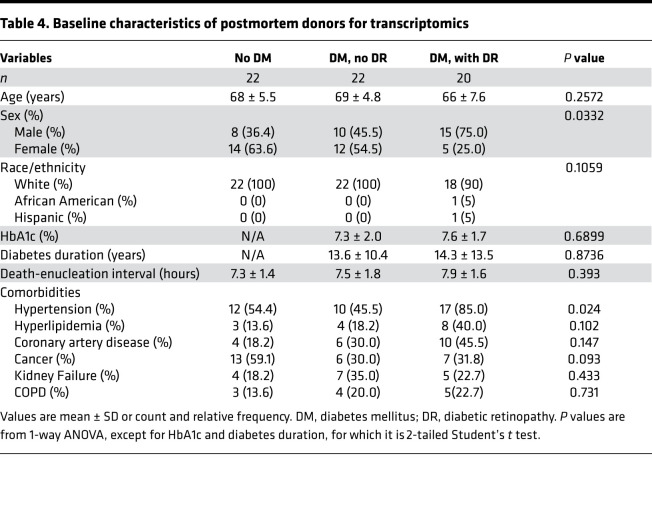
Baseline characteristics of postmortem donors for transcriptomics

**Table 5:**
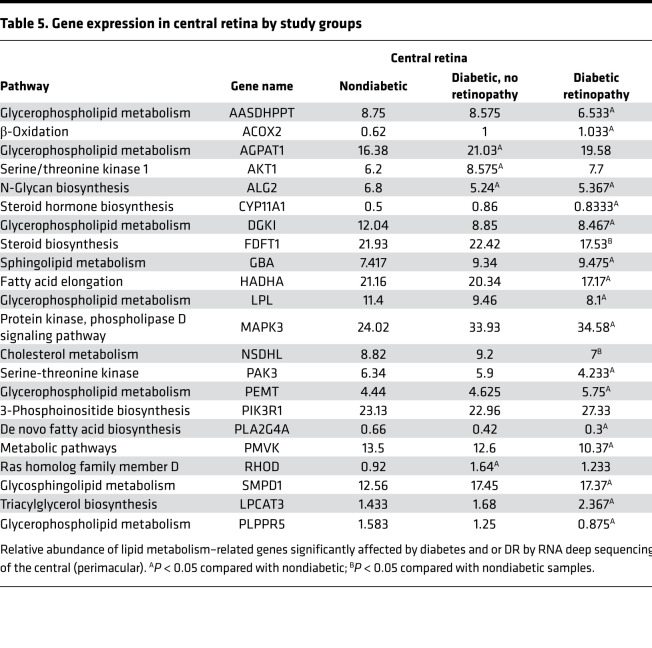
Gene expression in central retina by study groups

**Table 6:**
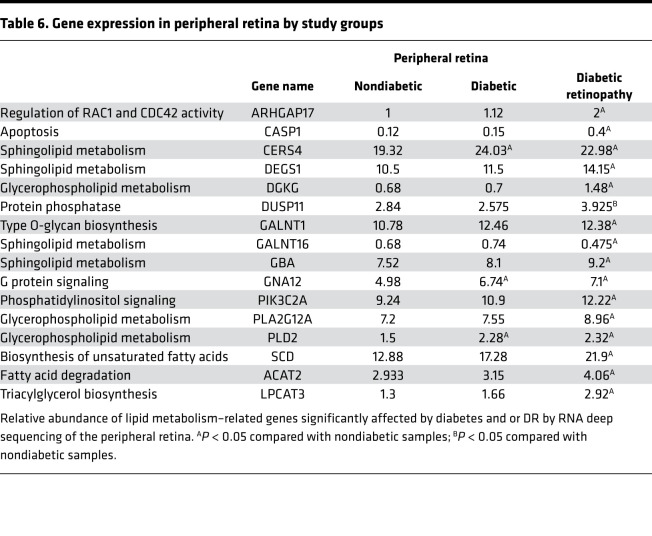
Gene expression in peripheral retina by study groups

## Version 1 08/26/2021

In-Press Preview

## Version 2 10/08/2021

Electronic publication

## References

[1] Duh EJ (2017). Diabetic retinopathy: current understanding, mechanisms, and treatment strategies. JCI Insight.

[2] Yau JW (2012). Global prevalence and major risk factors of diabetic retinopathy. Diabetes Care.

[3] Zhang X (2010). Prevalence of diabetic retinopathy in the United States, 2005-2008. JAMA.

[4] Diabetes Control Complications Trial Research Group (1993). The effect of intensive treatment of diabetes on the development and progression of long-term complications in insulin-dependent diabetes mellitus. N Engl J Med.

[5] Eid S (2019). New insights into the mechanisms of diabetic complications: role of lipids and lipid metabolism. Diabetologia.

[6] Afshinnia F (2019). Increased lipogenesis and impaired β-oxidation predict type 2 diabetic kidney disease progression in American Indians. JCI Insight.

[7] Sas KM (2016). Tissue-specific metabolic reprogramming drives nutrient flux in diabetic complications. JCI Insight.

[8] National Eye Institute. Diabetic Retinopathy. https://www.nei.nih.gov/learn-about-eye-health/eye-conditions-and-diseases/diabetic-retinopathy#:~:text=Diabetic%20retinopathy%20is%20caused%20by,vessels%20all%20over%20the%20body Accessed September 2, 2021

[9] Curovic VR (2020). Circulating metabolites and lipids are associated to diabetic retinopathy in individuals with type 1 diabetes. Diabetes.

[10] Ibrahim AS (2015). A lipidomic screen of hyperglycemia-treated HRECs links 12/15-Lipoxygenase to microvascular dysfunction during diabetic retinopathy via NADPH oxidase. J Lipid Res.

[11] Koehrer P (2014). Erythrocyte phospholipid and polyunsaturated fatty acid composition in diabetic retinopathy. PLoS One.

[12] Sas KM (2018). Shared and distinct lipid-lipid interactions in plasma and affected tissues in a diabetic mouse model. J Lipid Res.

[13] Schwartzman ML (2010). Profile of lipid and protein autacoids in diabetic vitreous correlates with the progression of diabetic retinopathy. Diabetes.

[14] Xuan Q (2020). Rapid lipidomic profiling based on ultra-high performance liquid chromatography-mass spectrometry and its application in diabetic retinopathy. Anal Bioanal Chem.

[15] Tikhonenko M (2010). Remodeling of retinal Fatty acids in an animal model of diabetes: a decrease in long-chain polyunsaturated fatty acids is associated with a decrease in fatty acid elongases Elovl2 and Elovl4. Diabetes.

[16] O’Brien PD (2020). Integrated lipidomic and transcriptomic analyses identify altered nerve triglycerides in mouse models of prediabetes and type 2 diabetes. Dis Model Mech.

[17] Weil EJ (2013). Effect of losartan on prevention and progression of early diabetic nephropathy in American Indians with type 2 diabetes. Diabetes.

[18] Silva PS (2015). Peripheral lesions identified on ultrawide field imaging predict increased risk of diabetic retinopathy progression over 4 years. Ophthalmology.

[19] Shimizu K (1981). Midperipheral fundus involvement in diabetic retinopathy. Ophthalmology.

[20] van der Veen JN (2017). The critical role of phosphatidylcholine and phosphatidylethanolamine metabolism in health and disease. Biochim Biophys Acta Biomembr.

[21] Afshinnia F (2020). Plasma lipidomic profiling identifies a novel complex lipid signature associated with ischemic stroke in chronic kidney disease. J Transl Sci.

[22] Rajagopal R (2018). Retinal de novo lipogenesis coordinates neurotrophic signaling to maintain vision. JCI Insight.

[23] Fox TE (2012). Diabetes diminishes phosphatidic acid in the retina: a putative mediator for reduced mTOR signaling and increased neuronal cell death. Invest Ophthalmol Vis Sci.

[24] Fox TE (2006). Diabetes alters sphingolipid metabolism in the retina: a potential mechanism of cell death in diabetic retinopathy. Diabetes.

[25] Gorusupudi A (2016). Associations of human retinal very long-chain polyunsaturated fatty acids with dietary lipid biomarkers. J Lipid Res.

[26] Enticknap JB (1961). Lipids in cadaver sera after fatal heart attacks. J Clin Pathol.

[27] Montanini L (2013). Human RNA integrity after postmortem retinal tissue recovery. Ophthalmic Genet.

[28] Mozetic V (2019). Statins and/or fibrates for diabetic retinopathy: a systematic review and meta-analysis. Diabetol Metab Syndr.

[29] Fonda SJ (2020). The Indian Health Service Primary Care-Based Teleophthalmology Program for diabetic eye disease surveillance and management. Telemed J E Health.

[30] [No authors listed] (1991). Grading diabetic retinopathy from stereoscopic color fundus photographs--an extension of the modified Airlie House classification. ETDRS report number 10. Early Treatment Diabetic Retinopathy Study Research Group. Ophthalmology.

[31] Ruebsam A (2018). A specific phosphorylation regulates the protective role of αA-crystallin in diabetes. JCI Insight.

[32] Afshinnia F (2016). Lipidomic signature of progression of chronic kidney disease in the chronic renal insufficiency cohort. Kidney Int Rep.

[33] Afshinnia F (2018). Impaired β-oxidation and altered complex lipid fatty acid partitioning with advancing CKD. J Am Soc Nephrol.

[34] Abcouwer SF (2013). Minocycline prevents retinal inflammation and vascular permeability following ischemia-reperfusion injury. J Neuroinflammation.

