# Supplementary material for: Diminished retinal complex lipid synthesis and impaired fatty acid β-oxidation associated with human diabetic retinopathy
Source: JCI Insight. 2021 Oct 8;6(19):e152109. doi: 10.1172/jci.insight.152109 (PMC8525591; doi:10.1172/jci.insight.152109)

Supplement:

Table S1: Identified lipids by adducts, mass, and retention time in positive and negative modes in Pima cohort. The mass accuracy was ±0.001 Da in positive mode and ±0.005 Da in negative mode, with overall mass error of less than 2 parts per million. AC, Acylcarnitines; CE, cholesterol Esters; DAG, diacylglycerol; LPC. lysophosphatidylcholine; LPE, lysophosphatidylethanolamine; MAG, monoacylglycerol; PC, phosphatidylcholine; pPC, plasmenyl-phosphatidylcholine; PE, phosphatidylethanolamine; pPE: plasmenyl-phosphatidylethanolamine; PA, phosphatidic acid; SM, sphingomyelin; TAG, triacylglycerol; FFA, free Fatty Acid; CerP, ceramide-phosphates; PG, phosphatidylglycerol; PI, phosphatidylinositol; x:y, number of carbons:number of double bonds.

**Lipids in Positive Mode**				
**Class**	**Compound name**	**Type of adducts**	**m/z**	**RT/min**
CE	CE 16:0	[M+NH4]	642.6189	10.92
	CE 16:1	[M+NH4]	640.6032	10.65
	CE 17:0	[M+NH4]	656.6345	11.11
	CE 18:0	[M+NH4]	670.6502	11.28
	CE 18:1	[M+NH4]	668.6345	10.89
	CE 18:2	[M+NH4]	666.6189	10.72
	CE 18:3	[M+NH4]	664.6032	10.48
	CE 20:3	[M+NH4]	692.6345	10.74
	CE 20:4	[M+NH4]	690.6189	10.52
	CE 20:5	[M+NH4]	688.6032	10.37
	CE 22:4	[M+NH4]	718.6502	10.82
	CE 22:5	[M+NH4]	716.6345	10.64
	CE 22:6	[M+NH4]	714.6189	10.48
DAG	DAG 26:0	[M+NH4]	502.4469	6.16
	DAG 30:0	[M+NH4]	558.5094	7.38
	DAG 30:1	[M+NH4]	556.4938	6.89
	DAG 31:0	[M+NH4]	572.5251	7.69
	DAG 32:0	[M+NH4]	586.5407	7.90
	DAG 32:1	[M+NH4]	584.5251	7.48
	DAG 32:2	[M+NH4]	582.5094	7.03
	DAG 33:0	[M+NH4]	600.5564	8.12
	DAG 33:1	[M+NH4]	598.5407	7.76
	DAG 34:0	[M+NH4]	614.5720	8.40
	DAG 34:1	[M+NH4]	612.5564	7.96
	DAG 34:2	[M+NH4]	610.5407	7.58
	DAG 34:3	[M+NH4]	608.5251	7.19
	DAG 35:1	[M+NH4]	626.5720	8.20
	DAG 35:2	[M+NH4]	624.5564	7.83
	DAG 36:0	[M+NH4]	642.6033	8.86
	DAG 36:1	[M+NH4]	640.5877	8.47
	DAG 36:2	[M+NH4]	638.5720	8.07

Lipids in Positive Mode				
**Class**	**Compound name**	**Type of adducts**	**m/z**	**RT/min**
	DAG 36:3	[M+NH4]	636.5564	7.68
	DAG 36:4	[M+NH4]	634.5407	7.33
	DAG 36:5	[M+NH4]	632.5251	6.94
	DAG 38:1	[M+NH4]	668.6189	8.93
	DAG 38:2	[M+NH4]	666.6033	8.52
	DAG 38:4	[M+NH4]	662.5720	7.90
	DAG 38:5	[M+NH4]	660.5564	7.61
	DAG 38:6	[M+NH4]	658.5407	7.25
	DAG 38:7	[M+NH4]	656.5251	6.89
	DAG 40:6	[M+NH4]	686.5720	7.68
	DAG 40:7	[M+NH4]	684.5564	7.52
LPC	LPC 14:0	[M+H]	468.3090	1.10
	LPC 14:0	[M+Na]	490.2910	1.16
	LPC 15:0	[M+H]	482.3246	1.36
	LPC 15:0	[M+Na]	504.3066	1.37
	LPC 16:0	[M+H]	496.3403	1.62
	LPC 16:0	[M+Na]	504.3430	2.11
	LPC 16:1	[M+H]	494.3246	1.33
	LPC 16:1	[M+Na]	516.3066	1.25
	LPC 17:0	[M+H]	510.3560	2.05
	LPC 17:0	[M+Na]	532.3379	2.01
	LPC 17:1	[M+H]	508.3403	1.49
	LPC 18:0	[M+H]	524.3716	2.49
	LPC 18:0	[M+Na]	546.3535	2.38
	LPC 18:1	[M+Na]	544.3379	1.69
	LPC 18:2	[M+H]	520.3403	1.60
	LPC 18:2	[M+Na]	542.3223	1.43
	LPC 18:3	[M+H]	518.3246	1.12
	LPC 18:3	[M+Na]	540.3066	1.15
	LPC 19:0	[M+H]	538.3873	2.96
	LPC 20:0	[M+H]	552.4029	3.44
	LPC 20:1	[M+H]	550.3873	2.64
	LPC 20:1	[M+Na]	572.3692	2.62
	LPC 20:2	[M+H]	548.3716	20
	LPC 20:3	[M+H]	546.3560	1.61
	LPC 20:3	[M+Na]	568.3379	1.60
	LPC 20:4	[M+H]	544.3403	1.45
	LPC 20:4	[M+Na]	566.3223	1.34
	LPC 20:5	[M+H]	542.3246	1.08
	LPC 22:4	[M+H]	572.3716	1.86
	LPC 22:4	[M+Na]	594.3535	1.88
	LPC 22:5	[M+H]	570.3560	1.51

Lipids in Positive Mode				
**Class**	**Compound name**	**Type of adducts**	**m/z**	**RT/min**
	LPC 22:6	[M+H]	568.3403	1.30
	LPC 24:0	[M+H]	608.4655	5.30
LPE	LPE 16:0	[M+H]	454.2934	1.69
	LPE 18:0	[M+H]	482.3246	2.48
	LPE 18:0	[M+Na]	504.3066	2.62
	LPE 18:1	[M+H]	480.3090	1.87
	LPE 18:2	[M+H]	478.2934	1.40
	LPE 20:4	[M+H]	502.2934	1.34
	LPE 22:6	[M+H]	526.2933	1.29
MAG	MAG 17:0	[M+Li]	351.3087	3.19
	MAG 18:1	[M+NH4]	374.3270	2.90
PA	PA 34:0	[M+Na]	699.4941	8.37
PC	PC 30:0	[M+Na]	728.5206	6.07
	PC 30:1	[M+Na]	726.5050	5.54
	PC 32:0	[M+Na]	756.5519	6.75
	PC 32:1	[M+Na]	754.5363	6.23
	PC 32:2	[M+Na]	752.5206	5.75
	PC 32:3	[M+H]	728.5230	5.37
	PC 33:2	[M+Na]	766.5363	6.09
	PC 34:0	[M+Na]	784.5832	7.43
	PC 34:2	[M+Na]	780.5519	6.42
	PC 34:3	[M+H]	756.5543	6.78
	PC 34:3	[M+Na]	778.5363	5.93
	PC 34:4	[M+Na]	776.5206	5.68
	PC 36:1	[M+Na]	810.5989	7.57
	PC 36:2	[M+Na]	808.5832	7.16
	PC 36:3	[M+H]	784.5856	7.45
	PC 36:4	[M+H]	782.5700	7.86
	PC 36:4	[M+Na]	804.5519	6.08
	PC 36:5	[M+H]	780.5543	5.60
	PC 36:5	[M+Na]	802.5363	5.73
	PC 36:6	[M+H]	778.5387	5.89
	PC 38:4	[M+H]	810.6013	7.56
	PC 38:4	[M+Na]	832.5832	7.02
	PC 38:5	[M+H]	808.5856	5.09
	PC 38:6	[M+H]	806.5700	6.03
	PC 38:6	[M+Na]	828.5519	5.44
	PC 38:6	[M+Na]	828.5519	6.14
	PC 38:8	[M+H]	802.5387	5.86
	PC 40:10	[M+H]	826.5387	5.62
	PC 40:4	[M+Na]	860.6145	7.55
	PC 40:9	[M+H]	828.5543	6.09

Lipids in Positive Mode				
**Class**	**Compound name**	**Type of adducts**	**m/z**	**RT/min**
	PC 42:10	[M+H]	854.5700	5.83
PE	PE 32:1	[M+H]	690.5074	6.27
	PE 34:0	[M+H]	720.5543	7.38
	PE 34:1	[M+H]	718.5387	6.90
	PE 34:2	[M+H]	716.5230	6.46
	PE 35:2	[M+H]	730.5387	6.79
	PE 36:0	[M+H]	748.5856	7.91
	PE 36:1	[M+H]	746.5700	7.50
	PE 36:2	[M+H]	744.5543	7.01
	PE 36:2	[M+H]	744.5543	7.12
	PE 36:3	[M+H]	742.5387	6.59
	PE 36:4	[M+H]	740.5230	6.40
	PE 36:5	[M+H]	738.5074	5.88
	PE 38:3	[M+H]	770.5700	7.28
	PE 38:4	[M+H]	768.5543	7.04
	PE 38:5	[M+H]	766.5387	6.54
	PE 40:6	[M+H]	792.5543	6.94
pPC	pPC 18:0	[M+Na]	544.3379	1.89
pPE	pPE 34:1	[M+H]	702.5438	7.22
	pPE 34:2	[M+H]	700.5281	6.75
	pPE 34:2	[M+H]	700.5281	6.82
	pPE 36:1	[M+H]	730.5751	7.80
	pPE 36:2	[M+H]	728.5594	7.37
	pPE 36:4	[M+H]	724.5281	6.72
	pPE 36:5	[M+H]	722.5125	6.34
	pPE 38:1	[M+H]	758.6063	8.27
	pPE 38:2	[M+H]	756.5907	7.95
	pPE 38:3	[M+H]	754.5751	7.58
	pPE 38:4	[M+H]	752.5594	7.33
	pPE 38:6	[M+H]	748.5281	6.64
	pPE 40:4	[M+H]	780.5907	7.78
	pPE 40:5	[M+H]	778.5751	7.46
	pPE 40:6	[M+H]	776.5594	7.25
SM	SM 21:0	[M]	523.3876	1.87
	SM 30:1	[M]	647.5128	4.46
	SM 30:1	[M+Na]	669.4948	4.41
	SM 31:1	[M]	661.5284	4.84
	SM 32:0	[M]	677.5597	5.53
	SM 32:0	[M+Na]	699.5417	5.47
	SM 32:1	[M+Na]	697.5261	2.13
	SM 32:1	[M+Na]	697.5261	5.23
	SM 32:2	[M]	673.5284	4.66

Lipids in Positive Mode				
**Class**	**Compound name**	**Type of adducts**	**m/z**	**RT/min**
	SM 33:0	[M]	691.5754	5.86
	SM 33:1	[M]	689.5597	4.41
	SM 33:1	[M+Na]	711.5417	5.60
	SM 33:2	[M]	687.5441	5.05
	SM 34:1	[M]	703.5754	5.89
	SM 34:1	[M+Na]	725.5573	5.94
	SM 34:2	[M+Na]	723.5417	5.37
	SM 35:1	[M]	717.5911	5.67
	SM 35:1	[M]	717.5911	6.26
	SM 35:1	[M+Na]	739.5730	6.27
	SM 35:2	[M]	715.5754	5.66
	SM 35:2	[M+Na]	737.5573	5.78
	SM 36:1	[M+Na]	753.5886	6.67
	SM 36:2	[M+Na]	751.5730	6.14
	SM 37:1	[M]	745.6224	6.97
	SM 37:2	[M]	743.6067	6.56
	SM 38:0	[M]	761.6536	7.55
	SM 38:1	[M]	759.6380	7.10
	SM 38:1	[M+Na]	781.6199	7.32
	SM 38:2	[M]	757.6224	6.69
	SM 38:4	[M]	753.5911	5.87
	SM 39:0	[M]	775.6693	7.85
	SM 39:1	[M+Na]	795.6356	7.66
	SM 39:2	[M]	771.6380	7.15
	SM 40:1	[M+Na]	809.6513	7.92
	SM 40:2	[M]	785.6536	7.05
	SM 40:2	[M+Na]	807.6356	7.44
	SM 41:1	[M+Na]	823.6669	8.21
	SM 41:2	[M+Na]	821.6513	7.76
	SM 41:4	[M]	795.6380	6.86
	SM 41:5	[M]	793.6224	6.32
	SM 42:1	[M+Na]	837.6826	8.49
	SM 42:2	[M]	813.6849	11.70
	SM 42:2	[M+Na]	835.6669	7.93
	SM 42:4	[M]	809.6536	7.00
	SM 42:5	[M]	807.6380	6.61
	SM 43:1	[M]	829.7163	8.77
	SM 43:1	[M+Na]	851.6982	8.78
	SM 43:2	[M]	827.7006	8.31
	SM 43:4	[M]	823.6693	7.34
	SM 44:1	[M]	843.7319	9.05
	SM 44:2	[M]	841.7163	8.49

Lipids in Positive Mode				
**Class**	**Compound name**	**Type of adducts**	**m/z**	**RT/min**
TAG	TAG 40:0	[M+NH4]	712.6451	9.45
	TAG 42:0	[M+NH4]	740.6764	9.77
	TAG 42:1	[M+NH4]	738.6608	9.49
	TAG 44:1	[M+NH4]	766.6921	9.79
	TAG 46:0	[M+NH4]	796.7390	10.31
	TAG 46:1	[M+Na]	799.6787	10.08
	TAG 46:1	[M+NH4]	794.7233	10.04
	TAG 46:2	[M+NH4]	792.7077	9.80
	TAG 48:0	[M+NH4]	824.7702	10.55
	TAG 48:1	[M+NH4]	822.7546	10.29
	TAG 48:2	[M+NH4]	820.7390	10.07
	TAG 48:3	[M+NH4]	818.7233	9.84
	TAG 49:0	[M+NH4]	838.7859	10.66
	TAG 49:1	[M+NH4]	836.7702	10.45
	TAG 49:2	[M+NH4]	834.7546	10.24
	TAG 50:0	[M+NH4]	852.8015	10.79
	TAG 50:1	[M+NH4]	850.7859	10.53
	TAG 50:2	[M+NH4]	848.7702	10.30
	TAG 50:3	[M+NH4]	846.7546	10.11
	TAG 50:3	[M+NH4]	846.7546	9.20
	TAG 50:4	[M+NH4]	844.7390	8.98
	TAG 50:4	[M+NH4]	844.7390	9.91
	TAG 50:5	[M+NH4]	842.7233	9.73
	TAG 51:1	[M+NH4]	864.8015	10.69
	TAG 51:2	[M+NH4]	862.7859	10.51
	TAG 51:3	[M+NH4]	860.7702	10.31
	TAG 51:4	[M+NH4]	858.7546	10.06
	TAG 52:0	[M+Na]	885.7882	11.02
	TAG 52:0	[M+NH4]	880.8328	11.03
	TAG 52:1	[M+Na]	883.7726	10.82
	TAG 52:1	[M+NH4]	878.8172	10.80
	TAG 52:2	[M+NH4]	876.8015	10.54
	TAG 52:3	[M+Na]	879.7413	10.38
	TAG 52:3	[M+NH4]	874.7859	10.36
	TAG 52:4	[M+NH4]	872.7702	10.17
	TAG 52:4	[M+NH4]	872.7702	9.27
	TAG 52:5	[M+NH4]	870.7546	9.99
	TAG 52:6	[M+Na]	873.6944	9.77
	TAG 52:6	[M+NH4]	868.7390	9.79
	TAG 53:0	[M+NH4]	894.8484	11.14
	TAG 53:1	[M+NH4]	892.8328	10.92
	TAG 53:2	[M+NH4]	890.8172	10.73

Lipids in Positive Mode				
**Class**	**Compound name**	**Type of adducts**	**m/z**	**RT/min**
	TAG 53:3	[M+NH4]	888.8015	10.54
	TAG 53:4	[M+NH4]	886.7859	10.34
	TAG 53:5	[M+NH4]	884.7702	10.16
	TAG 54:1	[M+Na]	911.8038	11.02
	TAG 54:1	[M+NH4]	906.8484	11.05
	TAG 54:2	[M+NH4]	904.8328	10.84
	TAG 54:3	[M+NH4]	902.8172	10.63
	TAG 54:4	[M+NH4]	900.8015	10.42
	TAG 54:5	[M+NH4]	898.7859	10.24
	TAG 54:6	[M+Na]	901.7257	10.04
	TAG 54:6	[M+NH4]	896.7702	10.04
	TAG 54:7	[M+Na]	899.7100	9.84
	TAG 54:7	[M+NH4]	894.7546	9.89
	TAG 54:8	[M+NH4]	892.7390	9.67
	TAG 56:0	[M+NH4]	936.8954	11.52
	TAG 56:1	[M+NH4]	934.8797	11.28
	TAG 56:2	[M+NH4]	932.8641	11.06
	TAG 56:3	[M+NH4]	930.8484	10.87
	TAG 56:4	[M+NH4]	928.8328	10.70
	TAG 56:5	[M+NH4]	926.8172	10.54
	TAG 56:6	[M+NH4]	924.8015	10.36
	TAG 56:7	[M+NH4]	922.7859	10.17
	TAG 56:8	[M+NH4]	920.7702	9.99
	TAG 56:9	[M+NH4]	918.7546	9.82
	TAG 58:1	[M+NH4]	962.9110	11.55
	TAG 58:10	[M+NH4]	944.7702	9.91
	TAG 58:3	[M+NH4]	958.8797	11.10
	TAG 58:5	[M+NH4]	954.8484	10.76
	TAG 58:6	[M+NH4]	952.8328	10.60
	TAG 58:7	[M+NH4]	950.8172	10.49
	TAG 58:8	[M+NH4]	948.8015	10.23
	TAG 58:9	[M+NH4]	946.7859	10.10
	TAG 60:12	[M+NH4]	968.7702	10.88
	TAG 62:1	[M+NH4]	1018.9740	12.09
Acylcarnitines	L_Carnitine		162.1	0.57
	AC2:0		204.1	0.73
	AC3:0		218.2	0.87
	AC4:0		232.2	3.22
	AC5:0		246.2	3.86
	AC6:0		260.2	4.50
	AC8:1		286.2	5.30
	AC8:0		288.2	6.12

Lipids in Positive Mode				
**Class**	**Compound name**	**Type of adducts**	**m/z**	**RT/min**
	AC10:1		314.2	6.80
	AC10:0		316.2	7.30
	AC12:0-OH		360.2	7.40
	AC12:1		342.2	7.40
	AC12:0		344.2	8.20
	AC14:0-OH		388.3	8.20
	AC14:1		370.3	8.20
	AC14:0		372.3	9.10
	AC14:2		368.3	7.80
	AC16:0-OH		416.3	9.20
	AC16:1		398.3	9.20
	AC16:0		400.3	9.80
	AC18:2-OH		424.3	9.20
	AC18:2		424.3	9.40
	AC18:1		426.3	10.00
	AC18:0		428.3	10.80
	AC20:4		448.3	9.70
	AC20:3		450.2	10.00
	AC20:2		452.3	10.30
	AC20:1		454.3	11.00
	AC20:0		456.3	12.30

Lipids in Negative Mode				
**Class**	**Compound name**	**Type of Adducts**	**m/z**	**RT/min**
FFA	FFA 16:0		255.2329	2.30
	FFA 18:0		283.2642	3.27
	FFA 18:1		281.2486	2.50
	FFA 18:2		279.2329	1.92
	FFA 20:0		311.2955	4.30
	FFA 20:1		309.2799	3.45
	FFA 20:2		307.2642	2.82
	FFA 22:0		339.3268	5.29
	FFA 22:1		337.3112	4.43
	FFA 22:2		335.2955	3.76
	FFA 22:3		333.2799	3.18
	FFA 24:0		367.3581	6.21
	FFA 24:1		365.3425	5.36
	FFA 24:2		363.3268	4.70
	FFA 24:3		361.3112	4.07
	FFA 20:4		303.2329	1.85

Lipids in Negative Mode				
**Class**	**Compound name**	**Type of Adducts**	**m/z**	**RT/min**
CerP	CerP 34:1	[M-H]	616.4706	5.91
LPE	LPE 16:0	[M-H]	452.2777	1.71
	LPE 18:0	[M-H]	480.3090	2.49
	LPE 18:1	[M-H]	478.2934	1.88
	LPE 18:2	[M-H]	476.2777	1.40
	LPE 20:3	[M-H]	502.2934	1.68
	LPE 20:4	[M-H]	500.2777	1.37
	LPE 22:4	[M-H]	528.3090	1.91
	LPE 22:5	[M-H]	526.2934	1.62
	LPE 22:6	[M-H]	524.2777	1.33
Spingenine	N-(hexadecanoyl)-sphing-4-enine	[M-H]	536.5041	6.85
PA	PA 34:2	[M-H]	671.4652	6.36
PC	PC 30:0	[M-Ac-H]	764.5442	6.12
	PC 30:1	[M-Ac-H]	762.5285	5.59
	PC 30:2	[M-Ac-H]	760.5129	5.06
	PC 32:0	[M-Ac-H]	792.5754	6.81
	PC 32:1	[M-Ac-H]	790.5598	6.26
	PC 32:2	[M-Ac-H]	788.5442	5.77
	PC 32:3	[M-Ac-H]	786.5285	5.32
	PC 33:1	[M-Ac-H]	804.5754	6.60
	PC 33:2	[M-Ac-H]	802.5598	6.13
	PC 34:1	[M-Ac-H]	818.5911	6.85
	PC 34:2	[M-Ac-H]	816.5754	4.54
	PC 34:3	[M-Ac-H]	814.5598	5.94
	PC 34:4	[M-Ac-H]	812.5442	5.71
	PC 35:1	[M-Ac-H]	832.6068	7.24
	PC 35:2	[M-Ac-H]	830.5911	6.75
	PC 35:3	[M-Ac-H]	828.5754	6.26
	PC 36:1	[M-Ac-H]	846.6224	7.54
	PC 36:2	[M-Ac-H]	844.6068	10.73
	PC 36:2	[M-Ac-H]	844.6068	7.04
	PC 36:3	[M-Ac-H]	842.5911	6.56
	PC 36:4	[M-Ac-H]	840.5754	6.23
	PC 36:5	[M-Ac-H]	838.5598	5.81
	PC 36:6	[M-Ac-H]	836.5442	5.45
	PC 37:2	[M-Ac-H]	858.6224	7.42
	PC 37:3	[M-Ac-H]	856.6068	6.91
	PC 37:4	[M-Ac-H]	854.5911	6.68
	PC 37:6	[M-Ac-H]	850.5598	5.91
	PC 38:2	[M-Ac-H]	872.6381	7.71

Lipids in Negative Mode				
**Class**	**Compound name**	**Type of Adducts**	**m/z**	**RT/min**
	PC 38:3	[M-Ac-H]	870.6224	7.30
	PC 38:4	[M-Ac-H]	868.6068	6.92
	PC 38:5	[M-Ac-H]	866.5911	6.55
	PC 38:6	[M-Ac-H]	864.5754	6.19
	PC 38:7	[M-Ac-H]	862.5598	5.65
	PC 39:6	[M-Ac-H]	878.5911	6.58
	PC 40:4	[M-Ac-H]	896.6381	7.53
	PC 40:5	[M-Ac-H]	894.6224	7.21
	PC 40:6	[M-Ac-H]	892.6068	6.83
	PC 40:7	[M-Ac-H]	890.5911	6.31
	PC 40:8	[M-Ac-H]	888.5754	5.90
	PC 42:10	[M-Ac-H]	912.5754	5.78
PE	PE 32:0	[M-H]	690.5074	6.79
	PE 32:1	[M-H]	688.4917	6.28
	PE 33:0	[M-H]	704.5231	7.08
	PE 33:1	[M-H]	702.5074	6.60
	PE 34:1	[M-H]	716.5231	6.90
	PE 34:2	[M-H]	714.5074	6.43
	PE 34:3	[M-H]	712.4917	6.02
	PE 35:1	[M-H]	730.5387	7.14
	PE 35:2	[M-H]	728.5231	6.77
	PE 35:3	[M-H]	726.5074	6.23
	PE 35:4	[M-H]	724.4917	6.09
	PE 36:0	[M-H]	746.5700	7.92
	PE 36:1	[M-H]	744.5543	6.94
	PE 36:1	[M-H]	744.5543	7.49
	PE 36:2	[M-H]	742.5387	7.06
	PE 36:3	[M-H]	740.5231	5.14
	PE 36:3	[M-H]	740.5231	6.58
	PE 36:4	[M-H]	738.5074	6.26
	PE 36:5	[M-H]	736.4917	6.01
	PE 37:4	[M-H]	752.5231	6.72
	PE 38:2	[M-H]	770.5700	7.51
	PE 38:3	[M-H]	768.5543	7.29
	PE 38:4	[M-H]	766.5387	6.96
	PE 38:5	[M-H]	764.5231	6.56
	PE 38:6	[M-H]	762.5074	6.16
	PE 40:4	[M-H]	794.5700	7.47
	PE 40:5	[M-H]	792.5543	7.20
	PE 40:6	[M-H]	790.5387	6.95
	PE 40:7	[M-H]	788.5231	6.44
	PE 40:8	[M-H]	786.5074	6.02

Lipids in Negative Mode				
**Class**	**Compound name**	**Type of Adducts**	**m/z**	**RT/min**
PG	PG 33:0	[M-H]	735.5176	6.04
	PG 36:0	[M-H]	777.5646	6.88
	PG 36:2	[M-H]	773.5333	6.04
PI	PI 32:1	[M-H]	807.5024	5.05
	PI 34:2	[M-H]	833.5180	5.22
	PI 36:2	[M-H]	861.5493	5.87
	PI 36:3	[M-H]	859.5337	5.36
	PI 36:4	[M-H]	857.5180	5.22
	PI 38:3	[M-H]	887.5649	6.04
	PI 38:4	[M-H]	885.5493	5.85
	PI 38:5	[M-H]	883.5337	5.37
pPE	pPE 32:1	[M-H]	672.4968	6.65
	pPE 34:1	[M-H]	700.5281	7.25
	pPE 34:2	[M-H]	698.5124	6.78
	pPE 34:3	[M-H]	696.4968	6.40
	pPE 36:1	[M-H]	728.5594	7.81
	pPE 36:2	[M-H]	726.5438	7.36
	pPE 36:3	[M-H]	724.5281	6.96
	pPE 36:4	[M-H]	722.5124	6.72
	pPE 36:5	[M-H]	720.4968	6.38
	pPE 38:1	[M-H]	756.5907	8.30
	pPE 38:2	[M-H]	754.5751	7.97
	pPE 38:3	[M-H]	752.5594	7.58
	pPE 38:4	[M-H]	750.5438	7.29
	pPE 38:5	[M-H]	748.5281	6.80
	pPE 38:6	[M-H]	746.5124	6.61
	pPE 40:4	[M-H]	778.5751	7.82
	pPE 40:5	[M-H]	776.5594	7.43
	pPE 40:6	[M-H]	774.5438	7.25
	pPE 42:5	[M-H]	804.5907	7.91
	pPE 42:6	[M-H]	802.5751	7.80
PS	PS 36:1	[M-H]	788.5442	6.43
	PS 38:4	[M-H]	810.5285	6.01

Table S2: Identified lipids by adducts, mass, and retention time in positive and negative modes in post-mortem cohort. The mass accuracy was ±0.001 Da in positive mode and ±0.005 Da in negative mode, with overall mass error of less than 2 parts per million. AC, acylcarnitine, CE, cholesterol Esters; BMP, bis(monoacylglycero)phosphate; Cer[AS], ceramide class consisting of α-hydroxy fatty acids and 4-sphingenines; Cer[EOS], omega-acylceramide; Cer[EODS], ceramide class consisting of ester-linked non-hydroxy fatty acids, ω-hydroxy fatty acids and 4-sphinnenines; Cer[NDS], non-hydroxy fatty acids and sphinganine; Cer[NDS], ceramide class consisting of non-hydroxy fatty acids and sphinganines; Cer[NP], ceramide class consisting of non-hydroxy fatty acids and 4-hydroxysphinganines; Cer[NS], non-hydroxy fatty acids and 4-sphingenine ceramide; DAG, diacylglycerol; GlcCer[NS], ceramide class consisting of non-hydroxy fatty acids and 4-sphingenines; FAHFA, Fatty Acid ester of Hydroxyl Fatty Acid; LPC, lysophosphatidylcholine; LPE, lysophosphatidylethanolamine; PC, phosphatidylcholine; pPC, plasmenyl-phosphatidylcholine; PE, phosphatidylethanolamine; pPE: plasmenyl-phosphatidylethanolamine; PS, phosphatidylserine; PA, phosphatidic acid; SM, sphingomyelin; TAG, triacylglycerol; FFA, free Fatty Acid; PG, phosphatidylglycerol; PI, phosphatidylinositol; x:y, number of carbons:number of double bonds.

**Lipids in Positive Mode (n=606)**				
**Class**	**Compound name**	**Type of adducts**	**m/z**	**RT/min**
**CE**	CE16:0	[M+NH4]+	642.6189	11.00
	CE 16:1	[M+NH4]+	640.6033	10.69
	CE 18:0	[M+NH4]+	670.6502	11.38
	CE 18:1	[M+NH4]+	668.6345	10.99
	CE 18:2	[M+NH4]+	666.6189	10.81
	CE 18:3	[M+NH4]+	664.6033	10.55
	CE 18:3	[M+NH4]+	664.6033	9.31
	CE 20:3	[M+NH4]+	692.6345	10.81
	CE 20:4	[M+NH4]+	690.6189	10.63
	CE 20:5	[M+NH4]+	688.6033	10.42
	CE 22:6	[M+NH4]+	714.6189	10.53
**BMP**	BMP 34:1	[M+NH4]+	766.5593	5.67
	BMP 36:2	[M+NH4]+	792.5749	5.72
	BMP 36:3	[M+NH4]+	790.5593	5.37
	BMP 36:4	[M+NH4]+	788.5436	4.91
	BMP 38:4	[M+NH4]+	816.5749	5.59
	BMP 38:5	[M+NH4]+	814.5593	5.32
	BMP 38:6	[M+NH4]+	812.5436	4.86
	BMP 40:6	[M+NH4]+	840.5749	5.37
	BMP 40:7	[M+NH4]+	838.5593	5.24
	BMP 40:8	[M+NH4]+	836.5436	4.78
	BMP 42:10	[M+NH4]+	860.5436	4.76
	BMP 42:8	[M+NH4]+	864.5749	5.41
	BMP 42:9	[M+NH4]+	862.5593	5.01

	BMP 44:10	[M+NH4]+	888.5749	5.27
	BMP 44:11	[M+NH4]+	886.5593	5.07
	BMP 44:12	[M+NH4]+	884.5436	4.68
**Cer[NDS]**	Cer[NDS] 34:0	[M+H]+	540.5350	7.06
	Cer[NDS] 36:0	[M+H]+	568.5663	7.61
	Cer[NDS] 38:0	[M+H]+	596.5976	8.14
	Cer[NDS] 40:0	[M+H]+	624.6289	8.64
	Cer[NDS] 41:0	[M+H]+	638.6446	8.81
	Cer[NDS] 42:0	[M+H]+	652.6602	9.09
**Cer[NS]**	Cer[NS] 32:1	[M+H]+	510.4881	6.22
	Cer[NS] 32:2	[M+H]+	508.4724	5.66
	Cer[NS] 33:1	[M+H]+	524.5037	6.54
	Cer[NS] 33:4	[M+H]+	518.4568	5.44
	Cer[NS] 34:1	[M+H]+	538.5194	6.85
	Cer[NS] 34:2	[M+H]+	536.5037	6.36
	Cer[NS] 35:1	[M+H]+	552.5350	7.19
	Cer[NS] 36:1	[M+H]+	566.5507	7.43
	Cer[NS] 36:2	[M+H]+	564.5350	6.99
	Cer[NS] 37:1	[M+H]+	580.5663	7.74
	Cer[NS] 38:1	[M+H]+	594.5820	7.97
	Cer[NS] 38:2	[M+H]+	592.5663	7.57
	Cer[NS] 39:1	[M+H]+	608.5976	8.28
	Cer[NS] 39:2	[M+H]+	606.5820	7.91
	Cer[NS] 40:1	[M+H]+	622.6133	8.08
	Cer[NS] 40:1	[M+H]+	622.6133	8.47
	Cer[NS] 40:2	[M+H]+	620.5976	8.08
	Cer[NS] 41:1	[M+H]+	636.6289	8.73
	Cer[NS] 41:2	[M+H]+	634.6133	8.29
	Cer[NS] 42:1	[M+H]+	650.6446	8.92
	Cer[NS] 42:2	[M+H]+	648.6289	8.10
	Cer[NS] 42:2	[M+H]+	648.6289	8.51
	Cer[NS] 42:3	[M+H]+	646.6133	8.13
	Cer[NS] 42:4	[M+H]+	644.5976	7.79
	Cer[NS] 43:1	[M+H]+	664.6602	9.09
	Cer[NS] 43:2	[M+H]+	662.6446	8.68
	Cer[NS] 44:1	[M+H]+	678.6759	9.33
	Cer[NS] 44:2	[M+H]+	676.6602	8.92
	Cer[NS] 44:3	[M+H]+	674.6446	8.60
**DAG**	DAG 30:0	[M+NH4]+	558.5098	7.38
	DAG 32:0	[M+NH4]+	586.5411	7.94
	DAG 32:1	[M+NH4]+	584.5254	7.50
	DAG 32:2	[M+NH4]+	582.5098	7.06
	DAG 33:0	[M+NH4]+	600.5567	8.17

	DAG 33:1	[M+NH4]+	598.5411	7.75
	DAG 34:0	[M+NH4]+	614.5724	8.38
	DAG 34:0	[M+NH4]+	614.5724	9.55
	DAG 34:1	[M+NH4]+	612.5567	8.00
	DAG 34:2	[M+NH4]+	610.5411	7.61
	DAG 34:3	[M+NH4]+	608.5254	7.25
	DAG 34:4	[M+NH4]+	606.5098	6.89
	DAG 35:0	[M+NH4]+	628.5880	8.67
	DAG 35:1	[M+NH4]+	626.5724	8.21
	DAG 35:2	[M+NH4]+	624.5567	7.87
	DAG 36:0	[M+NH4]+	642.6037	8.89
	DAG 36:0	[M+NH4]+	642.6037	9.80
	DAG 36:1	[M+NH4]+	640.5880	8.48
	DAG 36:2	[M+NH4]+	638.5724	8.08
	DAG 36:3	[M+NH4]+	636.5567	7.78
	DAG 36:4	[M+NH4]+	634.5411	7.47
	DAG 36:5	[M+NH4]+	632.5254	6.94
	DAG 37:0	[M+NH4]+	656.6193	9.09
	DAG 37:4	[M+NH4]+	648.5567	7.86
	DAG 38:0	[M+NH4]+	670.6350	9.26
	DAG 38:1	[M+NH4]+	668.6193	8.96
	DAG 38:2	[M+NH4]+	666.6037	8.57
	DAG 38:3	[M+NH4]+	664.5880	8.28
	DAG 38:4	[M+NH4]+	662.5724	8.00
	DAG 38:5	[M+NH4]+	660.5567	7.63
	DAG 38:6	[M+NH4]+	658.5411	7.45
	DAG 39:0	[M+NH4]+	684.6506	9.46
	DAG 40:0	[M+NH4]+	698.6662	9.62
	DAG 40:4	[M+NH4]+	690.6037	8.48
	DAG 40:5	[M+NH4]+	688.5880	8.18
	DAG 40:6	[M+NH4]+	686.5724	7.96
	DAG 40:7	[M+NH4]+	684.5567	7.50
	DAG 41:0	[M+NH4]+	712.6819	9.77
	DAG 42:0	[M+NH4]+	726.6975	9.93
	DAG 42:10	[M+NH4]+	706.5411	7.06
	DAG 42:7	[M+NH4]+	712.5880	8.06
	DAG 42:8	[M+NH4]+	710.5724	7.66
	DAG 42:9	[M+NH4]+	708.5567	7.35
	DAG 44:12	[M+NH4]+	730.5411	6.98
**GlcCer[NS]**	GlcCer[NS] 34:1	[M+H]+	700.5727	6.19
	GlcCer[NS] 34:2	[M+H]+	698.5571	5.64
	GlcCer[NS] 35:1	[M+H]+	714.5884	6.50
	GlcCer[NS] 36:1	[M+H]+	728.6040	6.82

	GlcCer[NS] 36:2	[M+H]+	726.5884	6.36
	GlcCer[NS] 40:1	[M+H]+	784.6666	7.93
	GlcCer[NS] 40:2	[M+H]+	782.6510	7.48
	GlcCer[NS] 41:1	[M+H]+	798.6823	8.17
	GlcCer[NS] 41:2	[M+H]+	796.6666	8.02
	GlcCer[NS] 42:1	[M+H]+	812.6979	8.42
	GlcCer[NS] 42:2	[M+H]+	810.6823	7.98
	GlcCer[NS] 42:3	[M+H]+	808.6666	7.52
	GlcCer[NS] 43:1	[M+H]+	826.7136	8.60
	GlcCer[NS] 44:1	[M+H]+	840.7292	8.83
**LPC**	LPC 14:0	[M+H]+	468.3090	1.06
	LPC 15:0	[M+H]+	482.3247	1.19
	LPC 16:0	[M+H]+	496.3403	1.65
	LPC 16:0	[M+H]+	496.3403	3.45
	LPC 16:1	[M+H]+	494.3247	1.19
	LPC 17:0	[M+H]+	510.3560	1.98
	LPC 17:1	[M+H]+	508.3403	1.44
	LPC 18:0	[M+H]+	524.3716	2.40
	LPC 18:1	[M+H]+	522.3560	1.85
	LPC 18:2	[M+H]+	520.3403	1.30
	LPC 18:3	[M+H]+	518.3247	1.08
	LPC 19:0	[M+H]+	538.3873	2.83
	LPC 19:1	[M+H]+	536.3716	2.18
	LPC 20:0	[M+H]+	552.4029	3.26
	LPC 20:1	[M+H]+	550.3873	2.54
	LPC 20:2	[M+H]+	548.3716	1.92
	LPC 20:4	[M+H]+	544.3403	1.28
	LPC 20:5	[M+H]+	542.3247	1.06
	LPC 22:0	[M+H]+	580.4342	4.25
	LPC 22:4	[M+H]+	572.3716	1.77
	LPC 22:5	[M+H]+	570.3560	1.46
	LPC 22:6	[M+H]+	568.3403	1.24
	LPC 24:0	[M+H]+	608.4655	5.12
	LPC 24:1	[M+H]+	606.4499	4.28
	LPC 26:0	[M+H]+	636.4968	5.92
**LPE**	LPE 16:0	[M+H]+	454.2934	1.71
	LPE 18:0	[M+H]+	482.3247	2.49
	LPE 18:1	[M+H]+	480.3090	1.80
	LPE 18:2	[M+H]+	478.2934	1.33
	LPE 20:4	[M+H]+	502.2934	1.31
	LPE 20:4	[M+H]+	502.2934	7.30
	LPE 22:4	[M+H]+	530.3247	1.89
	LPE 22:5	[M+H]+	528.3090	1.53

	LPE 22:6	[M+H]+	526.2934	1.27
**PC**	PC 26:0	[M+H]+	650.4761	4.45
	PC 28:0	[M+H]+	678.5074	5.18
	PC 28:2	[M+H]+	674.4761	4.41
	PC 29:0	[M+H]+	692.5230	5.47
	PC 29:1	[M+H]+	690.5074	5.20
	PC 30:0	[M+H]+	706.5387	5.83
	PC 30:2	[M+H]+	702.5074	5.06
	PC 30:3	[M+H]+	700.4917	4.74
	PC 31:0	[M+H]+	720.5543	6.11
	PC 31:1	[M+H]+	718.5387	5.87
	PC 31:2	[M+H]+	716.5230	5.46
	PC 32:0	[M+H]+	734.5700	5.41
	PC 32:0	[M+H]+	734.5700	6.45
	PC 32:1	[M+H]+	732.5543	5.99
	PC 32:2	[M+H]+	730.5387	5.82
	PC 32:3	[M+H]+	728.5230	5.11
	PC 32:4	[M+H]+	726.5074	4.83
	PC 32:6	[M+H]+	722.4761	4.18
	PC 33:0	[M+H]+	748.5856	6.75
	PC 33:2	[M+H]+	744.5543	5.79
	PC 33:3	[M+H]+	742.5387	5.58
	PC 34:0	[M+H]+	762.6013	7.07
	PC 34:1	[M+H]+	760.5856	4.81
	PC 34:2	[M+H]+	758.5700	6.16
	PC 34:3	[M+H]+	756.5543	4.23
	PC 34:3	[M+H]+	756.5543	5.89
	PC 34:4	[M+H]+	754.5387	5.31
	PC 34:5	[M+H]+	752.5230	5.14
	PC 34:6	[M+H]+	750.5074	4.73
	PC 35:0	[M+H]+	776.6169	7.42
	PC 35:2	[M+H]+	772.5856	6.45
	PC 35:2	[M+H]+	772.5856	7.52
	PC 35:3	[M+H]+	770.5700	6.13
	PC 35:4	[M+H]+	768.5543	5.86
	PC 35:5	[M+H]+	766.5387	5.48
	PC 35:6	[M+H]+	764.5230	6.04
	PC 35:7	[M+H]+	762.5074	5.58
	PC 36:0	[M+H]+	790.6326	7.66
	PC 36:1	[M+H]+	788.6169	7.18
	PC 36:1	[M+H]+	788.6169	9.27
	PC 36:2	[M+H]+	786.6013	6.74
	PC 36:3	[M+H]+	784.5856	6.36

	PC 36:4	[M+H]+	782.5700	4.50
	PC 36:5	[M+H]+	780.5543	4.51
	PC 36:6	[M+H]+	778.5387	5.39
	PC 36:7	[M+H]+	776.5230	4.92
	PC 37:1	[M+H]+	802.6326	7.51
	PC 37:2	[M+H]+	800.6169	7.02
	PC 37:3	[M+H]+	798.6013	6.76
	PC 37:4	[M+H]+	796.5856	6.35
	PC 37:5	[M+H]+	794.5700	6.02
	PC 37:6	[M+H]+	792.5543	5.75
	PC 38:0	[M+H]+	818.6639	8.15
	PC 38:1	[M+H]+	816.6482	7.75
	PC 38:2	[M+H]+	814.6326	7.33
	PC 38:3	[M+H]+	812.6169	7.00
	PC 38:4	[M+H]+	810.6013	5.36
	PC 38:4	[M+H]+	810.6013	6.82
	PC 38:5	[M+H]+	808.5856	6.31
	PC 38:6	[M+H]+	806.5700	6.04
	PC 38:7	[M+H]+	804.5543	5.59
	PC 38:8	[M+H]+	802.5387	5.25
	PC 38:9	[M+H]+	800.5230	4.89
	PC 39:1	[M+H]+	830.6639	7.69
	PC 39:2	[M+H]+	828.6482	7.57
	PC 39:4	[M+H]+	824.6169	7.08
	PC 39:5	[M+H]+	822.6013	6.05
	PC 39:6	[M+H]+	820.5856	6.30
	PC 39:7	[M+H]+	818.5700	5.90
	PC 40:0	[M+H]+	846.6952	8.62
	PC 40:1	[M+H]+	844.6795	8.18
	PC 40:10	[M+H]+	826.5387	5.01
	PC 40:2	[M+H]+	842.6639	7.83
	PC 40:3	[M+H]+	840.6482	7.53
	PC 40:6	[M+H]+	834.6013	6.71
	PC 40:7	[M+H]+	832.5856	6.18
	PC 40:8	[M+H]+	830.5700	5.61
	PC 41:1	[M+H]+	858.6952	8.48
	PC 41:6	[M+H]+	848.6169	7.02
	PC 41:7	[M+H]+	846.6013	6.49
	PC 42:1	[M+H]+	872.7108	8.61
	PC 42:10	[M+H]+	854.5700	5.66
	PC 42:11	[M+H]+	852.5543	5.27
	PC 42:2	[M+H]+	870.6952	8.35
	PC 42:3	[M+H]+	868.6795	7.92

	PC 42:4	[M+H]+	866.6639	7.51
	PC 42:5	[M+H]+	864.6482	7.64
	PC 42:6	[M+H]+	862.6326	6.89
	PC 42:7	[M+H]+	860.6169	6.61
	PC 42:9	[M+H]+	856.5856	5.94
	PC 44:1	[M+H]+	900.7421	9.07
	PC 44:10	[M+H]+	882.6013	6.20
	PC 44:11	[M+H]+	880.5856	5.95
	PC 44:12	[M+H]+	878.5700	5.56
	PC 44:2	[M+H]+	898.7265	8.72
	PC 44:3	[M+H]+	896.7108	8.39
	PC 44:4	[M+H]+	894.6952	8.08
	PC 44:5	[M+H]+	892.6795	7.66
	PC 44:6	[M+H]+	890.6639	7.71
	PC 44:7	[M+H]+	888.6482	7.36
	PC 44:8	[M+H]+	886.6326	6.82
	PC 44:9	[M+H]+	884.6169	6.45
	PC 46:0	[M+H]+	930.7891	9.74
	PC 46:1	[M+H]+	928.7734	9.41
	PC 46:10	[M+H]+	910.6326	6.68
	PC 46:2	[M+H]+	926.7578	9.11
	PC 46:4	[M+H]+	922.7265	8.45
	PC 46:5	[M+H]+	920.7108	8.15
	PC 46:6	[M+H]+	918.6952	7.80
	PC 46:6	[M+H]+	918.6952	8.32
	PC 46:7	[M+H]+	916.6795	7.77
	PC 46:8	[M+H]+	914.6639	7.28
	PC 46:9	[M+H]+	912.6482	7.03
	PC 48:2	[M+H]+	954.7891	9.50
	PC 48:3	[M+H]+	952.7734	9.18
	PC 48:7	[M+H]+	944.7108	8.10
	PC 48:8	[M+H]+	942.6952	7.85
	PC 48:9	[M+H]+	940.6795	7.54
	PC 49:4	[M+H]+	964.7734	9.03
	PC 50:1	[M+H]+	984.8360	10.04
	PC 50:2	[M+H]+	982.8204	9.80
	PC 50:3	[M+H]+	980.8047	9.51
	PC 50:4	[M+H]+	978.7891	9.13
	PC 50:8	[M+H]+	970.7265	8.03
	PC 52:1	[M+H]+	1012.8670	10.35
	PC 52:5	[M+H]+	1004.8050	9.26
	PC 52:8	[M+H]+	998.7578	8.48
**PE**	PE 32:0	[M+H]+	692.5230	6.72

	PE 32:1	[M+H]+	690.5074	6.20
	PE 32:2	[M+H]+	688.4917	5.69
	PE 32:3	[M+H]+	686.4761	5.65
	PE 33:1	[M+H]+	704.5230	6.56
	PE 34:1	[M+H]+	718.5387	6.81
	PE 34:2	[M+H]+	716.5230	6.37
	PE 34:3	[M+H]+	714.5074	5.90
	PE 35:1	[M+H]+	732.5543	7.09
	PE 36:0	[M+H]+	748.5856	7.81
	PE 36:1	[M+H]+	746.5700	7.39
	PE 36:2	[M+H]+	744.5543	6.93
	PE 36:3	[M+H]+	742.5387	6.54
	PE 36:4	[M+H]+	740.5230	6.38
	PE 36:5	[M+H]+	738.5074	5.84
	PE 36:6	[M+H]+	736.4917	5.59
	PE 37:4	[M+H]+	754.5387	6.67
	PE 38:1	[M+H]+	774.6013	7.88
	PE 38:2	[M+H]+	772.5856	7.45
	PE 38:3	[M+H]+	770.5700	7.20
	PE 38:4	[M+H]+	768.5543	6.89
	PE 38:5	[M+H]+	766.5387	6.47
	PE 38:6	[M+H]+	764.5230	6.19
	PE 38:7	[M+H]+	762.5074	5.72
	PE 39:6	[M+H]+	778.5387	6.47
	PE 40:1	[M+H]+	802.6326	8.38
	PE 40:2	[M+H]+	800.6169	7.93
	PE 40:4	[M+H]+	796.5856	7.38
	PE 40:5	[M+H]+	794.5700	7.07
	PE 40:6	[M+H]+	792.5543	6.82
	PE 40:7	[M+H]+	790.5387	6.33
	PE 40:8	[M+H]+	788.5230	5.93
	PE 40:9	[M+H]+	786.5074	5.55
	PE 41:6	[M+H]+	806.5700	7.17
	PE 42:1	[M+H]+	830.6639	8.83
	PE 42:10	[M+H]+	812.5230	5.84
	PE 42:11	[M+H]+	810.5074	5.42
	PE 42:2	[M+H]+	828.6482	8.40
	PE 42:4	[M+H]+	824.6169	7.86
	PE 42:6	[M+H]+	820.5856	7.45
	PE 42:7	[M+H]+	818.5700	6.95
	PE 42:8	[M+H]+	816.5543	6.50
	PE 42:9	[M+H]+	814.5387	6.15
	PE 44:10	[M+H]+	840.5543	6.37

	PE 44:12	[M+H]+	836.5230	5.72
	PE 44:9	[M+H]+	842.5700	6.75
**PG**	PG 34:0	[M+NH4]+	768.5749	6.51
	PG 34:1	[M+NH4]+	766.5593	5.98
	PG 34:2	[M+NH4]+	764.5436	5.59
	PG 36:0	[M+NH4]+	796.6062	7.07
	PG 36:4	[M+NH4]+	788.5436	5.61
**pPC**	pPC 24:0	[M+H]+	606.4499	4.29
	pPC 29:1	[M+H]+	674.5125	5.15
	pPC 30:0	[M+H]+	690.5438	6.23
	pPC 31:0	[M+H]+	704.5594	6.53
	pPC 32:0	[M+H]+	718.5751	6.82
	pPC 32:1	[M+H]+	716.5594	5.97
	pPC 32:2	[M+H]+	714.5438	5.90
	pPC 32:3	[M+H]+	712.5281	5.54
	pPC 32:4	[M+H]+	710.5125	5.21
	pPC 32:5	[M+H]+	708.4968	4.62
	pPC 33:5	[M+H]+	722.5125	6.21
	pPC 34:0	[M+H]+	746.6064	7.00
	pPC 34:1	[M+H]+	744.5907	6.58
	pPC 34:2	[M+H]+	742.5751	6.37
	pPC 34:3	[M+H]+	740.5594	5.94
	pPC 34:4	[M+H]+	738.5438	5.83
	pPC 34:5	[M+H]+	736.5281	5.36
	pPC 35:0	[M+H]+	760.6220	6.20
	pPC 35:1	[M+H]+	758.6064	7.31
	pPC 35:3	[M+H]+	754.5751	6.27
	pPC 35:4	[M+H]+	752.5594	5.10
	pPC 36:0	[M+H]+	774.6377	7.45
	pPC 36:1	[M+H]+	772.6220	7.19
	pPC 36:2	[M+H]+	770.6064	6.89
	pPC 36:3	[M+H]+	768.5907	6.54
	pPC 36:4	[M+H]+	766.5751	6.44
	pPC 36:5	[M+H]+	764.5594	6.06
	pPC 36:6	[M+H]+	762.5438	6.84
	pPC 37:0	[M+H]+	788.6533	6.79
	pPC 37:1	[M+H]+	786.6377	8.61
	pPC 37:3	[M+H]+	782.6064	6.90
	pPC 37:4	[M+H]+	780.5907	6.73
	pPC 37:6	[M+H]+	776.5594	6.92
	pPC 38:2	[M+H]+	798.6377	7.38
	pPC 38:3	[M+H]+	796.6220	6.97
	pPC 38:3	[M+H]+	796.6220	7.20

	pPC 38:4	[M+H]+	794.6064	6.87
	pPC 38:5	[M+H]+	792.5907	6.37
	pPC 38:6	[M+H]+	790.5751	6.36
	pPC 39:4	[M+H]+	808.6220	6.06
	pPC 39:6	[M+H]+	804.5907	6.64
	pPC 40:2	[M+H]+	826.6690	7.47
	pPC 40:3	[M+H]+	824.6533	7.58
	pPC 40:4	[M+H]+	822.6377	7.28
	pPC 40:5	[M+H]+	820.6220	7.00
	pPC 40:6	[M+H]+	818.6064	6.54
	pPC 41:4	[M+H]+	836.6533	6.75
	pPC 42:1	[M+H]+	856.7159	8.64
	pPC 42:2	[M+H]+	854.7003	8.47
	pPC 42:3	[M+H]+	852.6846	8.24
	pPC 42:5	[M+H]+	848.6533	7.37
	pPC 42:6	[M+H]+	846.6377	6.94
	pPC 43:4	[M+H]+	864.6846	7.99
	pPC 44:3	[M+H]+	880.7159	8.64
	pPC 44:4	[M+H]+	878.7003	8.19
	pPC 44:5	[M+H]+	876.6846	8.16
	pPC 44:6	[M+H]+	874.6690	7.59
	pPC 46:4	[M+H]+	906.7316	8.52
**pPE**	pPE 32:0	[M+H]+	676.5281	7.06
	pPE 32:1	[M+H]+	674.5125	6.56
	pPE 34:0	[M+H]+	704.5594	7.59
	pPE 34:1	[M+H]+	702.5438	7.10
	pPE 34:2	[M+H]+	700.5281	6.73
	pPE 35:1	[M+H]+	716.5594	7.42
	pPE 36:0	[M+H]+	732.5907	8.13
	pPE 36:1	[M+H]+	730.5751	7.65
	pPE 36:2	[M+H]+	728.5594	7.30
	pPE 36:3	[M+H]+	726.5438	6.86
	pPE 36:4	[M+H]+	724.5281	6.67
	pPE 36:5	[M+H]+	722.5125	6.31
	pPE 37:4	[M+H]+	738.5438	6.95
	pPE 38:2	[M+H]+	756.5907	7.84
	pPE 38:3	[M+H]+	754.5751	7.43
	pPE 38:4	[M+H]+	752.5594	7.22
	pPE 38:5	[M+H]+	750.5438	6.70
	pPE 38:6	[M+H]+	748.5281	6.55
	pPE 39:5	[M+H]+	764.5594	6.98
	pPE 40:4	[M+H]+	780.5907	7.65
	pPE 40:5	[M+H]+	778.5751	7.36

	pPE 40:6	[M+H]+	776.5594	7.17
	pPE 42:4	[M+H]+	808.6220	8.02
	pPE 42:5	[M+H]+	806.6064	7.86
	pPE 42:6	[M+H]+	804.5907	7.71
**PS**	PS 34:0	[M+H]+	764.5441	6.53
	PS 34:1	[M+H]+	762.5285	5.94
	PS 34:2	[M+H]+	760.5128	5.53
	PS 35:1	[M+H]+	776.5441	6.25
	PS 36:1	[M+H]+	790.5598	6.59
	PS 36:2	[M+H]+	788.5441	6.17
	PS 36:3	[M+H]+	786.5285	5.82
	PS 36:4	[M+H]+	784.5128	5.47
	PS 38:1	[M+H]+	818.5911	7.15
	PS 38:2	[M+H]+	816.5755	6.79
	PS 38:3	[M+H]+	814.5598	6.39
	PS 38:4	[M+H]+	812.5441	6.12
	PS 38:5	[M+H]+	810.5285	5.63
	PS 38:6	[M+H]+	808.5128	5.37
	PS 39:6	[M+H]+	822.5285	5.72
	PS 40:1	[M+H]+	846.6224	7.83
	PS 40:2	[M+H]+	844.6068	7.29
	PS 40:4	[M+H]+	840.5755	6.52
	PS 40:5	[M+H]+	838.5598	6.38
	PS 40:6	[M+H]+	836.5441	4.01
	PS 40:7	[M+H]+	834.5285	5.46
	PS 40:8	[M+H]+	832.5128	5.04
	PS 42:1	[M+H]+	874.6537	8.29
	PS 42:10	[M+H]+	856.5128	4.97
	PS 42:11	[M+H]+	854.4972	4.57
	PS 42:2	[M+H]+	872.6381	7.83
	PS 42:5	[M+H]+	866.5911	6.79
	PS 42:6	[M+H]+	864.5755	6.72
	PS 42:7	[M+H]+	862.5598	6.03
	PS 42:8	[M+H]+	860.5441	5.63
	PS 42:9	[M+H]+	858.5285	5.25
	PS 44:10	[M+H]+	884.5441	5.45
	PS 44:11	[M+H]+	882.5285	5.15
	PS 44:12	[M+H]+	880.5128	4.86
	PS 44:7	[M+H]+	890.5911	6.79
	PS 44:9	[M+H]+	886.5598	5.85
	PS 46:10	[M+H]+	912.5755	5.95
	PS 46:9	[M+H]+	914.5911	6.36
**SM**	SM 30:1	[M+H]+	647.5128	4.36

	SM 30:2	[M+H]+	645.4972	3.70
	SM 31:1	[M+H]+	661.5285	4.73
	SM 32:0	[M+H]+	677.5598	5.41
	SM 32:1	[M+H]+	675.5441	5.07
	SM 32:2	[M+H]+	673.5285	4.50
	SM 33:0	[M+H]+	691.5754	5.75
	SM 33:2	[M+H]+	687.5441	4.95
	SM 34:1	[M+H]+	703.5754	5.74
	SM 34:2	[M+H]+	701.5598	5.24
	SM 34:3	[M+H]+	699.5441	4.75
	SM 35:2	[M+H]+	715.5754	5.67
	SM 36:1	[M+H]+	731.6067	6.43
	SM 36:1	[M+H]+	731.6067	9.64
	SM 36:2	[M+H]+	729.5911	5.97
	SM 36:3	[M+H]+	727.5754	5.54
	SM 36:4	[M+H]+	725.5598	5.01
	SM 37:1	[M+H]+	745.6223	6.79
	SM 37:2	[M+H]+	743.6067	6.35
	SM 37:4	[M+H]+	739.5754	6.52
	SM 38:0	[M+H]+	761.6536	7.35
	SM 38:1	[M+H]+	759.6380	7.03
	SM 38:1	[M+H]+	759.6380	9.88
	SM 38:3	[M+H]+	755.6067	6.23
	SM 38:4	[M+H]+	753.5911	5.63
	SM 39:1	[M+H]+	773.6536	7.36
	SM 40:1	[M+H]+	787.6693	7.61
	SM 40:2	[M+H]+	785.6536	7.15
	SM 40:3	[M+H]+	783.6380	6.72
	SM 40:4	[M+H]+	781.6223	6.32
	SM 40:8	[M+H]+	773.5598	7.16
	SM 41:1	[M+H]+	801.6849	7.87
	SM 42:1	[M+H]+	815.7006	8.13
	SM 42:2	[M+H]+	813.6849	7.65
	SM 42:3	[M+H]+	811.6693	7.27
	SM 42:4	[M+H]+	809.6536	6.89
	SM 42:5	[M+H]+	807.6380	6.54
	SM 42:6	[M+H]+	805.6223	6.06
	SM 42:7	[M+H]+	803.6067	7.47
	SM 43:1	[M+H]+	829.7162	8.35
	SM 43:2	[M+H]+	827.7006	7.90
	SM 43:3	[M+H]+	825.6849	7.45
	SM 43:6	[M+H]+	819.6380	7.72
	SM 44:1	[M+H]+	843.7319	8.60

	SM 44:2	[M+H]+	841.7162	8.17
	SM 44:3	[M+H]+	839.7006	7.80
	SM 44:4	[M+H]+	837.6849	7.40
	SM 44:5	[M+H]+	835.6693	7.03
**TAG**	TAG 36:0	[M+NH4]+	656.5829	8.68
	TAG 38:0	[M+NH4]+	684.6142	9.12
	TAG 40:0	[M+NH4]+	712.6455	9.49
	TAG 40:1	[M+NH4]+	710.6299	9.16
	TAG 41:0	[M+NH4]+	726.6612	9.62
	TAG 42:0	[M+NH4]+	740.6768	9.80
	TAG 42:1	[M+NH4]+	738.6612	9.51
	TAG 42:2	[M+NH4]+	736.6455	9.23
	TAG 42:3	[M+NH4]+	734.6299	8.97
	TAG 43:0	[M+NH4]+	754.6925	9.89
	TAG 44:0	[M+NH4]+	768.7081	10.07
	TAG 44:1	[M+NH4]+	766.6925	9.83
	TAG 44:2	[M+NH4]+	764.6768	9.60
	TAG 45:0	[M+NH4]+	782.7238	10.18
	TAG 45:1	[M+NH4]+	780.7081	9.96
	TAG 45:2	[M+NH4]+	778.6925	9.74
	TAG 46:0	[M+NH4]+	796.7394	10.33
	TAG 46:1	[M+NH4]+	794.7238	10.10
	TAG 46:2	[M+NH4]+	792.7081	9.88
	TAG 46:3	[M+NH4]+	790.6925	9.61
	TAG 47:0	[M+NH4]+	810.7551	10.43
	TAG 47:1	[M+NH4]+	808.7394	10.23
	TAG 47:2	[M+NH4]+	806.7238	10.04
	TAG 47:3	[M+NH4]+	804.7081	9.83
	TAG 48:0	[M+NH4]+	824.7707	10.58
	TAG 48:1	[M+NH4]+	822.7551	10.35
	TAG 48:2	[M+NH4]+	820.7394	10.15
	TAG 48:3	[M+NH4]+	818.7238	9.96
	TAG 48:4	[M+NH4]+	816.7081	9.69
	TAG 48:5	[M+NH4]+	814.6925	9.50
	TAG 49:0	[M+NH4]+	838.7864	10.68
	TAG 49:1	[M+NH4]+	836.7707	10.45
	TAG 49:2	[M+NH4]+	834.7551	10.30
	TAG 49:3	[M+NH4]+	832.7394	10.07
	TAG 50:0	[M+NH4]+	852.8020	10.86
	TAG 50:1	[M+Na]+	855.7418	9.25
	TAG 50:1	[M+NH4]+	850.7864	10.59
	TAG 50:2	[M+NH4]+	848.7707	10.37
	TAG 50:3	[M+NH4]+	846.7551	10.19

	TAG 50:4	[M+NH4]+	844.7394	9.99
	TAG 50:5	[M+NH4]+	842.7238	9.78
	TAG 50:6	[M+NH4]+	840.7081	9.58
	TAG 51:0	[M+NH4]+	866.8177	10.92
	TAG 51:1	[M+NH4]+	864.8020	10.71
	TAG 51:2	[M+NH4]+	862.7864	10.52
	TAG 51:3	[M+NH4]+	860.7707	10.33
	TAG 51:4	[M+NH4]+	858.7551	10.14
	TAG 51:5	[M+NH4]+	856.7394	9.91
	TAG 52:0	[M+Na]+	885.7887	11.02
	TAG 52:0	[M+NH4]+	880.8333	11.02
	TAG 52:1	[M+Na]+	883.7731	10.49
	TAG 52:1	[M+NH4]+	878.8177	10.87
	TAG 52:2	[M+NH4]+	876.8020	10.62
	TAG 52:3	[M+Na]+	879.7418	10.40
	TAG 52:3	[M+NH4]+	874.7864	10.42
	TAG 52:4	[M+NH4]+	872.7707	10.27
	TAG 52:5	[M+Na]+	875.7105	10.05
	TAG 52:5	[M+NH4]+	870.7551	10.08
	TAG 52:6	[M+NH4]+	868.7394	9.90
	TAG 53:0	[M+NH4]+	894.8490	11.18
	TAG 53:1	[M+NH4]+	892.8333	10.96
	TAG 53:2	[M+NH4]+	890.8177	10.75
	TAG 53:3	[M+NH4]+	888.8020	10.56
	TAG 53:4	[M+NH4]+	886.7864	10.38
	TAG 53:5	[M+NH4]+	884.7707	10.18
	TAG 54:0	[M+NH4]+	908.8646	11.30
	TAG 54:0	[M+NH4]+	908.8646	12.28
	TAG 54:1	[M+NH4]+	906.8490	11.11
	TAG 54:2	[M+NH4]+	904.8333	10.88
	TAG 54:3	[M+NH4]+	902.8177	10.68
	TAG 54:4	[M+NH4]+	900.8020	10.53
	TAG 54:5	[M+NH4]+	898.7864	10.37
	TAG 54:6	[M+NH4]+	896.7707	10.17
	TAG 54:7	[M+NH4]+	894.7551	10.07
	TAG 54:8	[M+NH4]+	892.7394	9.70
	TAG 54:9	[M+NH4]+	890.7238	9.73
	TAG 56:0	[M+NH4]+	936.8959	11.52
	TAG 56:1	[M+NH4]+	934.8803	11.27
	TAG 56:2	[M+NH4]+	932.8646	11.08
	TAG 56:3	[M+NH4]+	930.8490	10.90
	TAG 56:4	[M+NH4]+	928.8333	10.74
	TAG 56:5	[M+NH4]+	926.8177	10.62

	TAG 56:6	[M+NH4]+	924.8020	10.41
	TAG 56:7	[M+NH4]+	922.7864	10.26
	TAG 56:8	[M+NH4]+	920.7707	10.09
	TAG 58:0	[M+NH4]+	964.9272	11.81
	TAG 58:1	[M+NH4]+	962.9116	11.58
	TAG 58:10	[M+NH4]+	944.7707	10.04
	TAG 58:3	[M+NH4]+	958.8803	11.14
	TAG 58:4	[M+NH4]+	956.8646	11.06
	TAG 58:5	[M+NH4]+	954.8490	10.81
	TAG 58:6	[M+NH4]+	952.8333	10.74
	TAG 58:8	[M+NH4]+	948.8020	10.36
	TAG 58:9	[M+NH4]+	946.7864	10.16
	TAG 60:10	[M+NH4]+	972.8020	10.30
	TAG 60:11	[M+NH4]+	970.7864	10.11
	TAG 60:12	[M+NH4]+	968.7707	9.96
	TAG 60:7	[M+NH4]+	978.8490	10.83
	TAG 60:9	[M+NH4]+	974.8177	10.41
	TAG 62:1	[M+NH4]+	1018.9740	12.17
	TAG 62:12	[M+NH4]+	996.8020	10.31
	TAG 62:13	[M+NH4]+	994.7864	9.99
	TAG 62:14	[M+NH4]+	992.7707	9.88
	TAG 64:15	[M+NH4]+	1018.7860	9.93
	TAG 64:16	[M+NH4]+	1016.7710	9.81

**Lipids in Negative Mode (n=487)**				
**Class**	Compound name	Type of adducts	m/z	RT/min
**FFA**	FFA(16:0)	[M-H]-	255.2329	2.30
	FFA(18:0)	[M-H]-	283.2642	3.27
	FFA(18:1)	[M-H]-	281.2486	2.50
	FFA(18:2)	[M-H]-	279.2329	1.92
	FFA(20:0)	[M-H]-	311.2955	4.30
	FFA(20:1)	[M-H]-	309.2799	3.45
	FFA(20:2)	[M-H]-	307.2642	2.82
	FFA(20:4)	[M-H]-	303.2329	1.85
	FFA(22:0)	[M-H]-	339.3268	5.29
	FFA(22:1)	[M-H]-	337.3112	4.43
	FFA(22:2)	[M-H]-	335.2955	3.76
	FFA(22:3)	[M-H]-	333.2799	3.18
	FFA(24:0)	[M-H]-	367.3581	6.21
	FFA(24:1)	[M-H]-	365.3425	5.36
	FFA(24:2)	[M-H]-	363.3268	4.70
	FFA(24:3)	[M-H]-	361.3112	4.07
**Cer[AS]**	Cer[AS] 35:1	[M+Hac-H]-	626.5360	6.55

	Cer[AS] 36:1	[M+Hac-H]-	640.5516	7.22
	Cer[AS] 37:1	[M+Hac-H]-	654.5673	7.41
	Cer[AS] 38:2	[M+Hac-H]-	666.5673	7.59
	Cer[AS] 39:2	[M+Hac-H]-	680.5829	7.60
**Cer[EODS]**	Cer[EODS] 56:0	[M-H]-	876.8384	8.33
	Cer[EODS] 58:0	[M-H]-	904.8697	8.81
	Cer[EODS] 58:1	[M+Hac-H]-	962.8752	10.58
	Cer[EODS] 60:0	[M-H]-	932.9010	8.94
	Cer[EODS] 60:1	[M-H]-	930.8854	8.50
**Cer[EOS]**	Cer[EOS] 60:1	[M-H]-	930.8854	8.51
**Cer[NDS]**	Cer[NDS] 34:0	[M+Hac-H]-	598.5411	7.03
	Cer[NDS] 34:0	[M-H]-	538.5199	7.07
	Cer[NDS] 36:0	[M+Hac-H]-	626.5724	7.63
	Cer[NDS] 36:0	[M-H]-	566.5512	7.63
	Cer[NDS] 38:0	[M+Hac-H]-	654.6037	8.12
	Cer[NDS] 38:0	[M-H]-	594.5825	8.14
	Cer[NDS] 40:0	[M+Hac-H]-	682.6350	8.60
	Cer[NDS] 40:0	[M-H]-	622.6138	8.64
	Cer[NDS] 42:0	[M-H]-	650.6451	9.10
	Cer[NDS] 42:1	[M-H]-	648.6295	8.65
**Cer[NP]**	Cer[NP] 35:0	[M+Hac-H]-	628.5516	7.05
	Cer[NP] 35:1	[M+Hac-H]-	626.5360	6.58
	Cer[NP] 36:0	[M+Hac-H]-	642.5673	7.17
	Cer[NP] 38:0	[M+Hac-H]-	670.5986	7.73
	Cer[NP] 40:0	[M+Hac-H]-	698.6299	8.22
	Cer[NP] 42:0	[M+Hac-H]-	726.6612	8.68
	Cer[NP] 42:1	[M+Hac-H]-	724.6455	8.25
	Cer[NP] 42:2	[M+Hac-H]-	722.6299	7.88
**Cer[NS]**	Cer[NS] 30:1	[M-H]-	480.4417	5.50
	Cer[NS] 31:1	[M+Hac-H]-	554.4784	5.85
	Cer[NS] 32:1	[M+Hac-H]-	568.4941	6.21
	Cer[NS] 32:1	[M-H]-	508.4730	6.22
	Cer[NS] 32:2	[M+Hac-H]-	566.4784	5.66
	Cer[NS] 33:1	[M+Hac-H]-	582.5098	6.54
	Cer[NS] 33:1	[M-H]-	522.4886	6.55
	Cer[NS] 34:1	[M+Hac-H]-	596.5254	6.81
	Cer[NS] 34:1	[M-H]-	536.5043	6.85
	Cer[NS] 34:2	[M+Hac-H]-	594.5098	6.33
	Cer[NS] 34:2	[M-H]-	534.4886	6.35
	Cer[NS] 35:2	[M+Hac-H]-	608.5254	6.71
	Cer[NS] 36:1	[M+Hac-H]-	624.5567	7.40
	Cer[NS] 36:1	[M-H]-	564.5356	7.42
	Cer[NS] 36:2	[M+Hac-H]-	622.5411	6.98

	Cer[NS] 36:2	[M+Hac-H]-	622.5411	7.46
	Cer[NS] 36:2	[M-H]-	562.5199	7.01
	Cer[NS] 37:1	[M+Hac-H]-	638.5724	7.70
	Cer[NS] 37:1	[M-H]-	578.5512	7.74
	Cer[NS] 37:2	[M+Hac-H]-	636.5567	7.32
	Cer[NS] 38:1	[M+Hac-H]-	652.5880	7.96
	Cer[NS] 38:1	[M-H]-	592.5669	7.97
	Cer[NS] 38:2	[M+Hac-H]-	650.5724	7.59
	Cer[NS] 38:2	[M-H]-	590.5512	7.58
	Cer[NS] 38:3	[M+Hac-H]-	648.5567	7.04
	Cer[NS] 39:1	[M+Hac-H]-	666.6037	8.25
	Cer[NS] 39:1	[M-H]-	606.5825	8.26
	Cer[NS] 39:2	[M+Hac-H]-	664.5880	7.88
	Cer[NS] 40:1	[M+Hac-H]-	680.6193	8.45
	Cer[NS] 40:1	[M-H]-	620.5982	8.45
	Cer[NS] 40:2	[M+Hac-H]-	678.6037	8.10
	Cer[NS] 40:2	[M-H]-	618.5825	8.11
	Cer[NS] 40:3	[M-H]-	616.5669	7.62
	Cer[NS] 41:1	[M+Hac-H]-	694.6350	8.68
	Cer[NS] 41:1	[M-H]-	634.6138	8.73
	Cer[NS] 41:2	[M+Hac-H]-	692.6193	8.24
	Cer[NS] 41:2	[M-H]-	632.5982	8.28
	Cer[NS] 42:1	[M+Hac-H]-	708.6506	8.88
	Cer[NS] 42:1	[M-H]-	648.6295	8.90
	Cer[NS] 42:2	[M+Hac-H]-	706.6350	8.49
	Cer[NS] 42:2	[M-H]-	646.6138	8.48
	Cer[NS] 42:3	[M+Hac-H]-	704.6193	8.11
	Cer[NS] 42:3	[M-H]-	644.5982	8.13
	Cer[NS] 43:1	[M+Hac-H]-	722.6663	9.03
	Cer[NS] 43:1	[M-H]-	662.6451	9.06
**FAHFA**	FAHFA 30:2	[M-H]-	477.3949	1.51
	FAHFA 31:0	[M-H]-	495.4419	1.73
	FAHFA 32:1	[M-H]-	507.4419	1.67
	FAHFA 33:0	[M-H]-	523.4732	2.29
	FAHFA 34:0	[M-H]-	537.4888	2.43
	FAHFA 34:2	[M-H]-	533.4575	1.88
	FAHFA 34:2	[M-H]-	533.4575	2.42
	FAHFA 34:3	[M-H]-	531.4419	0.99
	FAHFA 34:4	[M-H]-	529.4262	1.72
	FAHFA 34:5	[M-H]-	527.4106	1.51
	FAHFA 35:0	[M-H]-	551.5045	2.56
	FAHFA 35:2	[M-H]-	547.4732	2.58
	FAHFA 36:0	[M-H]-	565.5201	3.12

	FAHFA 36:0	[M-H]-	565.5201	7.77
	FAHFA 36:1	[M-H]-	563.5045	2.54
	FAHFA 36:2	[M-H]-	561.4888	2.49
	FAHFA 36:3	[M-H]-	559.4732	1.48
	FAHFA 36:3	[M-H]-	559.4732	2.09
	FAHFA 36:3	[M-H]-	559.4732	2.49
	FAHFA 36:4	[M-H]-	557.4575	2.22
	FAHFA 37:3	[M-H]-	573.4888	1.78
	FAHFA 37:3	[M-H]-	573.4888	2.55
	FAHFA 38:0	[M-H]-	593.5514	3.43
	FAHFA 38:2	[M-H]-	589.5201	2.73
	FAHFA 38:3	[M-H]-	587.5045	10.50
	FAHFA 38:3	[M-H]-	587.5045	9.69
	FAHFA 38:4	[M-H]-	585.4888	2.50
	FAHFA 38:5	[M-H]-	583.4732	2.01
	FAHFA 38:6	[M-H]-	581.4575	1.89
	FAHFA 40:3	[M-H]-	615.5358	10.09
	FAHFA 40:3	[M-H]-	615.5358	10.78
	FAHFA 40:4	[M-H]-	613.5201	2.52
	FAHFA 40:5	[M-H]-	611.5045	1.97
	FAHFA 40:6	[M-H]-	609.4888	2.07
	FAHFA 40:7	[M-H]-	607.4732	1.88
	FAHFA 40:8	[M-H]-	605.4575	1.96
	FAHFA 42:2	[M-H]-	645.5827	4.28
	FAHFA 42:3	[M-H]-	643.5671	4.30
	FAHFA 42:7	[M-H]-	635.5045	1.86
	FAHFA 42:8	[M-H]-	633.4888	2.06
	FAHFA 42:9	[M-H]-	631.4732	1.82
	FAHFA 44:11	[M-H]-	655.4732	1.71
	FAHFA 44:9	[M-H]-	659.5045	1.76
**GlcCer[NS]**	GlcCer[NS] 34:1	[M+Hac-H]-	758.5782	6.15
	GlcCer[NS] 36:1	[M+Hac-H]-	786.6095	6.78
	GlcCer[NS] 40:1	[M+Hac-H]-	842.6721	7.87
	GlcCer[NS] 42:1	[M+Hac-H]-	870.7034	8.36
	GlcCer[NS] 42:2	[M+Hac-H]-	868.6878	7.87
**LPC**	LPC 14:0	[M+Hac-H]-	526.3145	1.04
	LPC 15:0	[M+Hac-H]-	540.3302	1.30
	LPC 16:0	[M+Hac-H]-	554.3458	1.58
	LPC 16:1	[M+Hac-H]-	552.3302	1.16
	LPC 17:1	[M+Hac-H]-	566.3458	1.44
	LPC 18:0	[M+Hac-H]-	582.3771	2.38
	LPC 18:1	[M+Hac-H]-	580.3614	1.67
	LPC 18:2	[M+Hac-H]-	578.3458	1.31

	LPC 18:3	[M+Hac-H]-	576.3302	1.05
	LPC 19:0	[M+Hac-H]-	596.3927	2.85
	LPC 20:0	[M+Hac-H]-	610.4084	3.32
	LPC 20:1	[M+Hac-H]-	608.3927	2.52
	LPC 20:2	[M+Hac-H]-	606.3771	1.96
	LPC 20:3	[M+Hac-H]-	604.3614	1.51
	LPC 20:4	[M+Hac-H]-	602.3458	1.27
	LPC 20:5	[M+Hac-H]-	600.3302	1.01
	LPC 22:4	[M+Hac-H]-	630.3771	1.72
	LPC 22:5	[M+Hac-H]-	628.3614	1.41
	LPC 22:6	[M+Hac-H]-	626.3458	1.19
	LPC 24:0	[M+Hac-H]-	666.4710	5.13
**LPE**	LPE 16:0	[M-H]-	452.2777	1.68
	LPE 18:0	[M-H]-	480.3090	2.50
	LPE 18:1	[M-H]-	478.2934	1.80
	LPE 18:2	[M-H]-	476.2777	1.37
	LPE 20:1	[M-H]-	506.3247	2.68
	LPE 20:3	[M-H]-	502.2934	1.65
	LPE 20:4	[M-H]-	500.2777	1.33
	LPE 20:5	[M-H]-	498.2621	1.03
	LPE 22:4	[M-H]-	528.3090	1.83
	LPE 22:5	[M-H]-	526.2934	1.61
	LPE 22:6	[M-H]-	524.2777	1.26
**PA**	PA 34:1	[M-H]-	673.4808	6.64
	PA 34:2	[M-H]-	671.4652	6.22
	PA 36:1	[M-H]-	701.5121	6.41
	PA 38:3	[M-H]-	725.5121	6.30
	PA 38:4	[M-H]-	723.4965	6.06
	PA 38:4	[M-H]-	723.4965	7.98
	PA 40:6	[M-H]-	747.4965	8.05
**PC**	PC 28:0	[M+Hac-H]-	736.5129	5.20
	PC 29:0	[M+Hac-H]-	750.5285	5.56
	PC 30:0	[M+Hac-H]-	764.5442	5.87
	PC 30:1	[M+Hac-H]-	762.5285	5.41
	PC 30:2	[M+Hac-H]-	760.5129	5.05
	PC 30:3	[M+Hac-H]-	758.4972	4.66
	PC 31:0	[M+Hac-H]-	778.5598	6.23
	PC 32:0	[M+Hac-H]-	792.5755	6.50
	PC 32:1	[M+Hac-H]-	790.5598	6.01
	PC 32:2	[M+Hac-H]-	788.5442	5.58
	PC 32:3	[M+Hac-H]-	786.5285	5.41
	PC 32:4	[M+Hac-H]-	784.5129	4.94
	PC 33:0	[M+Hac-H]-	806.5911	6.81

	PC 33:1	[M+Hac-H]-	804.5755	6.37
	PC 33:2	[M+Hac-H]-	802.5598	5.89
	PC 34:1	[M+Hac-H]-	818.5911	6.61
	PC 34:2	[M+Hac-H]-	816.5755	6.18
	PC 34:3	[M+Hac-H]-	814.5598	5.82
	PC 34:4	[M+Hac-H]-	812.5442	5.49
	PC 34:5	[M+Hac-H]-	810.5285	5.03
	PC 35:1	[M+Hac-H]-	832.6068	6.94
	PC 35:2	[M+Hac-H]-	830.5911	6.49
	PC 35:3	[M+Hac-H]-	828.5755	6.04
	PC 35:4	[M+Hac-H]-	826.5598	5.87
	PC 36:0	[M+Hac-H]-	848.6381	7.69
	PC 36:1	[M+Hac-H]-	846.6224	7.19
	PC 36:2	[M+Hac-H]-	844.6068	6.78
	PC 36:3	[M+Hac-H]-	842.5911	6.38
	PC 36:4	[M+Hac-H]-	840.5755	6.12
	PC 36:5	[M+Hac-H]-	838.5598	5.70
	PC 36:6	[M+Hac-H]-	836.5442	5.39
	PC 36:7	[M+Hac-H]-	834.5285	5.01
	PC 37:2	[M+Hac-H]-	858.6224	7.09
	PC 37:3	[M+Hac-H]-	856.6068	6.68
	PC 37:4	[M+Hac-H]-	854.5911	6.50
	PC 37:6	[M+Hac-H]-	850.5598	5.78
	PC 38:1	[M+Hac-H]-	874.6537	7.73
	PC 38:2	[M+Hac-H]-	872.6381	7.33
	PC 38:3	[M+Hac-H]-	870.6224	7.04
	PC 38:4	[M+Hac-H]-	868.6068	6.73
	PC 38:5	[M+Hac-H]-	866.5911	6.30
	PC 38:6	[M+Hac-H]-	864.5755	5.97
	PC 38:7	[M+Hac-H]-	862.5598	5.58
	PC 38:8	[M+Hac-H]-	860.5442	5.15
	PC 38:9	[M+Hac-H]-	858.5285	4.90
	PC 39:6	[M+Hac-H]-	878.5911	6.39
	PC 40:1	[M+Hac-H]-	902.6850	8.24
	PC 40:10	[M+Hac-H]-	884.5442	4.97
	PC 40:3	[M+Hac-H]-	898.6537	7.50
	PC 40:4	[M+Hac-H]-	896.6381	7.24
	PC 40:5	[M+Hac-H]-	894.6224	7.00
	PC 40:6	[M+Hac-H]-	892.6068	6.70
	PC 40:7	[M+Hac-H]-	890.5911	6.16
	PC 40:8	[M+Hac-H]-	888.5755	5.78
	PC 40:9	[M+Hac-H]-	886.5598	5.39
	PC 42:10	[M+Hac-H]-	912.5755	5.69

	PC 42:11	[M+Hac-H]-	910.5598	5.27
	PC 42:4	[M+Hac-H]-	924.6694	7.71
	PC 42:6	[M+Hac-H]-	920.6381	7.34
	PC 42:7	[M+Hac-H]-	918.6224	6.80
	PC 42:8	[M+Hac-H]-	916.6068	6.36
	PC 42:9	[M+Hac-H]-	914.5911	5.99
	PC 44:10	[M+Hac-H]-	940.6068	6.18
	PC 44:11	[M+Hac-H]-	938.5911	5.90
	PC 44:12	[M+Hac-H]-	936.5755	5.57
	PC 44:4	[M+Hac-H]-	952.7007	8.05
	PC 44:6	[M+Hac-H]-	948.6694	7.83
	PC 44:9	[M+Hac-H]-	942.6224	6.51
	PC 48:10	[M+Hac-H]-	996.6694	7.17
**PE**	PE 30:0	[M-H]-	662.4761	5.21
	PE 30:0	[M-H]-	662.4761	6.06
	PE 32:0	[M-H]-	690.5074	5.89
	PE 32:0	[M-H]-	690.5074	6.69
	PE 32:1	[M-H]-	688.4917	6.17
	PE 32:2	[M-H]-	686.4761	5.72
	PE 33:1	[M-H]-	702.5074	6.53
	PE 33:2	[M-H]-	700.4917	6.05
	PE 34:1	[M-H]-	716.5230	6.81
	PE 34:2	[M-H]-	714.5074	5.09
	PE 34:2	[M-H]-	714.5074	6.37
	PE 34:3	[M-H]-	712.4917	6.00
	PE 34:4	[M-H]-	710.4761	5.71
	PE 35:1	[M-H]-	730.5387	7.10
	PE 35:2	[M-H]-	728.5230	6.66
	PE 35:3	[M-H]-	726.5074	6.19
	PE 35:4	[M-H]-	724.4917	6.07
	PE 36:0	[M-H]-	746.5700	7.80
	PE 36:1	[M-H]-	744.5543	7.38
	PE 36:2	[M-H]-	742.5387	6.09
	PE 36:2	[M-H]-	742.5387	6.94
	PE 36:3	[M-H]-	740.5230	5.27
	PE 36:3	[M-H]-	740.5230	6.54
	PE 36:4	[M-H]-	738.5074	6.27
	PE 36:5	[M-H]-	736.4917	5.89
	PE 36:6	[M-H]-	734.4761	5.59
	PE 37:2	[M-H]-	756.5543	7.24
	PE 37:4	[M-H]-	752.5230	6.68
	PE 37:5	[M-H]-	750.5074	6.18
	PE 38:1	[M-H]-	772.5856	7.87

	PE 38:2	[M-H]-	770.5700	7.46
	PE 38:3	[M-H]-	768.5543	7.21
	PE 38:4	[M-H]-	766.5387	6.87
	PE 38:5	[M-H]-	764.5230	6.51
	PE 38:6	[M-H]-	762.5074	6.17
	PE 38:7	[M-H]-	760.4917	5.75
	PE 39:4	[M-H]-	780.5543	7.08
	PE 40:1	[M-H]-	800.6169	8.42
	PE 40:10	[M-H]-	782.4761	5.15
	PE 40:2	[M-H]-	798.6013	7.93
	PE 40:3	[M-H]-	796.5856	7.65
	PE 40:4	[M-H]-	794.5700	7.38
	PE 40:5	[M-H]-	792.5543	7.20
	PE 40:6	[M-H]-	790.5387	6.88
	PE 40:6	[M-H]-	790.5387	8.69
	PE 40:7	[M-H]-	788.5230	6.36
	PE 40:8	[M-H]-	786.5074	5.89
	PE 40:9	[M-H]-	784.4917	5.58
	PE 42:1	[M-H]-	828.6482	8.84
	PE 42:10	[M-H]-	810.5074	5.81
	PE 42:11	[M-H]-	808.4917	5.43
	PE 42:2	[M-H]-	826.6326	8.41
	PE 42:4	[M-H]-	822.6013	8.05
	PE 42:6	[M-H]-	818.5700	7.45
	PE 42:7	[M-H]-	816.5543	6.95
	PE 42:9	[M-H]-	812.5230	6.12
	PE 44:10	[M-H]-	838.5387	6.33
	PE 44:12	[M-H]-	834.5074	5.72
	PE 44:2	[M-H]-	854.6639	8.83
	PE 44:4	[M-H]-	850.6326	8.50
	PE 44:9	[M-H]-	840.5543	6.70
	PE 46:10	[M-H]-	866.5700	6.84
**PG**	PG 30:0	[M-H]-	693.4707	5.21
	PG 32:0	[M-H]-	721.5020	5.85
	PG 32:1	[M-H]-	719.4863	5.34
	PG 33:0	[M-H]-	735.5176	6.14
	PG 34:1	[M-H]-	747.5176	5.94
	PG 34:2	[M-H]-	745.5020	5.38
	PG 34:3	[M-H]-	743.4863	4.72
	PG 34:4	[M-H]-	741.4707	4.49
	PG 36:0	[M-H]-	777.5646	6.98
	PG 36:2	[M-H]-	773.5333	5.90
	PG 36:3	[M-H]-	771.5176	5.36

	PG 36:3	[M-H]-	771.5176	5.75
	PG 36:4	[M-H]-	769.5020	4.89
	PG 36:4	[M-H]-	769.5020	5.54
	PG 36:5	[M-H]-	767.4863	4.79
	PG 37:4	[M-H]-	783.5176	5.82
	PG 38:2	[M-H]-	801.5646	6.35
	PG 38:3	[M-H]-	799.5489	5.92
	PG 38:3	[M-H]-	799.5489	6.38
	PG 38:4	[M-H]-	797.5333	5.59
	PG 38:4	[M-H]-	797.5333	6.15
	PG 38:5	[M-H]-	795.5176	5.19
	PG 38:5	[M-H]-	795.5176	5.62
	PG 38:6	[M-H]-	793.5020	4.85
	PG 38:6	[M-H]-	793.5020	5.43
	PG 38:7	[M-H]-	791.4863	4.54
	PG 40:4	[M-H]-	825.5646	5.98
	PG 40:5	[M-H]-	823.5489	5.76
	PG 40:6	[M-H]-	821.5333	5.53
	PG 40:7	[M-H]-	819.5176	5.15
	PG 40:8	[M-H]-	817.5020	4.78
	PG 42:10	[M-H]-	841.5020	4.75
	PG 42:6	[M-H]-	849.5646	5.87
	PG 42:7	[M-H]-	847.5489	5.66
	PG 42:8	[M-H]-	845.5333	5.35
	PG 42:9	[M-H]-	843.5176	4.99
	PG 44:10	[M-H]-	869.5333	5.23
	PG 44:11	[M-H]-	867.5176	4.99
	PG 44:12	[M-H]-	865.5020	4.63
	PG 44:9	[M-H]-	871.5489	5.52
**PI**	PI 34:1	[M-H]-	835.5337	5.82
	PI 34:2	[M-H]-	833.5180	5.35
	PI 36:1	[M-H]-	863.5650	6.42
	PI 36:2	[M-H]-	861.5493	5.98
	PI 36:3	[M-H]-	859.5337	5.57
	PI 36:4	[M-H]-	857.5180	5.31
	PI 38:3	[M-H]-	887.5650	6.21
	PI 38:4	[M-H]-	885.5493	5.94
	PI 38:5	[M-H]-	883.5337	5.48
	PI 40:4	[M-H]-	913.5806	6.40
	PI 40:5	[M-H]-	911.5650	6.05
	PI 40:6	[M-H]-	909.5493	5.91
**pPC**	pPC 34:0	[M+Hac-H]-	804.6119	7.29
	pPC 34:1	[M+Hac-H]-	802.5962	6.98

	pPC 34:2	[M+Hac-H]-	800.5806	6.55
	pPC 36:1	[M+Hac-H]-	830.6275	7.38
	pPC 36:2	[M+Hac-H]-	828.6119	7.05
	pPC 36:4	[M+Hac-H]-	824.5806	6.52
	pPC 37:4	[M+Hac-H]-	838.5962	6.75
	pPC 38:4	[M+Hac-H]-	852.6119	6.82
	pPC 38:4	[M+Hac-H]-	852.6119	7.15
	pPC 38:6	[M+Hac-H]-	848.5806	6.37
	pPC 40:4	[M+Hac-H]-	880.6431	7.43
	pPC 40:6	[M+Hac-H]-	876.6119	6.58
	pPC 42:4	[M+Hac-H]-	908.6744	7.74
**pPE**	pPE 32:0	[M-H]-	674.5125	7.05
	pPE 32:1	[M-H]-	672.4968	6.57
	pPE 32:2	[M-H]-	670.4812	6.05
	pPE 33:1	[M-H]-	686.5125	6.86
	pPE 34:0	[M-H]-	702.5438	7.60
	pPE 34:1	[M-H]-	700.5281	7.11
	pPE 34:2	[M-H]-	698.5125	6.72
	pPE 34:3	[M-H]-	696.4968	6.34
	pPE 34:4	[M-H]-	694.4812	6.02
	pPE 35:0	[M-H]-	716.5594	7.78
	pPE 35:1	[M-H]-	714.5438	7.35
	pPE 35:2	[M-H]-	712.5281	6.95
	pPE 35:4	[M-H]-	708.4968	6.28
	pPE 36:0	[M-H]-	730.5751	8.13
	pPE 36:1	[M-H]-	728.5594	6.96
	pPE 36:1	[M-H]-	728.5594	7.67
	pPE 36:2	[M-H]-	726.5438	7.26
	pPE 36:3	[M-H]-	724.5281	6.88
	pPE 36:4	[M-H]-	722.5125	6.63
	pPE 36:5	[M-H]-	720.4968	6.29
	pPE 36:6	[M-H]-	718.4812	5.95
	pPE 37:1	[M-H]-	742.5751	6.89
	pPE 37:1	[M-H]-	742.5751	7.87
	pPE 37:2	[M-H]-	740.5594	7.48
	pPE 37:3	[M-H]-	738.5438	7.10
	pPE 37:4	[M-H]-	736.5281	6.92
	pPE 37:5	[M-H]-	734.5125	6.53
	pPE 37:6	[M-H]-	732.4968	6.11
	pPE 38:1	[M-H]-	756.5907	8.16
	pPE 38:2	[M-H]-	754.5751	7.81
	pPE 38:3	[M-H]-	752.5594	7.53
	pPE 38:4	[M-H]-	750.5438	7.18

	pPE 38:4	[M-H]-	750.5438	8.63
	pPE 38:5	[M-H]-	748.5281	6.65
	pPE 38:6	[M-H]-	746.5125	6.56
	pPE 39:3	[M-H]-	766.5751	6.69
	pPE 39:4	[M-H]-	764.5594	7.38
	pPE 39:6	[M-H]-	760.5281	6.83
	pPE 40:1	[M-H]-	784.6220	8.58
	pPE 40:2	[M-H]-	782.6064	8.24
	pPE 40:3	[M-H]-	780.5907	8.01
	pPE 40:4	[M-H]-	778.5751	7.66
	pPE 40:5	[M-H]-	776.5594	7.31
	pPE 40:6	[M-H]-	774.5438	6.98
	pPE 40:6	[M-H]-	774.5438	8.79
	pPE 41:4	[M-H]-	792.5907	7.89
	pPE 42:4	[M-H]-	806.6064	8.10
	pPE 42:5	[M-H]-	804.5907	7.82
	pPE 42:6	[M-H]-	802.5751	7.71
**PS**	PS 34:1	[M-H]-	760.5129	5.96
	PS 34:2	[M-H]-	758.4972	5.44
	PS 36:1	[M-H]-	788.5442	6.52
	PS 36:2	[M-H]-	786.5285	6.02
	PS 36:3	[M-H]-	784.5129	5.83
	PS 36:4	[M-H]-	782.4972	5.43
	PS 38:1	[M-H]-	816.5755	7.16
	PS 38:2	[M-H]-	814.5598	6.55
	PS 38:3	[M-H]-	812.5442	6.30
	PS 38:4	[M-H]-	810.5285	6.02
	PS 38:5	[M-H]-	808.5129	5.55
	PS 38:6	[M-H]-	806.4972	5.31
	PS 40:1	[M-H]-	844.6068	7.64
	PS 40:2	[M-H]-	842.5911	7.10
	PS 40:4	[M-H]-	838.5598	6.52
	PS 40:5	[M-H]-	836.5442	6.31
	PS 40:6	[M-H]-	834.5285	5.97
	PS 40:7	[M-H]-	832.5129	5.47
	PS 40:8	[M-H]-	830.4972	4.97
	PS 42:1	[M-H]-	872.6381	8.11
	PS 42:10	[M-H]-	854.4972	4.91
	PS 42:6	[M-H]-	862.5598	6.59
	PS 42:7	[M-H]-	860.5442	6.05
	PS 42:8	[M-H]-	858.5285	5.68
	PS 42:9	[M-H]-	856.5129	5.20
	PS 44:10	[M-H]-	882.5285	5.35

	PS 44:11	[M-H]-	880.5129	5.00
	PS 44:12	[M-H]-	878.4972	4.79
	PS 44:6	[M-H]-	890.5911	7.15
	PS 44:7	[M-H]-	888.5755	6.65
	PS 44:9	[M-H]-	884.5442	5.85
	PS 46:10	[M-H]-	910.5598	5.94
	PS 48:10	[M-H]-	938.5911	6.48
**SM**	SM 30:1	[M+Hac-H]-	705.5183	4.33
	SM 31:1	[M+Hac-H]-	719.5339	4.72
	SM 32:0	[M+Hac-H]-	735.5652	5.41
	SM 32:1	[M+Hac-H]-	733.5496	5.09
	SM 32:2	[M+Hac-H]-	731.5339	4.47
	SM 33:1	[M+Hac-H]-	747.5652	5.47
	SM 33:2	[M+Hac-H]-	745.5496	4.98
	SM 34:0	[M+Hac-H]-	763.5965	6.06
	SM 34:1	[M+Hac-H]-	761.5809	5.76
	SM 34:2	[M+Hac-H]-	759.5652	5.26
	SM 35:2	[M+Hac-H]-	773.5809	5.64
	SM 36:0	[M+Hac-H]-	791.6278	6.70
	SM 36:1	[M+Hac-H]-	789.6122	6.46
	SM 36:2	[M+Hac-H]-	787.5965	5.96
	SM 36:3	[M+Hac-H]-	785.5809	5.57
	SM 37:1	[M+Hac-H]-	803.6278	6.82
	SM 38:1	[M+Hac-H]-	817.6435	7.07
	SM 38:2	[M+Hac-H]-	815.6278	6.67
	SM 39:1	[M+Hac-H]-	831.6591	7.43
	SM 40:0	[M+Hac-H]-	847.6904	7.87
	SM 40:1	[M+Hac-H]-	845.6748	7.65
	SM 40:2	[M+Hac-H]-	843.6591	7.24
	SM 40:3	[M+Hac-H]-	841.6435	6.71
	SM 41:1	[M+Hac-H]-	859.6904	7.93
	SM 41:2	[M+Hac-H]-	857.6748	7.50
	SM 42:0	[M+Hac-H]-	875.7217	8.37
	SM 42:1	[M+Hac-H]-	873.7061	8.14
	SM 42:2	[M+Hac-H]-	871.6904	7.69
	SM 42:3	[M+Hac-H]-	869.6748	7.27
	SM 42:4	[M+Hac-H]-	867.6591	6.85
	SM 43:1	[M+Hac-H]-	887.7217	8.39
	SM 44:1	[M+Hac-H]-	901.7374	8.63
	SM 44:2	[M+Hac-H]-	899.7217	8.16

Table S3: Comparing z-score standardized mean values of lipid factors in study groups in central and peripheral retina

		Central Retina			Peripheral Retina		
Lipids	Group	Mean	SE	p value	Mean	SE	p value
AC (C≥14)	No DM	0.231	0.043	***<0.0001***	-0.138	0.053	0.4600
	DM, No DR	0.390	0.041	***<0.0001***	-0.164	0.066	0.8160
	DM, with DR	-0.123	0.059	Ref	-0.256	0.057	Ref
uFFA	No DM	0.354	0.055	***0.004***	-0.064	0.058	0.571
	DM, No DR	0.088	0.056	0.9937	-0.467	0.063	***<0.0001***
	DM, with DR	0.079	0.075	Ref	0.024	0.081	Ref
FAHFA	No DM	0.117	0.037	***0.001***	-0.028	0.039	0.650
	DM, No DR	0.168	0.039	***<0.0001***	-0.204	0.042	***0.0003***
	DM, with DR	-0.068	0.038	Ref	0.019	0.045	Ref
DAG	No DM	0.160	0.032	***<0.0001***	0.008	0.038	0.105
	DM, No DR	0.110	0.032	***0.001***	-0.096	0.036	0.991
	DM, with DR	-0.072	0.044	Ref	-0.102	0.047	Ref
TAG	No DM	0.070	0.019	***<0.0001***	0.170	0.022	***<0.0001***
	DM, No DR	0.053	0.025	***<0.0001***	0.004	0.025	***0.007***
	DM, with DR	-0.183	0.031	Ref	-0.103	0.015	Ref
PC	No DM	0.145	0.019	***<0.0001***	0.051	0.019	***<0.0001***
	DM, No DR	0.062	0.018	***<0.0001***	0.005	0.019	***<0.0001***
	DM, with DR	-0.115	0.030	Ref	-0.138	0.028	Ref
Cer[NS]	No DM	-0.143	0.041	***<0.0001***	-0.134	0.039	***<0.0001***
	DM, No DR	-0.055	0.043	***0.003***	-0.039	0.040	***0.002***
	DM, with DR	0.165	0.061	Ref	0.193	0.066	Ref
ClucCer	No DM	0.093	0.068	0.064	0.065	0.067	0.049
	DM, No DR	0.128	0.068	0.025	0.001	0.066	0.194
	DM, with DR	-0.125	0.080	Ref	-0.153	0.073	Ref
PG	No DM	0.075	0.040	***0.008***	-0.039	0.036	0.698
	DM, No DR	0.036	0.035	0.052	0.008	0.037	0.997
	DM, with DR	-0.088	0.044	Ref	0.005	0.041	Ref
CE	No DM	0.057	0.076	0.0370	0.075	0.065	0.381
	DM, No DR	0.113	0.066	***0.007***	-0.014	0.080	0.930
	DM, with DR	-0.179	0.071	Ref	-0.045	0.064	Ref
pPC	No DM	0.049	0.031	0.099	0.017	0.030	0.808
	DM, No DR	0.040	0.028	0.137	-0.049	0.030	0.660
	DM, with DR	-0.045	0.042	Ref	-0.010	0.042	Ref
PI	No DM	0.034	0.053	0.3180	-0.059	0.050	0.4900
	DM, No DR	-0.061	0.055	0.0360	-0.109	0.058	0.2150
	DM, with DR	0.152	0.079	Ref	0.035	0.084	Ref
Cer[NDS]	No DM	-0.163	0.081	0.219	0.059	0.062	0.393
	DM, No DR	-0.169	0.072	0.195	0.018	0.062	0.216
	DM, with DR	0.050	0.129	Ref	0.200	0.115	Ref
BMP	No DM	0.079	0.057	0.886	-0.070	0.052	0.027
	DM, No DR	-0.056	0.051	0.298	-0.131	0.065	***0.002***
	DM, with DR	0.048	0.056	Ref	0.130	0.054	Ref

PE	No DM	-0.057	0.028	0.339	0.029	0.026	0.213
	DM, No DR	-0.089	0.026	0.091	0.015	0.025	0.112
	DM, with DR	0.002	0.042	Ref	0.098	0.041	Ref
pPE	No DM	0.120	0.036	0.784	-0.093	0.031	0.861
	DM, No DR	0.109	0.035	0.652	-0.168	0.035	0.578
	DM, with DR	0.154	0.050	Ref	-0.119	0.051	Ref
PS	No DM	0.002	0.031	0.680	0.012	0.028	0.262
	DM, No DR	0.017	0.028	0.870	-0.007	0.028	0.451
	DM, with DR	0.039	0.045	Ref	-0.063	0.050	Ref
SM	No DM	-0.043	0.035	0.279	0.022	0.031	0.838
	DM, No DR	0.007	0.033	0.878	-0.013	0.034	0.974
	DM, with DR	0.029	0.041	Ref	-0.003	0.042	Ref
Cer[AS]	No DM	0.031	0.150	0.809	-0.036	0.199	0.982
	DM, No DR	0.077	0.121	0.669	0.018	0.141	0.996
	DM, with DR	-0.091	0.193	Ref	0.001	0.148	Ref
Cer[EDOS]	No DM	-0.003	0.092	0.972	-0.017	0.100	0.996
	DM, No DR	-0.023	0.095	0.916	0.021	0.084	0.975
	DM, with DR	0.026	0.110	Ref	-0.005	0.119	Ref
Cer[NP]	No DM	0.062	0.084	0.891	0.023	0.081	0.844
	DM, No DR	0.016	0.085	0.999	-0.066	0.096	0.978
	DM, with DR	0.012	0.097	Ref	-0.043	0.104	Ref
LPC	No DM	-0.032	0.046	1.000	0.062	0.047	0.945
	DM, No DR	-0.099	0.049	0.525	0.021	0.047	0.605
	DM, with DR	-0.032	0.054	Ref	0.082	0.056	Ref
LPE	No DM	-0.027	0.059	0.980	-0.031	0.063	0.782
	DM, No DR	0.016	0.058	0.937	0.023	0.057	0.999
	DM, with DR	-0.012	0.077	Ref	0.028	0.086	Ref
PA	No DM	0.030	0.082	0.999	0.012	0.080	0.662
	DM, No DR	0.001	0.091	0.974	0.023	0.087	0.596
	DM, with DR	0.025	0.097	Ref	-0.089	0.109	Ref

CE, cholesterol Esters; BMP, bis(monoacylglycero)phosphate; Cer[AS], ceramide class consisting of α-hydroxy fatty acids and 4-sphingenines; Cer[EOS], omega-acylceramide; Cer[EODS], ceramide class consisting of ester-linked non-hydroxy fatty acids, ω-hydroxy fatty acids and 4-sphinnenines; Cer[NDS], non-hydroxy fatty acids and sphinganine; Cer[NDS], ceramide class consisting of non-hydroxy fatty acids and sphinganines; Cer[NP], ceramide class consisting of non-hydroxy fatty acids and 4-hydroxysphinganines; Cer[NS], non-hydroxy fatty acids and 4-sphingenine ceramide; DAG, diacylglycerol; GlcCer[NS], ceramide class consisting of non-hydroxy fatty acids and 4-sphingenines; FAHFA, Fatty Acid ester of Hydroxyl Fatty Acid; LPC, lysophosphatidylcholine; LPE, lysophosphatidylethanolamine; PC, phosphatidylcholine; pPC, plasmenyl-phosphatidylcholine; PE, phosphatidylethanolamine; pPE: plasmenyl-phosphatidylethanolamine; PS, phosphatidylserine; PA, phosphatidic acid; SM, sphingomyelin; TAG, triacylglycerol; AC, acylcarnitine; FFA, free Fatty Acid; PG, phosphatidylglycerol; PI, phosphatidylinositol

Fig S1: Comparing standardized mean values of the study lipids by location of retina and study groups in the postmortem cohort. Values are means and 95% confidence intervals. The p value shows the significance of mean values by the main effect of location (comparing central versus peripheral retina).

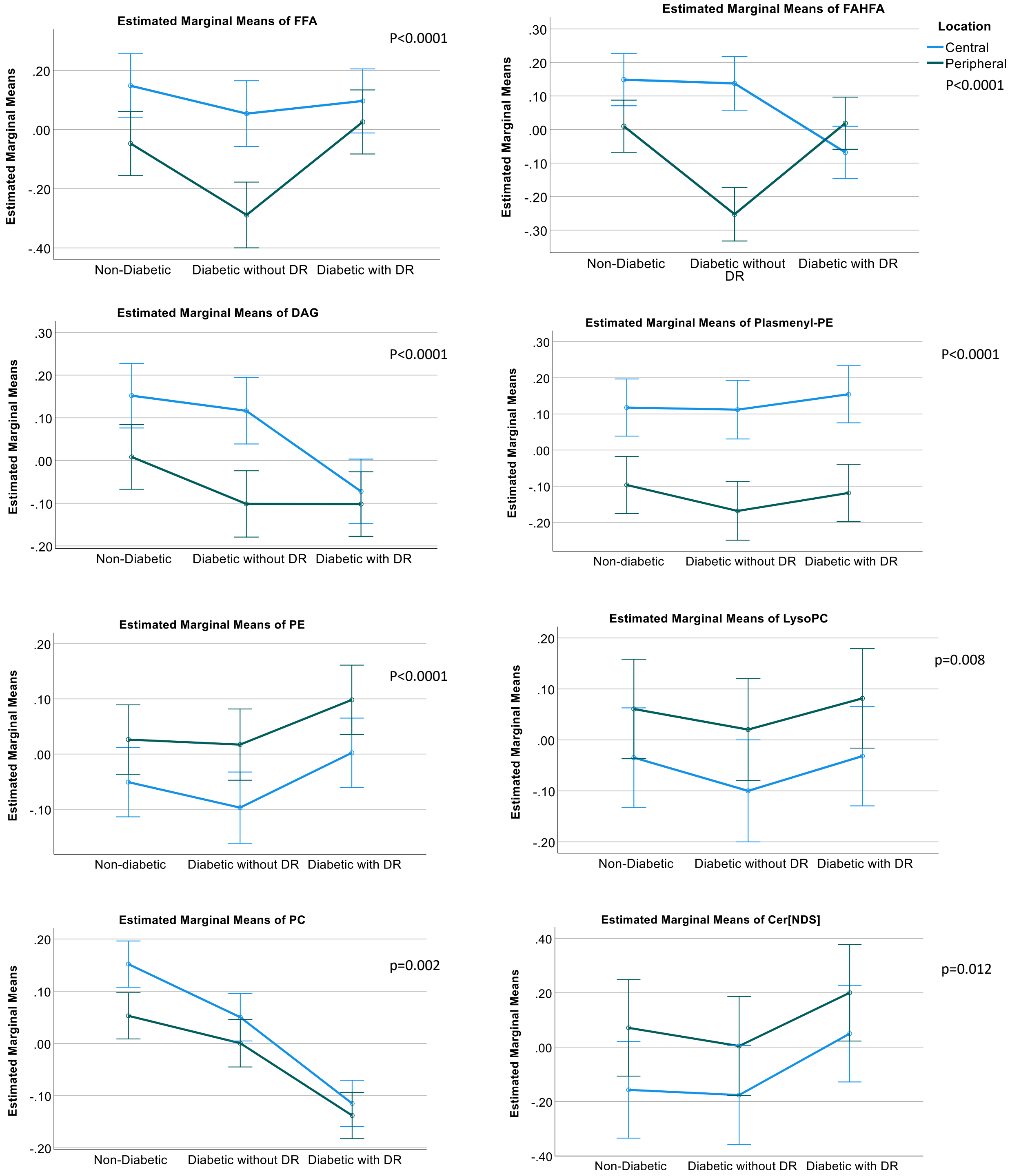

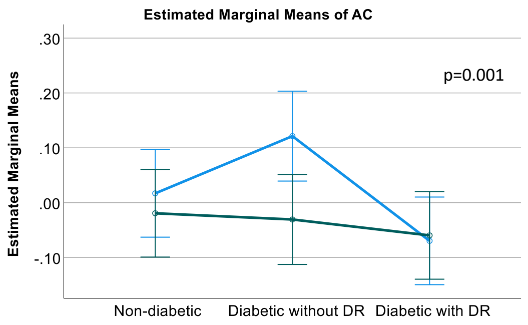
Estimated Marginal Means of AC
p=0.001
Estimated Marginal Means
.30
.20
.10
.00
-.10
Non-diabetic
Diabetic without DR
Diabetic with DR

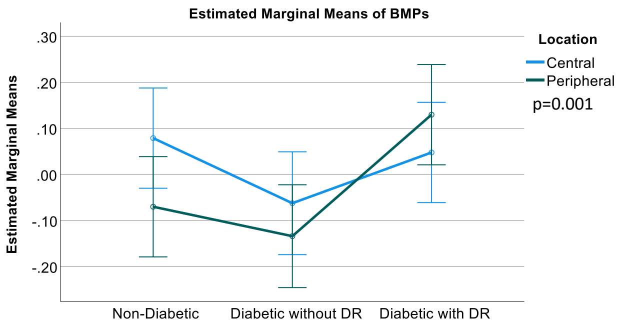
Estimated Marginal Means of BMPs
Location
Central
Peripheral
p=0.001
Estimated Marginal Means
.30
.20
.10
.00
-.10
-.20
Non-Diabetic
Diabetic without DR
Diabetic with DR

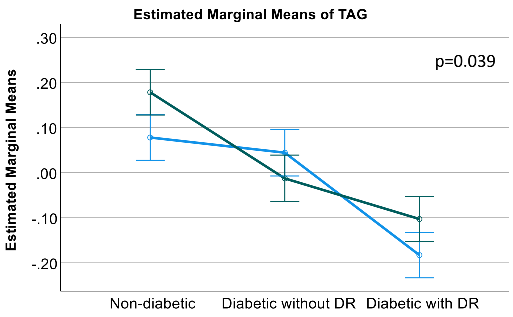
Estimated Marginal Means of TAG
p=0.039
Estimated Marginal Means
.30
.20
.10
.00
-.10
-.20
Non-diabetic
Diabetic without DR
Diabetic with DR

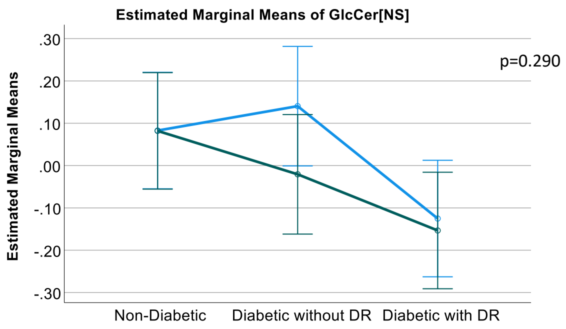
Estimated Marginal Means of GlcCer[NS]
p=0.290
Estimated Marginal Means
.30
.20
.10
.00
-.10
-.20
-.30
Non-Diabetic
Diabetic without DR
Diabetic with DR

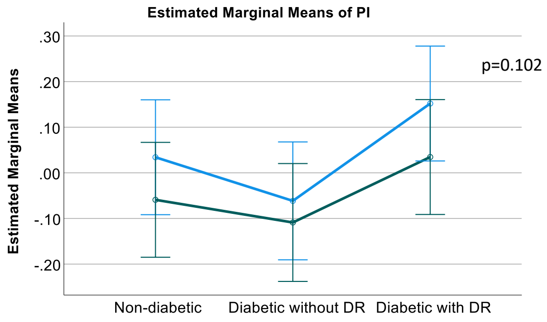
Estimated Marginal Means of PI
p=0.102
Estimated Marginal Means
.30
.20
.10
.00
-.10
-.20
Non-diabetic
Diabetic without DR
Diabetic with DR

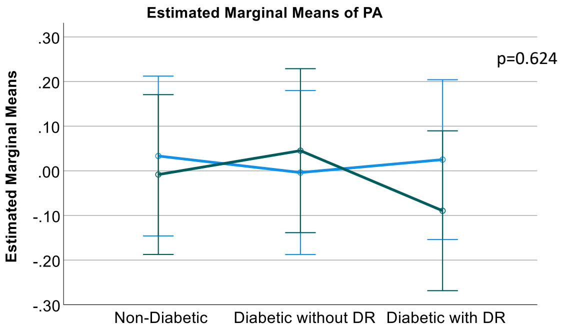
Estimated Marginal Means of PA
p=0.624
Estimated Marginal Means
.30
.20
.10
.00
-.10
-.20
-.30
Non-Diabetic
Diabetic without DR
Diabetic with DR

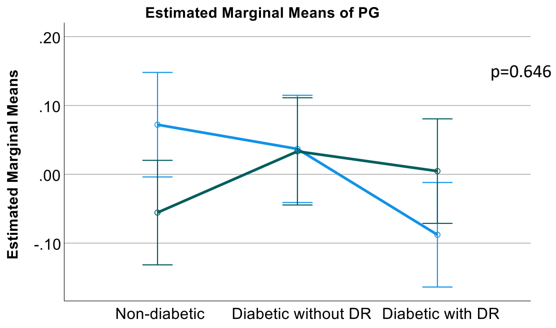
Estimated Marginal Means of PG
p=0.646
Estimated Marginal Means
.20
.10
.00
-.10
Non-diabetic
Diabetic without DR
Diabetic with DR

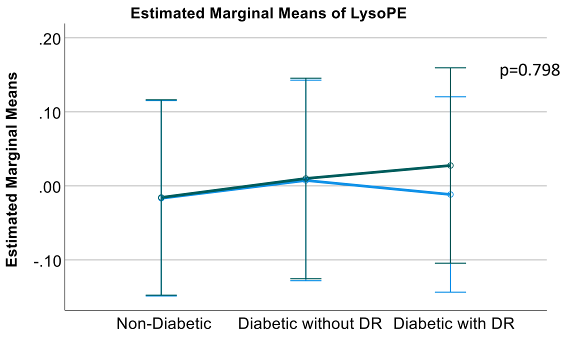
Estimated Marginal Means of LysoPE
p=0.798
Estimated Marginal Means
.20
.10
.00
-.10
Non-Diabetic
Diabetic without DR
Diabetic with DR

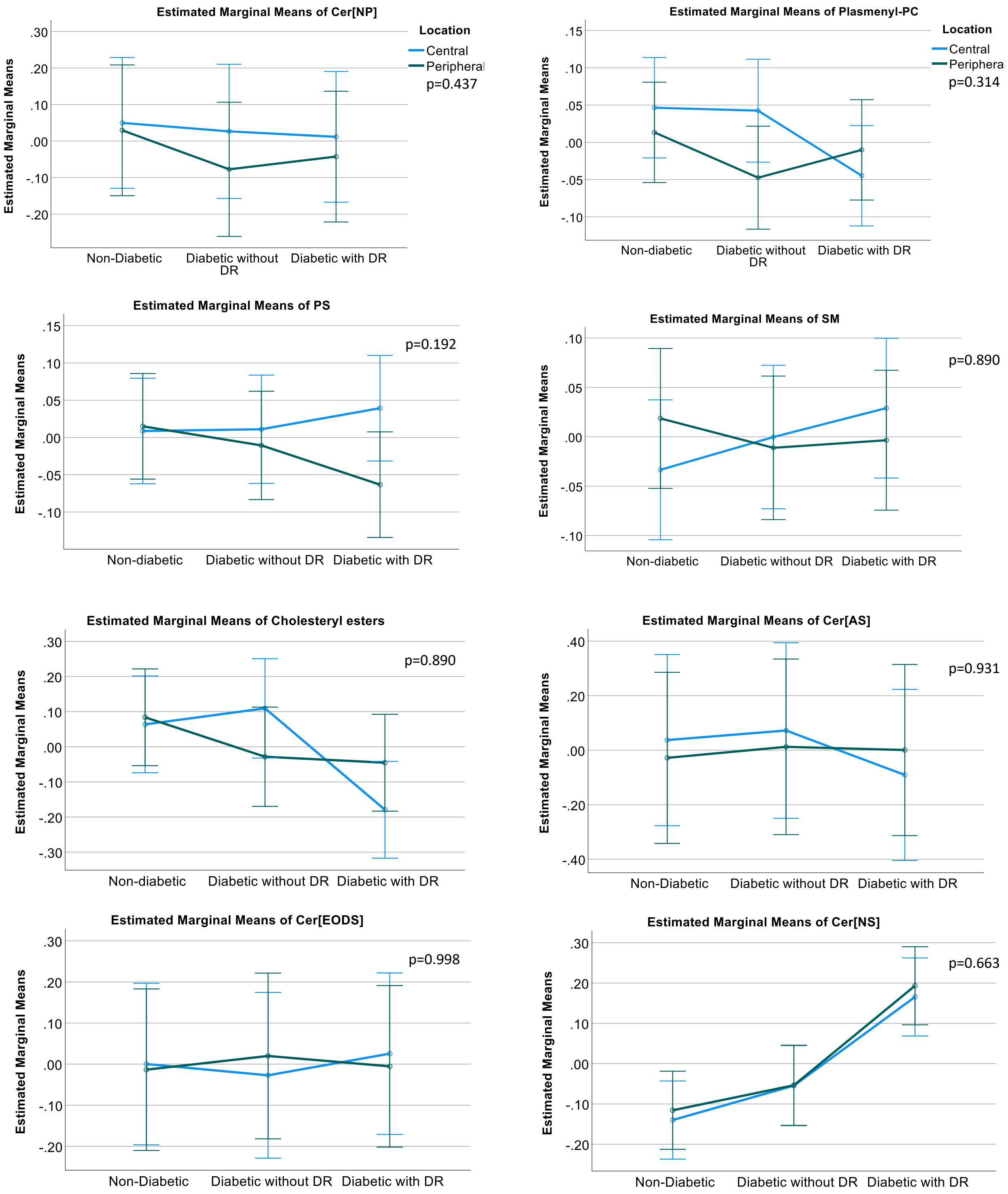

Estimated Marginal Means of Cer[NP]
Location
Central
Peripheral
p=0.437
Estimated Marginal Means
.30
.20
.10
.00
-.10
-.20
Non-Diabetic
Diabetic without DR
Diabetic with DR
Estimated Marginal Means of Plasmenyl-PC
Location
Central
Peripheral
p=0.314
.15
.10
.05
.00
-.05
-.10
Non-diabetic
Diabetic without DR
Diabetic with DR
Estimated Marginal Means of PS
p=0.192
Estimated Marginal Means of SM
p=0.890
Estimated Marginal Means of Cholesteryl esters
p=0.890
Estimated Marginal Means of Cer[AS]
p=0.931
.40
-.40
Estimated Marginal Means of Cer[EODS]
p=0.998
Estimated Marginal Means of Cer[NS]
p=0.663

Table S4: Partial correlation coefficient of ACs, FFAs, and complex glycerolipids in the central retina of post-mortem cohort controlled by group membership. CR: central retina; PR: peripheral retina; sFFA: saturated free fatty acids; uFFA: unsaturated free fatty acids; FAHFA: Fatty Acid ester of Hydroxyl Fatty Acid; AC: acylcarnitines; DAG: diacylglycerols; PC: phosphatidylcholines; TAG: triacylglycerols

A)

		sFFA	usFFA	FAHFA =<36:3	FAHFA >=38:4	DAG long	DAG short	TAG Long	TAG Short	TAG Intrm	PC Long PolyUnsat	PC Short	AC >=14	AC <14
CR	sFFA	1.000	-0.614	0.076	-0.375	-0.163	-0.190	0.000	0.243	0.219	-0.126	-0.122	-0.028	-0.034
	usFFA	-0.614	1.000	-0.162	0.534	0.318	0.214	0.334	-0.335	-0.295	0.394	0.306	0.072	0.124
	FAHFA =<36:3	0.076	-0.162	1.000	0.558	0.000	0.268	-0.130	0.074	-0.010	-0.098	-0.146	0.154	-0.169
	FAHFA >=38:4	-0.375	0.534	0.558	1.000	0.345	0.298	0.230	-0.238	-0.167	0.290	0.077	0.175	-0.089
	DAG long	-0.163	0.318	0.000	0.345	1.000	-0.006	0.346	-0.303	0.073	0.600	0.240	0.034	0.003
	DAG short	-0.190	0.214	0.268	0.298	-0.006	1.000	-0.182	-0.065	-0.124	-0.246	-0.091	0.244	-0.081
	TAG Long	0.000	0.334	-0.130	0.230	0.346	-0.182	1.000	-0.018	0.018	0.435	0.002	-0.025	0.113
	TAG Short	0.243	-0.335	0.074	-0.238	-0.303	-0.065	-0.018	1.000	0.006	-0.180	-0.153	-0.084	-0.115
	TAG Intrm	0.219	-0.295	-0.010	-0.167	0.073	-0.124	0.018	0.006	1.000	0.022	0.398	-0.451	0.281
	PC Long PolyUnsat	-0.126	0.394	-0.098	0.290	0.600	-0.246	0.435	-0.180	0.022	1.000	0.113	-0.174	0.124
	PC Short	-0.122	0.306	-0.146	0.077	0.240	-0.091	0.002	-0.153	0.398	0.113	1.000	-0.391	0.231
	AC >=14	-0.028	0.072	0.154	0.175	0.034	0.244	-0.025	-0.084	-0.451	-0.174	-0.391	1.000	-0.687
	AC <14	-0.034	0.124	-0.169	-0.089	0.003	-0.081	0.113	-0.115	0.281	0.124	0.231	-0.687	1.000
PR	sFFA	1.000	-0.790	0.113	-0.625	-0.393	-0.309	-0.150	0.559	0.201	-0.444	-0.200	-0.073	0.278
	usFFA	-0.790	1.000	0.076	0.727	0.269	0.342	0.084	-0.425	-0.124	0.469	0.417	-0.030	-0.094
	FAHFA =<36:3	0.113	0.076	1.000	0.436	-0.330	0.030	0.083	0.222	-0.041	-0.232	0.147	0.184	-0.017
	FAHFA >=38:4	-0.625	0.727	0.436	1.000	0.266	0.301	0.291	-0.257	-0.161	0.481	0.246	0.006	-0.280
	DAG long	-0.393	0.269	-0.330	0.266	1.000	-0.006	0.255	-0.517	0.280	0.770	0.214	0.002	-0.337
	DAG short	-0.309	0.342	0.030	0.301	-0.006	1.000	-0.092	-0.204	-0.313	-0.017	-0.149	0.052	-0.080
	TAG Long	-0.150	0.084	0.083	0.291	0.255	-0.092	1.000	-0.044	0.000	0.401	-0.191	0.129	-0.269
	TAG Short	0.559	-0.425	0.222	-0.257	-0.517	-0.204	-0.044	1.000	0.000	-0.462	-0.197	0.101	0.019
	TAG Intrm	0.201	-0.124	-0.041	-0.161	0.280	-0.313	0.000	0.000	1.000	0.092	0.478	-0.214	-0.062
	PC Long PolyUnsat	-0.444	0.469	-0.232	0.481	0.770	-0.017	0.401	-0.462	0.092	1.000	0.128	-0.107	-0.321
	PC Short	-0.200	0.417	0.147	0.246	0.214	-0.149	-0.191	-0.197	0.478	0.128	1.000	-0.288	0.107
	AC >=14	-0.073	-0.030	0.184	0.006	0.002	0.052	0.129	0.101	-0.214	-0.107	-0.288	1.000	-0.277
	AC <14	0.278	-0.094	-0.017	-0.280	-0.337	-0.080	-0.269	0.019	-0.062	-0.321	0.107	-0.277	1.000

B)

CR	sFFA		2.91E-07	5.69E-01	3.71E-03	2.23E-01	1.54E-01	1.00E+00	6.57E-02	9.83E-02	3.46E-01	3.62E-01	8.34E-01	8.03E-01
	usFFA	2.91E-07		2.24E-01	1.56E-05	1.51E-02	1.07E-01	1.05E-02	1.01E-02	2.46E-02	2.23E-03	1.94E-02	5.91E-01	3.55E-01
	FAHFA =<36:3	5.69E-01	2.24E-01		5.42E-06	9.99E-01	4.20E-02	3.31E-01	5.80E-01	9.42E-01	4.64E-01	2.76E-01	2.48E-01	2.05E-01
	FAHFA >=38:4	3.71E-03	1.56E-05	5.42E-06		8.07E-03	2.30E-02	8.27E-02	7.19E-02	2.11E-01	2.70E-02	5.65E-01	1.89E-01	5.09E-01
	DAG long	2.23E-01	1.51E-02	9.99E-01	8.07E-03		9.63E-01	7.90E-03	2.06E-02	5.84E-01	6.51E-07	6.95E-02	8.01E-01	9.84E-01
	DAG short	1.54E-01	1.07E-01	4.20E-02	2.30E-02	9.63E-01		1.72E-01	6.29E-01	3.54E-01	6.27E-02	4.99E-01	6.44E-02	5.46E-01
	TAG Long	1.00E+00	1.05E-02	3.31E-01	8.27E-02	7.90E-03	1.72E-01		8.92E-01	8.95E-01	6.39E-04	9.85E-01	8.51E-01	3.98E-01
	TAG Short	6.57E-02	1.01E-02	5.80E-01	7.19E-02	2.06E-02	6.29E-01	8.92E-01		9.65E-01	1.76E-01	2.51E-01	5.33E-01	3.88E-01
	TAG Intrm	9.83E-02	2.46E-02	9.42E-01	2.11E-01	5.84E-01	3.54E-01	8.95E-01	9.65E-01		8.68E-01	2.00E-03	3.80E-04	3.29E-02
	PC Long PolyUnsat	3.46E-01	2.23E-03	4.64E-01	2.70E-02	6.51E-07	6.27E-02	6.39E-04	1.76E-01	8.68E-01		3.98E-01	1.91E-01	3.54E-01
	PC Short	3.62E-01	1.94E-02	2.76E-01	5.65E-01	6.95E-02	4.99E-01	9.85E-01	2.51E-01	2.00E-03	3.98E-01		2.43E-03	8.11E-02
	AC >=14	8.34E-01	5.91E-01	2.48E-01	1.89E-01	8.01E-01	6.44E-02	8.51E-01	5.33E-01	3.80E-04	1.91E-01	2.43E-03		2.61E-09
	AC <14	8.03E-01	3.55E-01	2.05E-01	5.09E-01	9.84E-01	5.46E-01	3.98E-01	3.88E-01	3.29E-02	3.54E-01	8.11E-02	2.61E-09	
PR	sFFA		1.75E-13	3.97E-01	1.60E-07	2.30E-03	1.82E-02	2.62E-01	5.05E-06	1.30E-01	4.82E-04	1.33E-01	5.86E-01	3.46E-02
	usFFA	1.75E-13		5.71E-01	1.03E-10	4.13E-02	8.60E-03	5.31E-01	8.75E-04	3.55E-01	2.04E-04	1.13E-03	8.24E-01	4.82E-01
	FAHFA =<36:3	3.97E-01	5.71E-01		6.21E-04	1.14E-02	8.23E-01	5.36E-01	9.47E-02	7.60E-01	7.99E-02	2.72E-01	1.66E-01	9.00E-01
	FAHFA >=38:4	1.60E-07	1.03E-10	6.21E-04		4.38E-02	2.16E-02	2.66E-02	5.19E-02	2.28E-01	1.34E-04	6.28E-02	9.64E-01	3.35E-02
	DAG long	2.30E-03	4.13E-02	1.14E-02	4.38E-02		9.62E-01	5.35E-02	3.22E-05	3.33E-02	1.60E-12	1.07E-01	9.86E-01	9.71E-03
	DAG short	1.82E-02	8.60E-03	8.23E-01	2.16E-02	9.62E-01		4.94E-01	1.24E-01	1.66E-02	9.01E-01	2.64E-01	6.97E-01	5.53E-01
	TAG Long	2.62E-01	5.31E-01	5.36E-01	2.66E-02	5.35E-02	4.94E-01		7.45E-01	9.99E-01	1.83E-03	1.52E-01	3.33E-01	4.09E-02
	TAG Short	5.05E-06	8.75E-04	9.47E-02	5.19E-02	3.22E-05	1.24E-01	7.45E-01		9.99E-01	2.65E-04	1.37E-01	4.50E-01	8.88E-01
	TAG Intrm	1.30E-01	3.55E-01	7.60E-01	2.28E-01	3.33E-02	1.66E-02	9.99E-01	9.99E-01		4.93E-01	1.50E-04	1.06E-01	6.46E-01
	PC Long PolyUnsat	4.82E-04	2.04E-04	7.99E-02	1.34E-04	1.60E-12	9.01E-01	1.83E-03	2.65E-04	4.93E-01		3.38E-01	4.23E-01	1.39E-02
	PC Short	1.33E-01	1.13E-03	2.72E-01	6.28E-02	1.07E-01	2.64E-01	1.52E-01	1.37E-01	1.50E-04	3.38E-01		2.86E-02	4.24E-01
	AC >=14	5.86E-01	8.24E-01	1.66E-01	9.64E-01	9.86E-01	6.97E-01	3.33E-01	4.50E-01	1.06E-01	4.23E-01	2.86E-02		3.50E-02
	AC <14	3.46E-02	4.82E-01	9.00E-01	3.35E-02	9.71E-03	5.53E-01	4.09E-02	8.88E-01	6.46E-01	1.39E-02	4.24E-01	3.50E-02	

Fig S2: Comparing inter- and intra-assay coefficients of variation of internal standards in test pools and pooled plasma in Pima Indians.

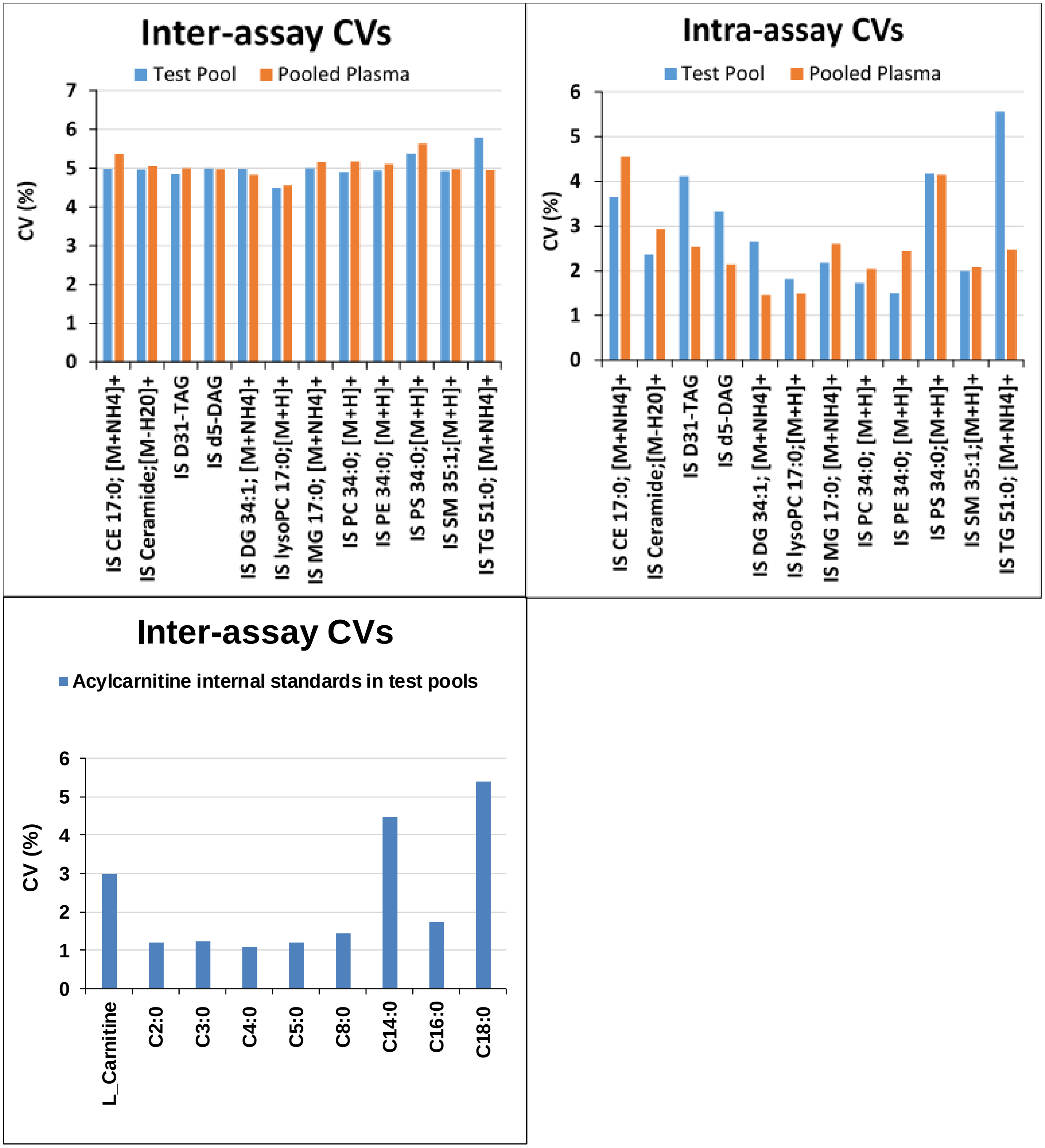

Fig S3: Illustration of minimal batch to batch variation in Pima Indians.

A) Distribution of lipidomic platform internal standards in test pools in sequential runs across different batches

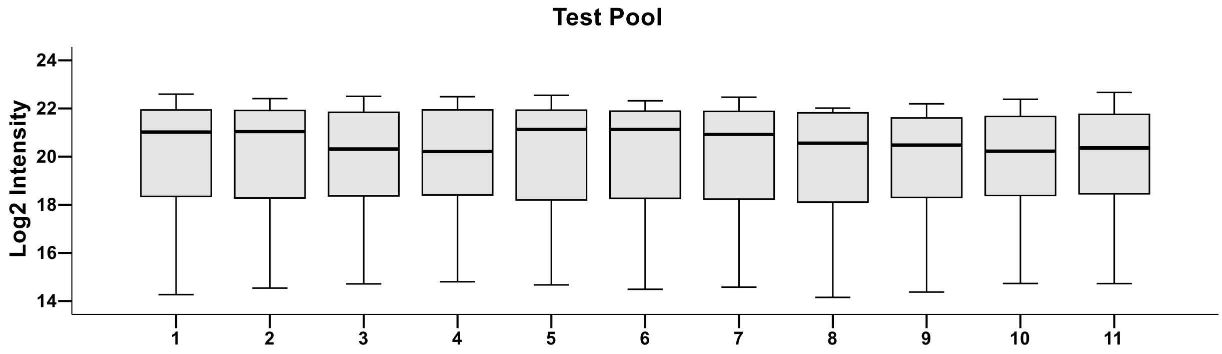

B) Distribution of lipidomic platform internal standards in pooled plasma in sequential runs across different batches

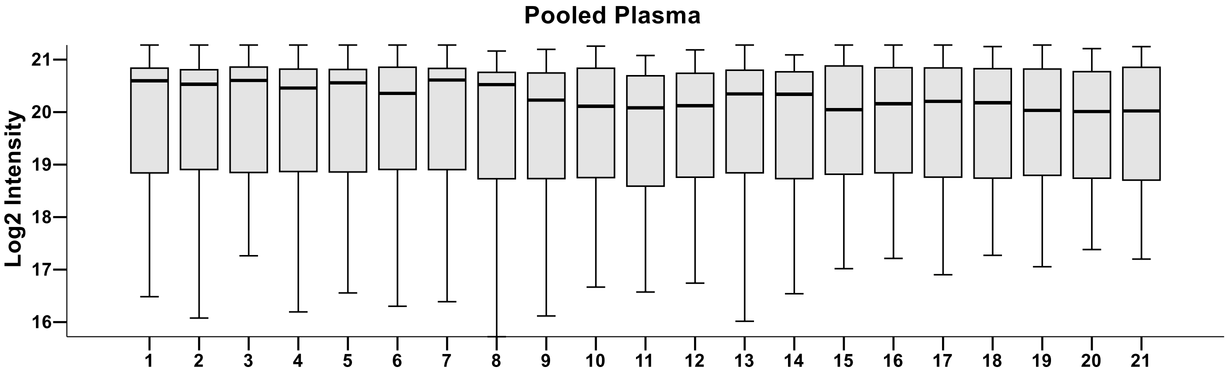

C) Distribution of acylcarnitine internal standards in pooled plasma in sequential runs across different batches

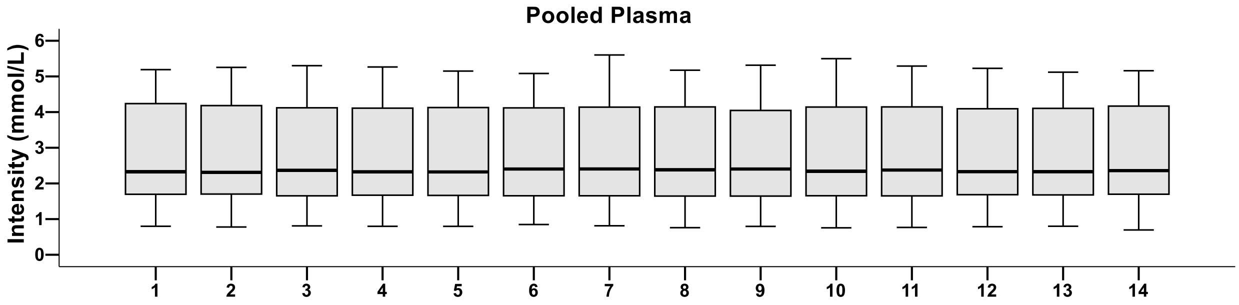

Fig S4: Comparing inter- and intra-assay coefficients of variation of internal standards in test pools and pooled plasma in postmortem cohort.

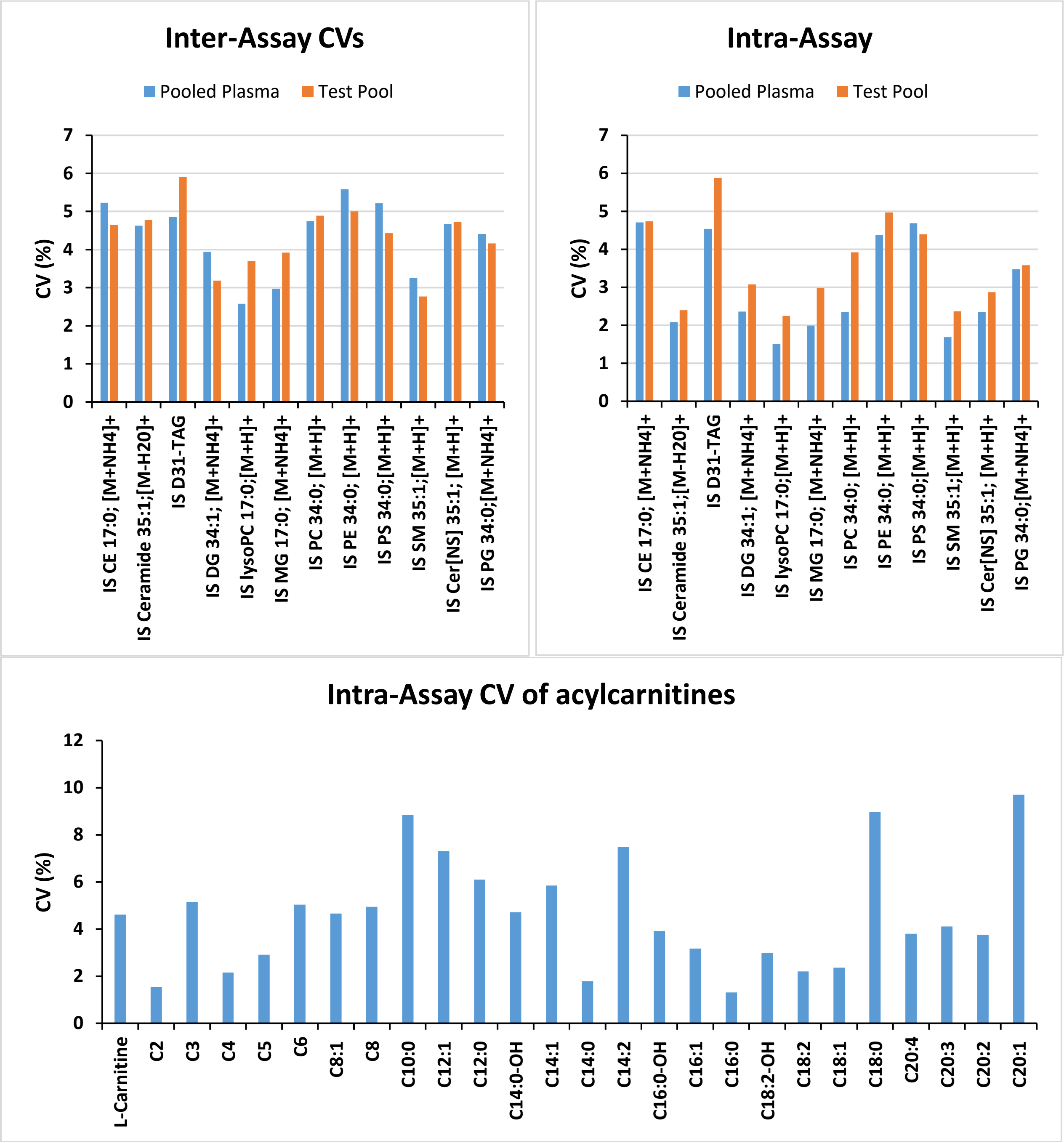

Fig S5: Illustration of minimal batch to batch variation in postmortem cohort.

A) Distribution of lipidomic internal standards in pooled plasma in sequential runs across different batches

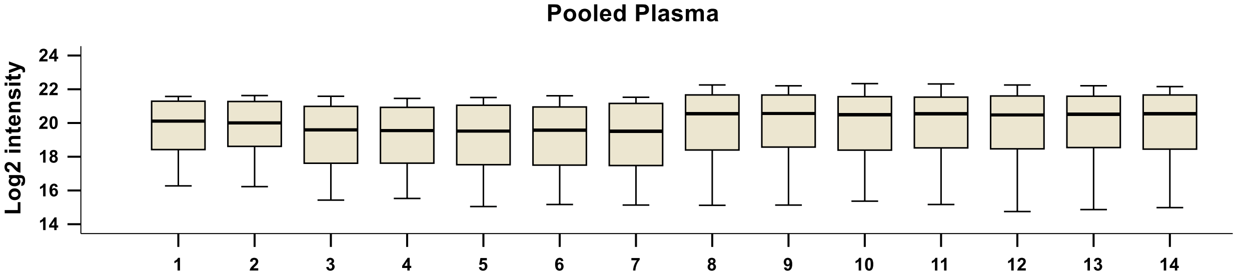

B) Distribution of lipidomic internal standards in test pools in sequential runs across different batches

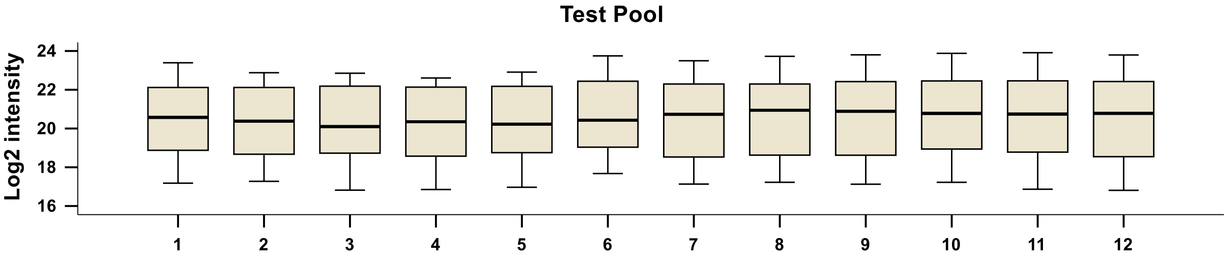

C) Distribution of acylcarnitine internal standards in Test Pools in sequential runs across

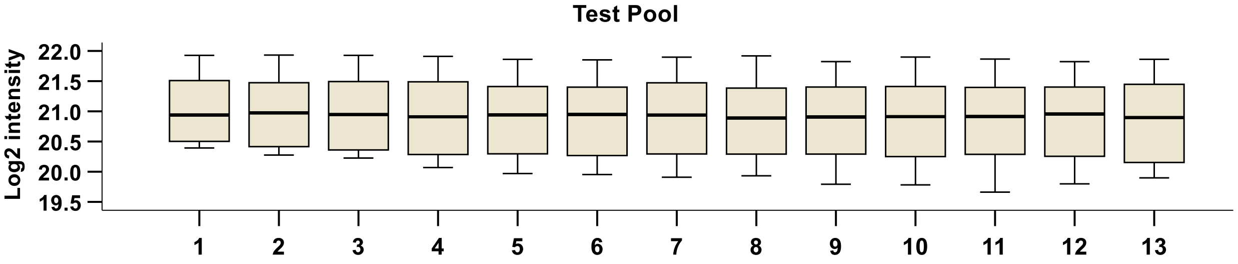

Supplement: Supplemental data [file jciinsight-6-152109-s176.pdf]
