# Supplementary material for: Diminished retinal complex lipid synthesis and impaired fatty acid β-oxidation associated with human diabetic retinopathy
Source: JCI Insight. 2021 Oct 8;6(19):e152109. doi: 10.1172/jci.insight.152109 (PMC8525591; doi:10.1172/jci.insight.152109)
Supplement: ICMJE disclosure forms [file jciinsight-6-152109-s178.pdf]

ICMJE DISCLOSURE FORM

Date: 08/01/2021
Your Name: Farsad Afshinnia
Manuscript Title: Diminished retinal complex lipid synthesis and impaired fatty acid β-oxidation associated with human diabetic retinopathy
Manuscript number (if known): 152109-INS-CMED-RV-2

In the interest of transparency, we ask you to disclose all relationships/activities/interests listed below that are related to the content of your manuscript. "Related" means any relation with for-profit or not-for-profit third parties whose interests may be affected by the content of the manuscript. Disclosure represents a commitment to transparency and does not necessarily indicate a bias. If you are in doubt about whether to list a relationship/activity/interest, it is preferable that you do so.

The following questions apply to the author's relationships/activities/interests as they relate to the current manuscript only.

The author's relationships/activities/interests should be defined broadly. For example, if your manuscript pertains to the epidemiology of hypertension, you should declare all relationships with manufacturers of antihypertensive medication, even if that medication is not mentioned in the manuscript.

In item #1 below, report all support for the work reported in this manuscript without time limit. For all other items, the time frame for disclosure is the past 36 months.

		**Name all entities with whom you have this relationship or indicate none (add rows as needed)**	**Specifications/Comments (e.g., if payments were made to you or to your institution)**
		**Time frame: Since the initial planning of the work**	
1	All support for the present manuscript (e.g., funding, provision of study materials, medical writing, article processing charges, etc.) **No time limit for this item.**	____ None	Farsad Afshinnia was funded by K08DK106523, R03DK121941 grants from NIDDK, and is supported by the JDRF Center for Excellence grant 5-COE-2019-861-S-B.
		**Time frame: past 36 months**	
2	Grants or contracts from any entity (if not indicated in item #1 above).	__ **X** __ None	
3	Royalties or licenses	__ **X** __ None	

4	Consulting fees	__ **X** __ None	
5	Payment or honoraria for lectures, presentations, speakers bureaus, manuscript writing or educational events	__ **X** __ None	
6	Payment for expert testimony	__ **X** __ None	
7	Support for attending meetings and/or travel	__ **X** __ None	
8	Patents planned, issued or pending	__ **X** __ None	
9	Participation on a Data Safety Monitoring Board or Advisory Board	__ **X** __ None	
10	Leadership or fiduciary role in other board, society, committee or advocacy group, paid or unpaid	__ **X** __ None	
11	Stock or stock options	__ **X** __ None	
12	Receipt of equipment, materials, drugs, medical writing, gifts or other services	__ **X** __ None	
13	Other financial or non-financial interests	__ **X** __ None	

Please place an "X" next to the following statement to indicate your agreement:

___ **I certify that I have answered every question and have not altered the wording of any of the questions on this form.**

ICMJE DISCLOSURE FORM

Date: 08/01/2021
Your Name: Subramaniam Pennathur
Manuscript Title: Diminished retinal complex lipid synthesis and impaired fatty acid β-oxidation associated with human diabetic retinopathy
Manuscript number (if known): 152109-INS-CMED-RV-2

In the interest of transparency, we ask you to disclose all relationships/activities/interests listed below that are related to the content of your manuscript. "Related" means any relation with for-profit or not-for-profit third parties whose interests may be affected by the content of the manuscript. Disclosure represents a commitment to transparency and does not necessarily indicate a bias. If you are in doubt about whether to list a relationship/activity/interest, it is preferable that you do so.

The following questions apply to the author's relationships/activities/interests as they relate to the current manuscript only.

The author's relationships/activities/interests should be defined broadly. For example, if your manuscript pertains to the epidemiology of hypertension, you should declare all relationships with manufacturers of antihypertensive medication, even if that medication is not mentioned in the manuscript.

In item #1 below, report all support for the work reported in this manuscript without time limit. For all other items, the time frame for disclosure is the past 36 months.

		**Name all entities with whom you have this relationship or indicate none (add rows as needed)**	**Specifications/Comments (e.g., if payments were made to you or to your institution)**
		**Time frame: Since the initial planning of the work**	
1	All support for the present manuscript (e.g., funding, provision of study materials, medical writing, article processing charges, etc.) **No time limit for this item.**	__ **X** __ None	
		**Time frame: past 36 months**	
2	Grants or contracts from any entity (if not indicated in item #1 above).	__ **X** __ None	
3	Royalties or licenses	__ **X** __ None	

4	Consulting fees	__ **X** __ None	
5	Payment or honoraria for lectures, presentations, speakers bureaus, manuscript writing or educational events	__ **X** __ None	
6	Payment for expert testimony	__ **X** __ None	
7	Support for attending meetings and/or travel	__ **X** __ None	
8	Patents planned, issued or pending	__ **X** __ None	
9	Participation on a Data Safety Monitoring Board or Advisory Board	__ **X** __ None	
10	Leadership or fiduciary role in other board, society, committee or advocacy group, paid or unpaid	__ **X** __ None	
11	Stock or stock options	__ **X** __ None	
12	Receipt of equipment, materials, drugs, medical writing, gifts or other services	__ **X** __ None	
13	Other financial or non-financial interests	__ **X** __ None	

Please place an "X" next to the following statement to indicate your agreement:

___ I certify that I have answered every question and have not altered the wording of any of the questions on this form.

ICMJE DISCLOSURE FORM

Date: 08/01/2021
Your Name: Patrice E. Fort
Manuscript Title: Diminished retinal complex lipid synthesis and impaired fatty acid β-oxidation associated with human diabetic retinopathy
Manuscript number (if known): 152109-INS-CMED-RV-2

In the interest of transparency, we ask you to disclose all relationships/activities/interests listed below that are related to the content of your manuscript. "Related" means any relation with for-profit or not-for-profit third parties whose interests may be affected by the content of the manuscript. Disclosure represents a commitment to transparency and does not necessarily indicate a bias. If you are in doubt about whether to list a relationship/activity/interest, it is preferable that you do so.

The following questions apply to the author's relationships/activities/interests as they relate to the current manuscript only.

The author's relationships/activities/interests should be defined broadly. For example, if your manuscript pertains to the epidemiology of hypertension, you should declare all relationships with manufacturers of antihypertensive medication, even if that medication is not mentioned in the manuscript.

In item #1 below, report all support for the work reported in this manuscript without time limit. For all other items, the time frame for disclosure is the past 36 months.

		**Name all entities with whom you have this relationship or indicate none (add rows as needed)**	**Specifications/Comments (e.g., if payments were made to you or to your institution)**
**Time frame: Since the initial planning of the work**			
1	All support for the present manuscript (e.g., funding, provision of study materials, medical writing, article processing charges, etc.) **No time limit for this item.**	Thomas Beatson Foundation	Research grant
		The Juvenile Diabetes Research Foundation	Research grant
**Time frame: past 36 months**			
2	Grants or contracts from any entity (if not indicated in item #1 above).	__ **X** __ None	
3	Royalties or licenses	__ **X** __ None	

4	Consulting fees	__ **X** __ None	
5	Payment or honoraria for lectures, presentations, speakers bureaus, manuscript writing or educational events	__ **X** __ None	
6	Payment for expert testimony	__ **X** __ None	
7	Support for attending meetings and/or travel	__ **X** __ None	
8	Patents planned, issued or pending	__ **X** __ None	
9	Participation on a Data Safety Monitoring Board or Advisory Board	__ **X** __ None	
10	Leadership or fiduciary role in other board, society, committee or advocacy group, paid or unpaid	__ **X** __ None	
11	Stock or stock options	__ **X** __ None	
12	Receipt of equipment, materials, drugs, medical writing, gifts or other services	__ **X** __ None	
13	Other financial or non-financial interests	__ **X** __ None	

Please place an "X" next to the following statement to indicate your agreement:

_X_ I certify that I have answered every question and have not altered the wording of any of the questions on this form.

ICMJE DISCLOSURE FORM

Date: August 1, 2021
Your Name: Robert G Nelson
Manuscript Title: Diminished retinal complex lipid synthesis and impaired fatty acid β-oxidation associated with human diabetic retinopathy
Manuscript number (if known): 152109-INS-CMED-RV-2

In the interest of transparency, we ask you to disclose all relationships/activities/interests listed below that are related to the content of your manuscript. "Related" means any relation with for-profit or not-for-profit third parties whose interests may be affected by the content of the manuscript. Disclosure represents a commitment to transparency and does not necessarily indicate a bias. If you are in doubt about whether to list a relationship/activity/interest, it is preferable that you do so.

The following questions apply to the author's relationships/activities/interests as they relate to the current manuscript only.

The author's relationships/activities/interests should be defined broadly. For example, if your manuscript pertains to the epidemiology of hypertension, you should declare all relationships with manufacturers of antihypertensive medication, even if that medication is not mentioned in the manuscript.

In item #1 below, report all support for the work reported in this manuscript without time limit. For all other items, the time frame for disclosure is the past 36 months.

		**Name all entities with whom you have this relationship or indicate none (add rows as needed)**	**Specifications/Comments (e.g., if payments were made to you or to your institution)**
		**Time frame: Since the initial planning of the work**	
1	All support for the present manuscript (e.g., funding, provision of study materials, medical writing, article processing charges, etc.) **No time limit for this item.**	____ None<br>NIDDK	Intramural support.
		ADA	Grant support to institution.
		**Time frame: past 36 months**	
2	Grants or contracts from any entity (if not indicated in item #1 above).	__X__ None	
3	Royalties or licenses	__X__ None	

4	Consulting fees	__X__ None	
5	Payment or honoraria for lectures, presentations, speakers bureaus, manuscript writing or educational events	__X__ None	
6	Payment for expert testimony	__X__ None	
7	Support for attending meetings and/or travel	__X__ None	
8	Patents planned, issued or pending	__X__ None	
9	Participation on a Data Safety Monitoring Board or Advisory Board	__X__ None	
10	Leadership or fiduciary role in other board, society, committee or advocacy group, paid or unpaid	__X__ None	
11	Stock or stock options	__X__ None	
12	Receipt of equipment, materials, drugs, medical writing, gifts or other services	__X__ None	
13	Other financial or non-financial interests	__X__ None	

Please place an "X" next to the following statement to indicate your agreement:

_X_ I certify that I have answered every question and have not altered the wording of any of the questions on this form.

ICMJE DISCLOSURE FORM

Date: 08/01/21
Your Name: Steven F. Abcouwer, Ph.D.
Manuscript Title: Diminished retinal complex lipid synthesis and impaired fatty acid β-oxidation associated with human diabetic retinopathy
Manuscript number (if known): 152109-INS-CMED-RV-2

In the interest of transparency, we ask you to disclose all relationships/activities/interests listed below that are related to the content of your manuscript. "Related" means any relation with for-profit or not-for-profit third parties whose interests may be affected by the content of the manuscript. Disclosure represents a commitment to transparency and does not necessarily indicate a bias. If you are in doubt about whether to list a relationship/activity/interest, it is preferable that you do so.

The following questions apply to the author's relationships/activities/interests as they relate to the current manuscript only.

The author's relationships/activities/interests should be defined broadly. For example, if your manuscript pertains to the epidemiology of hypertension, you should declare all relationships with manufacturers of antihypertensive medication, even if that medication is not mentioned in the manuscript.

In item #1 below, report all support for the work reported in this manuscript without time limit. For all other items, the time frame for disclosure is the past 36 months.

		**Name all entities with whom you have this relationship or indicate none (add rows as needed)**	**Specifications/Comments (e.g., if payments were made to you or to your institution)**
**Time frame: Since the initial planning of the work**			
1	All support for the present manuscript (e.g., funding, provision of study materials, medical writing, article processing charges, etc.) **No time limit for this item.**	__ **X** __ None	
**Time frame: past 36 months**			
2	Grants or contracts from any entity (if not indicated in item #1 above).	__ **X** __ None	
3	Royalties or licenses	__ **X** __ None	

4	Consulting fees	__ **X** __ None	
5	Payment or honoraria for lectures, presentations, speakers bureaus, manuscript writing or educational events	__ **X** __ None	
6	Payment for expert testimony	__ **X** __ None	
7	Support for attending meetings and/or travel	__ **X** __ None	
8	Patents planned, issued or pending	__ **X** __ None	
9	Participation on a Data Safety Monitoring Board or Advisory Board	__ **X** __ None	
10	Leadership or fiduciary role in other board, society, committee or advocacy group, paid or unpaid	__ **X** __ None	
11	Stock or stock options	__ **X** __ None	
12	Receipt of equipment, materials, drugs, medical writing, gifts or other services	__ **X** __ None	
13	Other financial or non-financial interests	__ **X** __ None	

Please place an "X" next to the following statement to indicate your agreement:

X I certify that I have answered every question and have not altered the wording of any of the questions on this form.

ICMJE DISCLOSURE FORM

Date: 08/01/21
Your Name: Jaeman Byun
Manuscript Title: Diminished retinal complex lipid synthesis and impaired fatty acid β-oxidation associated with human diabetic retinopathy
Manuscript number (if known): 152109-INS-CMED-RV-2

In the interest of transparency, we ask you to disclose all relationships/activities/interests listed below that are related to the content of your manuscript. "Related" means any relation with for-profit or not-for-profit third parties whose interests may be affected by the content of the manuscript. Disclosure represents a commitment to transparency and does not necessarily indicate a bias. If you are in doubt about whether to list a relationship/activity/interest, it is preferable that you do so.

The following questions apply to the author's relationships/activities/interests as they relate to the current manuscript only.

The author's relationships/activities/interests should be defined broadly. For example, if your manuscript pertains to the epidemiology of hypertension, you should declare all relationships with manufacturers of antihypertensive medication, even if that medication is not mentioned in the manuscript.

In item #1 below, report all support for the work reported in this manuscript without time limit. For all other items, the time frame for disclosure is the past 36 months.

		**Name all entities with whom you have this relationship or indicate none (add rows as needed)**	**Specifications/Comments (e.g., if payments were made to you or to your institution)**
		**Time frame: Since the initial planning of the work**	
1	All support for the present manuscript (e.g., funding, provision of study materials, medical writing, article processing charges, etc.) **No time limit for this item.**	__ **X** __ None	
		**Time frame: past 36 months**	
2	Grants or contracts from any entity (if not indicated in item #1 above).	__ **X** __ None	
3	Royalties or licenses	__ **X** __ None	

4	Consulting fees	__ **X** __ None	
5	Payment or honoraria for lectures, presentations, speakers bureaus, manuscript writing or educational events	__ **X** __ None	
6	Payment for expert testimony	__ **X** __ None	
7	Support for attending meetings and/or travel	__ **X** __ None	
8	Patents planned, issued or pending	__ **X** __ None	
9	Participation on a Data Safety Monitoring Board or Advisory Board	__ **X** __ None	
10	Leadership or fiduciary role in other board, society, committee or advocacy group, paid or unpaid	__ **X** __ None	
11	Stock or stock options	__ **X** __ None	
12	Receipt of equipment, materials, drugs, medical writing, gifts or other services	__ **X** __ None	
13	Other financial or non-financial interests	__ **X** __ None	

Please place an "X" next to the following statement to indicate your agreement:

_x__ I certify that I have answered every question and have not altered the wording of any of the questions on this form.

ICMJE DISCLOSURE FORM

Date: 8/2/2021

Your Name: Thekkelnaycke Rajendiran

Manuscript Title: Diminished retinal complex lipid synthesis and impaired fatty acid β-oxidation associated with human diabetic retinopathy
Manuscript number (if known): 152109-INS-CMED-RV-2

In the interest of transparency, we ask you to disclose all relationships/activities/interests listed below that are related to the content of your manuscript. "Related" means any relation with for-profit or not-for-profit third parties whose interests may be affected by the content of the manuscript. Disclosure represents a commitment to transparency and does not necessarily indicate a bias. If you are in doubt about whether to list a relationship/activity/interest, it is preferable that you do so.

The following questions apply to the author's relationships/activities/interests as they relate to the current manuscript only.

The author's relationships/activities/interests should be defined broadly. For example, if your manuscript pertains to the epidemiology of hypertension, you should declare all relationships with manufacturers of antihypertensive medication, even if that medication is not mentioned in the manuscript.

In item #1 below, report all support for the work reported in this manuscript without time limit. For all other items, the time frame for disclosure is the past 36 months.

		**Name all entities with whom you have this relationship or indicate none (add rows as needed)**	**Specifications/Comments (e.g., if payments were made to you or to your institution)**
		**Time frame: Since the initial planning of the work**	
1	All support for the present manuscript (e.g., funding, provision of study materials, medical writing, article processing charges, etc.) **No time limit for this item.**	_x___ None	
		**Time frame: past 36 months**	
2	Grants or contracts from any entity (if not indicated in item #1 above).	__x__ None	
3	Royalties or licenses	__x__ None	

4	Consulting fees	__x__ None	
5	Payment or honoraria for lectures, presentations, speakers bureaus, manuscript writing or educational events	__x__ None	
6	Payment for expert testimony	_x___ None	
7	Support for attending meetings and/or travel	_x___ None	
8	Patents planned, issued or pending	__x__ None	
9	Participation on a Data Safety Monitoring Board or Advisory Board	__x__ None	
10	Leadership or fiduciary role in other board, society, committee or advocacy group, paid or unpaid	_x___ None	
11	Stock or stock options	_x___ None	
12	Receipt of equipment, materials, drugs, medical writing, gifts or other services	__x__ None	
13	Other financial or non-financial interests	__x__ None	

Please place an "X" next to the following statement to indicate your agreement:

__x_ I certify that I have answered every question and have not altered the wording of any of the questions on this form.

ICMJE DISCLOSURE FORM

Date: 8/2/2021
Your Name: Yang Shan
Manuscript Title: Diminished retinal complex lipid synthesis and impaired fatty acid β-oxidation associated with human diabetic retinopathy
Manuscript number (if known): 152109-INS-CMED-RV-2

In the interest of transparency, we ask you to disclose all relationships/activities/interests listed below that are related to the content of your manuscript. "Related" means any relation with for-profit or not-for-profit third parties whose interests may be affected by the content of the manuscript. Disclosure represents a commitment to transparency and does not necessarily indicate a bias. If you are in doubt about whether to list a relationship/activity/interest, it is preferable that you do so.

The following questions apply to the author's relationships/activities/interests as they relate to the current manuscript only.

The author's relationships/activities/interests should be defined broadly. For example, if your manuscript pertains to the epidemiology of hypertension, you should declare all relationships with manufacturers of antihypertensive medication, even if that medication is not mentioned in the manuscript.

In item #1 below, report all support for the work reported in this manuscript without time limit. For all other items, the time frame for disclosure is the past 36 months.

		**Name all entities with whom you have this relationship or indicate none (add rows as needed)**	**Specifications/Comments (e.g., if payments were made to you or to your institution)**
**Time frame: Since the initial planning of the work**			
1	All support for the present manuscript (e.g., funding, provision of study materials, medical writing, article processing charges, etc.) **No time limit for this item.**	__x_ None	
**Time frame: past 36 months**			
2	Grants or contracts from any entity (if not indicated in item #1 above).	_x__ None	
3	Royalties or licenses	__x_ None	

4	Consulting fees	__x_ None	
5	Payment or honoraria for lectures, presentations, speakers bureaus, manuscript writing or educational events	__x_ None	
6	Payment for expert testimony	__x_ None	
7	Support for attending meetings and/or travel	__x_ None	
8	Patents planned, issued or pending	__x_ None	
9	Participation on a Data Safety Monitoring Board or Advisory Board	_x__ None	
10	Leadership or fiduciary role in other board, society, committee or advocacy group, paid or unpaid	__x_ None	
11	Stock or stock options	__x_ None	
12	Receipt of equipment, materials, drugs, medical writing, gifts or other services	__x_ None	
13	Other financial or non-financial interests	__x_ None	

Please place an "X" next to the following statement to indicate your agreement:

_x_ I certify that I have answered every question and have not altered the wording of any of the questions on this form.

ICMJE DISCLOSURE FORM

Date: 08/02/2021
Your Name: Jerome E Roger
Manuscript Title: Diminished retinal complex lipid synthesis and impaired fatty acid β-oxidation associated with human diabetic retinopathy
Manuscript number (if known): 152109-INS-CMED-RV-2

In the interest of transparency, we ask you to disclose all relationships/activities/interests listed below that are related to the content of your manuscript. "Related" means any relation with for-profit or not-for-profit third parties whose interests may be affected by the content of the manuscript. Disclosure represents a commitment to transparency and does not necessarily indicate a bias. If you are in doubt about whether to list a relationship/activity/interest, it is preferable that you do so.

The following questions apply to the author's relationships/activities/interests as they relate to the current manuscript only.

The author's relationships/activities/interests should be defined broadly. For example, if your manuscript pertains to the epidemiology of hypertension, you should declare all relationships with manufacturers of antihypertensive medication, even if that medication is not mentioned in the manuscript.

In item #1 below, report all support for the work reported in this manuscript without time limit. For all other items, the time frame for disclosure is the past 36 months.

		Name all entities with whom you have this relationship or indicate none (add rows as needed)	Specifications/Comments (e.g., if payments were made to you or to your institution)
		**Time frame: Since the initial planning of the work**	
1	All support for the present manuscript (e.g., funding, provision of study materials, medical writing, article processing charges, etc.) **No time limit for this item.**	__ **X** __ None	
		**Time frame: past 36 months**	
2	Grants or contracts from any entity (if not indicated in item #1 above).	__ **X** __ None	
3	Royalties or licenses	__ **X** __ None	
4	Consulting fees	__ **X** __ None	

5	Payment or honoraria for lectures, presentations, speakers bureaus, manuscript writing or educational events	__ **X** __ None	
6	Payment for expert testimony	__ **X** __ None	
7	Support for attending meetings and/or travel	__ **X** __ None	
8	Patents planned, issued or pending	__ **X** __ None	
9	Participation on a Data Safety Monitoring Board or Advisory Board	__ **X** __ None	
10	Leadership or fiduciary role in other board, society, committee or advocacy group, paid or unpaid	__ **X** __ None	
11	Stock or stock options	__ **X** __ None	
12	Receipt of equipment, materials, drugs, medical writing, gifts or other services	__ **X** __ None	
13	Other financial or non-financial interests	__ **X** __ None	

Please place an "X" next to the following statement to indicate your agreement:

X I certify that I have answered every question and have not altered the wording of any of the questions on this form.

ICMJE DISCLOSURE FORM

Date: August 2nd 2021
Your Name: Helen C Looker
Manuscript Title: Diminished retinal complex lipid synthesis and impaired fatty acid β-oxidation associated with human diabetic retinopathy
Manuscript number (if known): 152109-INS-CMED-RV-2

In the interest of transparency, we ask you to disclose all relationships/activities/interests listed below that are related to the content of your manuscript. "Related" means any relation with for-profit or not-for-profit third parties whose interests may be affected by the content of the manuscript. Disclosure represents a commitment to transparency and does not necessarily indicate a bias. If you are in doubt about whether to list a relationship/activity/interest, it is preferable that you do so.

The following questions apply to the author's relationships/activities/interests as they relate to the current manuscript only.

The author's relationships/activities/interests should be defined broadly. For example, if your manuscript pertains to the epidemiology of hypertension, you should declare all relationships with manufacturers of antihypertensive medication, even if that medication is not mentioned in the manuscript.

In item #1 below, report all support for the work reported in this manuscript without time limit. For all other items, the time frame for disclosure is the past 36 months.

		**Name all entities with whom you have this relationship or indicate none (add rows as needed)**	**Specifications/Comments (e.g., if payments were made to you or to your institution)**
		**Time frame: Since the initial planning of the work**	
1	All support for the present manuscript (e.g., funding, provision of study materials, medical writing, article processing charges, etc.) **No time limit for this item.**	_x__ None	
		**Time frame: past 36 months**	
2	Grants or contracts from any entity (if not indicated in item #1 above).	x__ None	
3	Royalties or licenses	_x__ None	

4	Consulting fees	_x_ None	
5	Payment or honoraria for lectures, presentations, speakers bureaus, manuscript writing or educational events	_x__ None	
6	Payment for expert testimony	_x__ None	
7	Support for attending meetings and/or travel	_x__ None	
8	Patents planned, issued or pending	_x___ None	
9	Participation on a Data Safety Monitoring Board or Advisory Board	_x___ None	
10	Leadership or fiduciary role in other board, society, committee or advocacy group, paid or unpaid	_x__ None	
11	Stock or stock options	_x__ None	
12	Receipt of equipment, materials, drugs, medical writing, gifts or other services	_x__ None	
13	Other financial or non-financial interests	_x__ None	

Please place an "X" next to the following statement to indicate your agreement:

_x_ I certify that I have answered every question and have not altered the wording of any of the questions on this form.

ICMJE DISCLOSURE FORM

Date: 08/02/2021
Your Name: Tanu Soni
Manuscript Title: Diminished retinal complex lipid synthesis and impaired fatty acid β-oxidation associated with human diabetic retinopathy
Manuscript number (if known): 152109-INS-CMED-RV-2

In the interest of transparency, we ask you to disclose all relationships/activities/interests listed below that are related to the content of your manuscript. "Related" means any relation with for-profit or not-for-profit third parties whose interests may be affected by the content of the manuscript. Disclosure represents a commitment to transparency and does not necessarily indicate a bias. If you are in doubt about whether to list a relationship/activity/interest, it is preferable that you do so.

The following questions apply to the author's relationships/activities/interests as they relate to the current manuscript only.

The author's relationships/activities/interests should be defined broadly. For example, if your manuscript pertains to the epidemiology of hypertension, you should declare all relationships with manufacturers of antihypertensive medication, even if that medication is not mentioned in the manuscript.

In item #1 below, report all support for the work reported in this manuscript without time limit. For all other items, the time frame for disclosure is the past 36 months.

		**Name all entities with whom you have this relationship or indicate none (add rows as needed)**	**Specifications/Comments (e.g., if payments were made to you or to your institution)**
		**Time frame: Since the initial planning of the work**	
1	All support for the present manuscript (e.g., funding, provision of study materials, medical writing, article processing charges, etc.) **No time limit for this item.**	_x__ None	
		**Time frame: past 36 months**	
2	Grants or contracts from any entity (if not indicated in item #1 above).	x__ None	
3	Royalties or licenses	_x__ None	

4	Consulting fees	_x_ None	
5	Payment or honoraria for lectures, presentations, speakers bureaus, manuscript writing or educational events	_x__ None	
6	Payment for expert testimony	_x__ None	
7	Support for attending meetings and/or travel	_x__ None	
8	Patents planned, issued or pending	_x___ None	
9	Participation on a Data Safety Monitoring Board or Advisory Board	_x___ None	
10	Leadership or fiduciary role in other board, society, committee or advocacy group, paid or unpaid	_x__ None	
11	Stock or stock options	_x__ None	
12	Receipt of equipment, materials, drugs, medical writing, gifts or other services	_x__ None	
13	Other financial or non-financial interests	_x__ None	

Please place an "X" next to the following statement to indicate your agreement:

_x_ I certify that I have answered every question and have not altered the wording of any of the questions on this form.

ICMJE DISCLOSURE FORM

Date: 8/3/21
Your Name: Thomas W. Gardner
Manuscript Title: Diminished retinal complex lipid synthesis and impaired fatty acid β-oxidation associated with human diabetic retinopathy
Manuscript number (if known): 152109-INS-CMED-RV-2

In the interest of transparency, we ask you to disclose all relationships/activities/interests listed below that are related to the content of your manuscript. "Related" means any relation with for-profit or not-for-profit third parties whose interests may be affected by the content of the manuscript. Disclosure represents a commitment to transparency and does not necessarily indicate a bias. If you are in doubt about whether to list a relationship/activity/interest, it is preferable that you do so.

The following questions apply to the author's relationships/activities/interests as they relate to the current manuscript only.

The author's relationships/activities/interests should be defined broadly. For example, if your manuscript pertains to the epidemiology of hypertension, you should declare all relationships with manufacturers of antihypertensive medication, even if that medication is not mentioned in the manuscript.

In item #1 below, report all support for the work reported in this manuscript without time limit. For all other items, the time frame for disclosure is the past 36 months.

		**Name all entities with whom you have this relationship or indicate none (add rows as needed)**	**Specifications/Comments (e.g., if payments were made to you or to your institution)**
		**Time frame: Since the initial planning of the work**	
1	All support for the present manuscript (e.g., funding, provision of study materials, medical writing, article processing charges, etc.) **No time limit for this item.**	JDRF Center of Excellence (5-COE-2019-861-S-B)	
		**Time frame: past 36 months**	
2	Grants or contracts from any entity (if not indicated in item #1 above).	Taubman Medical Research Institute	
		R01EY20852	
		- R01 EY013961<br>- R24 DK082841<br>- ORA, Inc. A Randomized, Double-Masked, 48-Week, Parallel-Group, Placebo-	

		Controlled, Proof-of-Concept Study to Investigate the Efficacy and Safety of RG7774 in Patients with Diabetes Mellitus Type 1 or Type 2 with Treatment-Naive Diabetic Retinopathy<br><br>- Coulter Translational Research Program<br><br>- JDRF (1-RSC-2021-1051-A-N)<br>- Novo Nordisk Foundation	
3	Royalties or licenses	__x__ None	
4	Consulting fees	National Medical Research Council, Singapore, 2021	To me
5	Payment or honoraria for lectures, presentations, speakers bureaus, manuscript writing or educational events	____ None	
6	Payment for expert testimony	__x__ None	
7	Support for attending meetings and/or travel	__ __ None	
		Lions Eye Institute for Transplant and Research	To me
8	Patents planned, issued or pending	____ None<br>Ojeda, LV, Sundstrom JM, Holmer JT, Cruz A, Bhamni A, Demirci H, Gardner TW. Devices, Systems and Methods for Biological Sample Collection. PCT/US20/33995 submitted 5.21.20	n/a

9	Participation on a Data Safety Monitoring Board or Advisory Board	____ None<br>Ophthalmology Working Group of DCCT/EDIC	
10	Leadership or fiduciary role in other board, society, committee or advocacy group, paid or unpaid	__x__ None	
11	Stock or stock options	__x__ None	
12	Receipt of equipment, materials, drugs, medical writing, gifts or other services	__x__ None	
13	Other financial or non-financial interests	____ None	

Please place an "X" next to the following statement to indicate your agreement:

___ I certify that I have answered every question and have not altered the wording of any of the questions on this form.

ICMJE DISCLOSURE FORM

Date: 08/02/2021
Your Name: George Michailidis
Manuscript Title: Diminished retinal complex lipid synthesis and impaired fatty acid β-oxidation associated with human diabetic retinopathy
Manuscript number (if known): 152109-INS-CMED-RV-2

In the interest of transparency, we ask you to disclose all relationships/activities/interests listed below that are related to the content of your manuscript. "Related" means any relation with for-profit or not-for-profit third parties whose interests may be affected by the content of the manuscript. Disclosure represents a commitment to transparency and does not necessarily indicate a bias. If you are in doubt about whether to list a relationship/activity/interest, it is preferable that you do so.

The following questions apply to the author's relationships/activities/interests as they relate to the current manuscript only.

The author's relationships/activities/interests should be defined broadly. For example, if your manuscript pertains to the epidemiology of hypertension, you should declare all relationships with manufacturers of antihypertensive medication, even if that medication is not mentioned in the manuscript.

In item #1 below, report all support for the work reported in this manuscript without time limit. For all other items, the time frame for disclosure is the past 36 months.

		**Name all entities with whom you have this relationship or indicate none (add rows as needed)**	**Specifications/Comments (e.g., if payments were made to you or to your institution)**
**Time frame: Since the initial planning of the work**			
1	All support for the present manuscript (e.g., funding, provision of study materials, medical writing, article processing charges, etc.) **No time limit for this item.**	_x__ None	
**Time frame: past 36 months**			
2	Grants or contracts from any entity (if not indicated in item #1 above).	x__ None	
3	Royalties or licenses	_x__ None	

4	Consulting fees	_x_ None	
5	Payment or honoraria for lectures, presentations, speakers bureaus, manuscript writing or educational events	_x__ None	
6	Payment for expert testimony	_x__ None	
7	Support for attending meetings and/or travel	_x__ None	
8	Patents planned, issued or pending	_x___ None	
9	Participation on a Data Safety Monitoring Board or Advisory Board	_x___ None	
10	Leadership or fiduciary role in other board, society, committee or advocacy group, paid or unpaid	_x__ None	
11	Stock or stock options	_x__ None	
12	Receipt of equipment, materials, drugs, medical writing, gifts or other services	_x__ None	
13	Other financial or non-financial interests	_x__ None	

Please place an "X" next to the following statement to indicate your agreement:

_x_ I certify that I have answered every question and have not altered the wording of any of the questions on this form.

ICMJE DISCLOSURE FORM

Date: 08/02/2021
Your Name: Matthias Kretzler
Manuscript Title: Diminished retinal complex lipid synthesis and impaired fatty acid β-oxidation associated with human diabetic retinopathy
Manuscript number (if known): 152109-INS-CMED-RV-2

In the interest of transparency, we ask you to disclose all relationships/activities/interests listed below that are related to the content of your manuscript. "Related" means any relation with for-profit or not-for-profit third parties whose interests may be affected by the content of the manuscript. Disclosure represents a commitment to transparency and does not necessarily indicate a bias. If you are in doubt about whether to list a relationship/activity/interest, it is preferable that you do so.

The following questions apply to the author's relationships/activities/interests as they relate to the current manuscript only.

The author's relationships/activities/interests should be defined broadly. For example, if your manuscript pertains to the epidemiology of hypertension, you should declare all relationships with manufacturers of antihypertensive medication, even if that medication is not mentioned in the manuscript.

In item #1 below, report all support for the work reported in this manuscript without time limit. For all other items, the time frame for disclosure is the past 36 months.

		**Name all entities with whom you have this relationship or indicate none (add rows as needed)**	**Specifications/Comments (e.g., if payments were made to you or to your institution)**
		**Time frame: Since the initial planning of the work**	
1	All support for the present manuscript (e.g., funding, provision of study materials, medical writing, article processing charges, etc.) **No time limit for this item.**	_x__ None	
		**Time frame: past 36 months**	
2	Grants or contracts from any entity (if not indicated in item #1 above).	x__ None	
3	Royalties or licenses	_x__ None	

4	Consulting fees	_x_ None	
5	Payment or honoraria for lectures, presentations, speakers bureaus, manuscript writing or educational events	_x__ None	
6	Payment for expert testimony	_x__ None	
7	Support for attending meetings and/or travel	_x__ None	
8	Patents planned, issued or pending	_x___ None	
9	Participation on a Data Safety Monitoring Board or Advisory Board	_x___ None	
10	Leadership or fiduciary role in other board, society, committee or advocacy group, paid or unpaid	_x__ None	
11	Stock or stock options	_x__ None	
12	Receipt of equipment, materials, drugs, medical writing, gifts or other services	_x__ None	
13	Other financial or non-financial interests	_x__ None	

Please place an "X" next to the following statement to indicate your agreement:

_x_ I certify that I have answered every question and have not altered the wording of any of the questions on this form.
